# The Crucial Role of the Blood–Brain Barrier in Neurodegenerative Diseases: Mechanisms of Disruption and Therapeutic Implications

**DOI:** 10.3390/jcm14020386

**Published:** 2025-01-09

**Authors:** Sehwan Kim, Un Ju Jung, Sang Ryong Kim

**Affiliations:** 1 School of Life Science and Biotechnology, Kyungpook National University, Daegu 41566, Republic of Korea; arputa@naver.com; 2 BK21 FOUR KNU Creative BioResearch Group, Kyungpook National University, Daegu 41566, Republic of Korea; 3 Department of Food Science and Nutrition, Pukyong National University, Busan 48513, Republic of Korea; jungunju@naver.com; 4 Brain Science and Engineering Institute, Kyungpook National University, Daegu 41404, Republic of Korea

**Keywords:** blood–brain barrier, neurodegenerative diseases, oxidative stress, neuroinflammation, neurotoxicity

## Abstract

The blood–brain barrier (BBB) is a crucial structure that maintains brain homeostasis by regulating the entry of molecules and cells from the bloodstream into the central nervous system (CNS). Neurodegenerative diseases such as Alzheimer’s and Parkinson’s disease, as well as ischemic stroke, compromise the integrity of the BBB. This leads to increased permeability and the infiltration of harmful substances, thereby accelerating neurodegeneration. In this review, we explore the mechanisms underlying BBB disruption, including oxidative stress, neuroinflammation, vascular dysfunction, and the loss of tight junction integrity, in patients with neurodegenerative diseases. We discuss how BBB breakdown contributes to neuroinflammation, neurotoxicity, and the abnormal accumulation of pathological proteins, all of which exacerbate neuronal damage and facilitate disease progression. Furthermore, we discuss potential therapeutic strategies aimed at preserving or restoring BBB function, such as anti-inflammatory treatments, antioxidant therapies, and approaches to enhance tight junction integrity. Given the central role of the BBB in neurodegeneration, maintaining its integrity represents a promising therapeutic approach to slow or prevent the progression of neurodegenerative diseases.

## 1. Introduction

Neurodegenerative diseases, such as Alzheimer’s disease (AD) and Parkinson’s disease (PD), as well as ischemic stroke, represent a growing public health concern because of their association with progressive neuronal loss, cognitive decline, and motor dysfunction [1,2,3]. While the underlying causes of these diseases are complex and multifactorial, the breakdown of the blood–brain barrier (BBB) is a crucial and increasingly recognized element involved in their pathogenesis [4,5,6]. The BBB is a highly specialized and selectively permeable structure that is essential for maintaining the brain’s microenvironment by regulating the transport of molecules between the bloodstream and the brain [7,8,9]. It not only supports brain homeostasis but also protects neural tissue from potentially harmful agents, toxins, and inflammatory cells [9,10,11].

However, in neurodegenerative diseases, the integrity of the BBB is frequently compromised. This allows molecules from the blood, including inflammatory cells, cytokines, and toxins, to infiltrate the brain parenchyma [6,12,13,14]. This infiltration exacerbates neuroinflammation, oxidative stress, and neuronal dysfunction, thereby accelerating the disease process [6,12,13,14]. Studies have shown that BBB disruption is both a consequence and cause of neurodegeneration, as increased permeability creates a pathological feedback loop in which neuronal injury exacerbates further BBB impairment [6,12,13,14]. Protection and restoration of the BBB can therefore serve as a crucial therapeutic target for neurodegenerative diseases [6,15,16]. Stabilizing the integrity of the BBB may help reduce the influx of harmful agents, mitigate neuroinflammation, and preserve neural function [6,11,12].

In this review, we aim to provide a detailed understanding of the mechanisms involved in BBB disruption in neurodegenerative diseases, explore the pathological impacts of this disruption, and discuss potential therapeutic strategies to protect and restore BBB integrity. By emphasizing the importance of maintaining a healthy BBB, we aim to highlight its role in mitigating the progression of neurodegenerative diseases.

## 2. Structure and Function of the BBB

**Figure 1:**
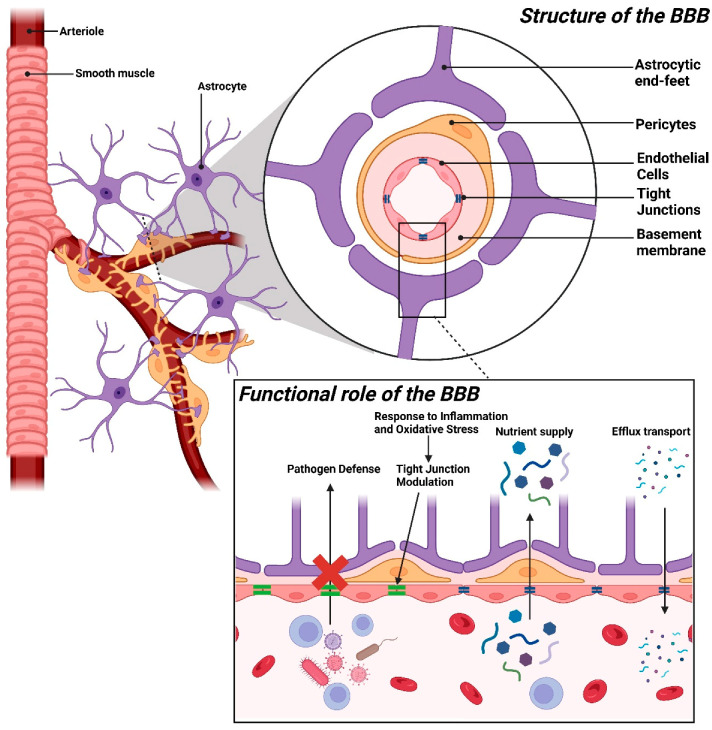
Structure and function of the BBB. The BBB is a specialized, selectively permeable interface that separates the brain from the systemic circulation. It plays a crucial role in maintaining the stability of the CNS by tightly regulating the exchange of substances and protecting neurons from toxins and inflammation. It consists of endothelial cells connected by tight junctions, pericytes embedded in the basement membrane, astrocytic endfeet, and an extracellular basement membrane, each uniquely contributing to its integrity and selective permeability. Endothelial cells form the core of the BBB, with unique tight junctions restricting paracellular movement and regulating substance passage. Pericytes support structural integrity, influence blood flow, and enhance tight junction stability. Astrocytic endfeet provide biochemical support, regulate ion and water homeostasis, and maintain neurotransmitter balance, while the basement membrane anchors BBB components and restricts immune cell infiltration, thereby protecting against inflammation. The BBB also supplies the brain with essential nutrients and removes metabolic waste, dynamically adapting to neural activity, oxidative stress, and hypoxia to support CNS homeostasis. These functions emphasize the importance of the BBB in maintaining CNS health and underscore the therapeutic potential of approaches that preserve or restore BBB integrity in neurodegenerative diseases. The figure was created using Biorender.com (Agreement number: CE27KUKTJR).

The BBB is a highly specialized, selectively permeable interface that separates the brain from the systemic circulation [9,11,17]. It primarily maintains a tightly regulated environment within the central nervous system (CNS) that is conducive to optimal neuronal function while shielding the brain from potential harm [9,11,17]. The BBB consists of a network of endothelial cells, pericytes, astrocytic endfeet, and a supporting basement membrane, each contributing uniquely to its integrity and selective permeability [18,19,20] (Figure 1).

### 2.1. Endothelial Cells and Tight Junctions

Endothelial cells serve as the primary structural and functional component of the BBB. They form a continuous monolayer along the cerebral microvasculature [17,18]. They are uniquely adapted to maintain BBB selectivity, which regulates the movement of substances between the bloodstream and the brain and is crucial for maintaining neural function [9,21]. Unlike peripheral endothelial cells, BBB endothelial cells exhibit specialized structural features, including unique tight junctions, low rates of transcytosis, and selective transport systems, which create a highly restrictive environment and ensure that only specific molecules necessary for brain health enter the CNS [21,22].

#### 2.1.1. Structure and Composition of Tight Junctions in BBB Endothelial Cells

BBB endothelial cells are joined by specialized tight junctions. This feature distinguishes them from peripheral endothelial cells [11,21]. These tight junctions are composed of a complex network of proteins, including claudins (particularly claudin-5), occludins, and junctional adhesion molecules (JAMs) [21,23]. These proteins form a highly resilient, nearly impermeable barrier that restricts the paracellular movement of ions, macromolecules, and other potentially harmful agents. This allows the BBB to tightly regulate the internal environment of the brain [21,24]. Claudin-5, one of the most abundant tight junction proteins in the BBB, is crucial for preventing the diffusion of small ions and solutes, thereby contributing to the high-resistance barrier unique to brain endothelial cells [23,25].

Tight junction proteins are further anchored to the endothelial cell cytoskeleton via zonula occludens (ZO) adaptor proteins, including ZO-1, ZO-2, and ZO-3. This connection provides structural stability to tight junctions and enables dynamic adjustments in response to physiological cues, such as neural activity or blood flow changes [21,23,24]. For example, ZO proteins can reorganize tight junctions in response to signaling molecules, such as cytokines, growth factors, and hormones, enabling the BBB to rapidly adapt to environmental or metabolic changes [26,27]. Consequently, the BBB is a highly organized and resilient barrier capable of withstanding fluctuations under both normal and pathological conditions [6,11,19].

#### 2.1.2. Communication with Other Cells and Response to the Neural Environment

BBB endothelial cells are not isolated in their function; they closely interact with pericytes, astrocytes, and microglia to form the neurovascular unit (NVU) [18,26,28]. This unit is a highly integrated system in which signaling molecules are continuously exchanged between these cells to maintain and regulate BBB integrity [18,29,30]. For instance, astrocytes release glial cell-derived neurotrophic factor (GDNF) and other signaling molecules that promote the expression and stability of tight junction proteins in endothelial cells [31,32,33]. Moreover, pericytes, which envelop endothelial cells on the abluminal side, release factors like transforming growth factor-beta (TGF-β) and platelet-derived growth factor-B (PDGF-B), further enhancing tight junction integrity and improving BBB stability [34,35,36].

The ability of endothelial cells to dynamically respond to the neural environment is vital for BBB homeostasis [18,21,37]. For example, in response to increased neural activity, endothelial cells can transiently alter tight junction permeability to enable a controlled increase in blood flow and nutrient supply to active brain regions; this process is closely linked to neurovascular coupling [38,39]. Additionally, endothelial cells can respond to inflammatory signals, oxidative stress, or hypoxia by modulating their tight junctions or activating efflux transporters, thereby protecting the brain from potential damage [40,41,42].

### 2.2. Pericytes

Pericytes are a vital cellular component of the BBB. They are embedded within the basement membrane that envelops the walls of brain capillaries [20,43,44]. These mural cells are situated closely along the abluminal side of endothelial cells and play a crucial role in maintaining BBB integrity, neurovascular regulation, and CNS homeostasis [44,45,46]. They can form physical contacts and communicate with endothelial cells via direct cell-to-cell interactions and paracrine signaling, thereby influencing the structural and functional properties of the BBB. Thus, they are crucial for maintaining brain health [44,47,48].

#### 2.2.1. Structural and Functional Support to the BBB

Pericytes maintain the structural integrity of the BBB by modulating the extracellular matrix (ECM) composition and supporting endothelial cell adhesion and alignment within the basement membrane [49,50,51]. Moreover, they secrete various ECM proteins, such as collagen, laminin, and fibronectin, which reinforce the physical barrier properties of the basement membrane and provide a stable scaffold for endothelial cells [34,52]. Through this interaction, pericytes help maintain vessel wall stability, thereby reducing the likelihood of BBB disruption under physiological stress or injury [34,52]. They are also essential for regulating cerebral blood flow (CBF) within the microvasculature [44,53,54]. They possess contractile properties mediated by contractile proteins, such as smooth muscle actin. Consequently, they can constrict or dilate capillaries, thereby modulating blood flow to specific brain regions in response to neuronal activity [44,46,55]. Through this process, known as neurovascular coupling, active brain regions can receive an adequate supply of oxygen and nutrients to support cognitive functions and overall brain health [56,57,58]. Pericyte dysfunction or loss can impair this regulation, leading to hypoperfusion and facilitating the progression of neurodegenerative diseases [34,52].

#### 2.2.2. Influence on Tight Junction Formation and Maintenance

In the BBB, pericytes play crucial roles in the formation, maturation, and maintenance of endothelial cell tight junctions [34,52]. Through signaling pathways, such as the TGF-β and Notch signaling pathways, pericytes promote the expression of tight junction proteins, including claudin-5, occludin, and JAMs, which are essential for forming a high-resistance barrier [24,29,59]. Moreover, pericytes secrete angiopoietin-1 and PDGF-B, which help stabilize tight junctions and strengthen BBB integrity [34,52]. In the absence of pericytes, endothelial cells exhibit increased permeability, enabling unregulated paracellular transport and compromising BBB function [34,60].

#### 2.2.3. Role in Response to Injury and Inflammation

In addition to their structural and regulatory functions, pericytes play crucial roles in CNS injury response and inflammation management, acting as the first responders to damage within the NVU [48,61,62]. They can detect injury or inflammatory signals and react by adjusting their phenotype to facilitate BBB repair and inflammation resolution [29,48,63]. Upon injury, pericytes release signaling molecules, such as cytokines and chemokines, which modulate endothelial and immune cell behaviors [48,64,65]. They can also differentiate into fibroblast-like cells to promote scar formation and wound repair around damaged blood vessels, thereby reinforcing BBB integrity in response to any stress or insult [66,67].

Pericytes can contribute to immune surveillance by regulating leukocyte transmigration across the BBB [68,69,70]. In neuroinflammatory conditions, pericytes can alter the expression of adhesion molecules to limit or facilitate the movement of immune cells into the CNS, providing an additional layer of immune control within the BBB [65,69,71]. However, in chronic inflammatory states associated with neurodegenerative diseases, prolonged activation of pericytes can lead to the excessive production of inflammatory mediators; this may exacerbate BBB breakdown and worsen disease pathology [35,62,68].

### 2.3. Astrocytic Endfeet

Astrocytes, a type of glial cell in the brain, extend their processes, known as astrocytic endfeet, to cover more than 90% of the capillary surface in the brain [32,72]. These endfeet play a crucial role in maintaining the structural and functional integrity of the BBB by providing biochemical support to endothelial cells [32,73,74]. By secreting growth factors, cytokines, and other signaling molecules, astrocytes regulate endothelial cell behavior and strengthen tight junctions [75,76,77]. Astrocytic endfeet also contribute to ion and water homeostasis, which is essential for maintaining the unique extracellular environment required for neuronal function [78].

#### 2.3.1. Support to Endothelial Cells and Maintenance of Tight Junctions

Astrocytes are vital for maintaining the structural stability of the BBB. Moreover, they closely interact with endothelial cells to modulate their function [31,74]. They release various growth factors, including GDNF, epidermal growth factor (EGF), and vascular endothelial growth factor (VEGF), which promote the survival, growth, and differentiation of endothelial cells [31,33,79]. These growth factors help endothelial cells stably express tight junction proteins (such as claudin-5, occludin, and JAMs), thereby enhancing cell adhesion and promoting BBB integrity [31,32,80].

Astrocytes also regulate the inflammatory response within the brain by releasing cytokines and chemokines that help endothelial cells respond to inflammatory conditions [75,81,82]. For instance, interleukin-6 (IL-6) modulates the reactivity of endothelial cells to inflammation, thereby minimizing BBB damage caused by immune responses [75,76,77]. Moreover, astrocytes secrete anti-inflammatory factors, such as TGF-β, which help protect endothelial cells from inflammatory damage and promote BBB stability [75,76,77].

#### 2.3.2. Regulation of Ion and Water Homeostasis

Astrocytic endfeet are essential for maintaining ion and water homeostasis in the brain [83,84,85]. They are particularly rich in aquaporin-4 (AQP4) water channels, which regulate water balance in the extracellular space [75,76,77]. AQP4 enables rapid water movement in response to brain swelling or inflammatory conditions, thereby alleviating edema and promoting BBB stability [77,84,86].

Astrocytes also play a crucial role in controlling potassium ion (K^+^) concentrations to maintain electrical stability within the brain [87,88]. When neuronal activity increases, local K^+^ concentrations increase. Astrocytes quickly absorb these excess ions to prevent excessive neuronal excitation [89,90]. This regulation helps maintain normal synaptic function and creates a stable extracellular environment for neurons [88,90].

#### 2.3.3. Metabolic Support and Energy Transfer

Astrocytes provide essential metabolic support to neurons by storing glucose as glycogen and breaking it down to lactate, which is then supplied to neurons as an energy source [91,92,93]. Lactate is a particularly important energy source for neurons during high activity periods [94,95,96]. This metabolic support helps sustain neuronal energy metabolism and promotes optimal neuronal function [92,93,95]. Astrocytes also participate in the glutamine–glutamate cycle to maintain neurotransmitter balance [97,98]. They absorb excess glutamate from the synaptic cleft and convert it to glutamine, which is then supplied to neurons [99,100,101]. This process prevents excitotoxicity from excessive excitatory neurotransmitters and ensures stable neurotransmission [100,102,103].

### 2.4. Basement Membrane

The basement membrane is a dense, specialized ECM that underlies the BBB. It plays a crucial role in maintaining the structural and functional integrity of the BBB [20,104]. The basement membrane is primarily composed of collagen (types IV and XVIII), laminin, fibronectin, heparan sulfate proteoglycans, and other glycoproteins. It provides essential support to BBB cells, anchoring endothelial cells and pericytes to form a cohesive, stable barrier [20,104,105].

#### 2.4.1. Structural Support and Stability

The basement membrane is not merely a passive support structure. Instead, it is an active component that contributes to the structural resilience and stability of the BBB [9,11,20]. It serves as a physical scaffold for endothelial cells and pericytes, allowing them to maintain their positions and form a tightly regulated barrier essential for CNS protection [9,19,20]. Laminin and collagen are key components of the basement membrane. They provide elasticity and tensile strength, enabling the BBB to withstand pressure fluctuations associated with blood flow while preserving its integrity [106,107,108]. This structural support is vital for maintaining tight junctions between endothelial cells, which are crucial for the selective permeability of the BBB [7,8,11].

#### 2.4.2. Biochemical Signaling and Cellular Interactions

The basement membrane also plays a crucial role in biochemical signaling [109,110,111]. It serves as a reservoir for growth factors, cytokines, and other signaling molecules that are released in response to physiological or pathological stimuli [112,113]. These signals influence the behavior of endothelial cells and pericytes, thereby regulating cell proliferation, migration, and survival [59,68,114]. For instance, the basement membrane releases VEGF and fibroblast growth factor (FGF), which promote endothelial cell survival and integrity, particularly in response to injury or stress [50,115,116]. Moreover, laminin and collagen in the basement membrane interact with integrins and other receptors on endothelial cells and pericytes, mediating cell adhesion and stabilizing the BBB [117,118]. This interaction is crucial for maintaining tight junction integrity. It provides a signaling pathway that reinforces tight junction proteins, such as claudin-5 and occludin, thereby enhancing the ability of the BBB to resist harmful substances [10,24,119].

#### 2.4.3. Barrier to Cellular Infiltration

The basement membrane mainly serves as a barrier to cellular infiltration [120,121,122]. In a healthy state, it restricts the migration of peripheral immune cells, such as leukocytes, preventing them from entering the CNS [123,124]. This selective restriction is crucial for protecting the CNS from potential inflammatory damage, as uncontrolled immune cell infiltration could cause neuroinflammation and compromise the BBB [6,125]. However, under pathological conditions, e.g., in neuroinflammatory or neurodegenerative diseases, the basement membrane becomes degraded. This degradation can lead to increased immune cell migration into the CNS, exacerbating tissue damage [126,127,128]. Matrix metalloproteinases (MMPs), which are upregulated in conditions like multiple sclerosis and AD, can degrade components of the basement membrane, such as collagen and laminin [129,130,131]. This degradation disrupts the structural integrity of the BBB and enables peripheral immune cells and inflammatory mediators to infiltrate brain tissue, contributing to further neurodegeneration and breakdown of the BBB [130,132].

#### 2.4.4. Role in Communication Within the NVU

The basement membrane also facilitates communication within the NVU, which includes endothelial cells, pericytes, astrocytes, and neurons [18,28,30]. It acts as a biochemical interface between these cell types, supporting their coordinated functions [18,28,30]. For example, astrocytic endfeet are anchored to the basement membrane. Here, they release signaling molecules, such as GDNF and TGF-β, which modulate the activity of endothelial cells and pericytes [32,133,134]. This signaling network within the NVU helps maintain BBB integrity and responds adaptively to neural activity, injury, or metabolic changes [38,135,136].

### 2.5. Functional Role of the BBB

Under normal physiological conditions, the BBB serves as a crucial defense mechanism that tightly regulates CNS homeostasis [8,11,137]. By acting as a highly selective barrier, it prevents harmful agents, such as pathogens, toxins, and peripheral immune cells, from entering the brain, thereby protecting neuronal tissue from damage and inflammation [8,126]. This selective permeability creates a stable environment that supports optimal neuronal function and overall brain health [138,139] (Table 1).

#### 2.5.1. Protection of the CNS and Selective Permeability

The BBB primarily shields the CNS from potentially harmful substances, maintaining an environment conducive to neural health [6,9,11]. Tight junctions between endothelial cells, combined with specialized transport systems, restrict the passage of pathogens, toxins, and other molecules, thereby minimizing the risk of infection and inflammation in the brain [9,24,140]. The BBB serves as a crucial protective mechanism for the CNS by acting as a selective barrier against circulating pathogens, such as bacteria and viruses [141,142]. Even during bloodstream infections, the BBB significantly reduces the likelihood of pathogens entering the brain, thereby lowering the risk of various infections, such as meningitis and encephalitis [143,144]. Pathogens typically manage to penetrate this barrier only in cases of severe infection or BBB disruption, highlighting the vital role of the BBB in disease prevention [141,142]. Moreover, the BBB restricts the access of peripheral immune cells, including T cells and macrophages, to prevent excessive immune responses that could otherwise lead to inflammation and neuronal damage [6,145]. This immune regulation is essential, as the brain’s neural tissue is highly sensitive to inflammation, which could disrupt neural circuits, impair synaptic signaling, and ultimately result in neuronal cell death if left unchecked [146,147].

#### 2.5.2. Nutrient Supply and Waste Removal

In addition to protecting the CNS from external threats, the BBB maintains the metabolic requirements of the brain [148]. It selectively allows the passage of necessary nutrients and ions. Moreover, it systematically removes metabolic waste products, thereby sustaining a stable environment for neuronal function [8,137,149].

The BBB plays an essential role in nutrient transport and waste removal, both of which are crucial for brain function [8,137,148]. Neurons and glial cells have high metabolic demands and rely on a steady supply of nutrients [150,151]. The BBB facilitates the transport of glucose, amino acids, and ions through specialized carriers and transport systems [152,153,154]. For example, glucose transporter-1 (GLUT1) selectively transports glucose, the primary energy source of the brain, across the BBB to provide a constant energy supply for ATP production and neurotransmitter synthesis [155,156,157]. Amino acids are essential for protein synthesis and neurotransmitter production. They cross the BBB via specific transporters, such as large neutral amino acid transporter 1 (LAT1), which can handle large neutral amino acids, including dopamine and serotonin precursors [154,158,159]. The BBB also tightly regulates ions, such as K^+^ and calcium ions, to maintain ionic stability and support synaptic transmission [6,160]. In addition to being involved in nutrient delivery, the BBB contains efflux transporters, such as ATP-binding cassette (ABC) transporters (e.g., P-glycoprotein [P-gp] and breast cancer resistance protein) that actively remove metabolic waste products, xenobiotics, and other potentially harmful substances [161,162,163]. This efflux system prevents toxin accumulation, thereby preserving a clean environment that supports neuronal health and reduces oxidative stress in the brain [164,165,166].

#### 2.5.3. Adaptive Regulation in Response to Neural Activity

The BBB is not a static barrier; it dynamically adjusts its permeability in response to the brain’s metabolic needs, neural activity, and environmental conditions [6,138,167]. This adaptability is essential for maintaining CNS homeostasis and enabling the brain to quickly respond to changes in activity and demand [8,138,168].

The BBB dynamically adapts to meet the brain’s demands during various conditions, such as increased neural activity, inflammation, oxidative stress, and hypoxia [165,169]. During heightened neural activity, such as intense cognitive processing or physical exertion, neurons require more oxygen and glucose [170,171,172]. Hence, the BBB increases blood flow to active brain regions through vasodilation and neurovascular coupling, thereby ensuring an adequate supply of these essential nutrients [57,173,174]. During inflammation or oxidative stress, the BBB can modulate tight junctions and enhance transporter activity to protect the CNS [6,24,175]. For example, it may strengthen tight junctions to prevent immune cells and inflammatory molecules from entering the brain, thereby reducing the risk of neuronal damage [57,173,174]. Moreover, during oxidative stress, the BBB may boost the activity of efflux transporters, such as P-gp, to expel toxic substances and lower oxidative load, thereby preserving neural integrity [176,177,178]. Under hypoxic conditions, the BBB can upregulate glucose and metabolite transporters to sustain neuronal energy levels. Additionally, it can enhance antioxidant defenses in endothelial cells to counteract the oxidative damage often associated with low oxygen levels [172,179,180]. These adaptive mechanisms collectively help maintain the internal environment of the brain and protect neural tissue from potential damage [6,138].

The BBB plays a fundamental role in CNS protection, nutrient supply, waste removal, and adaptive regulation in response to neural activity [6,138]. Its selective permeability is crucial for preventing the entry of harmful substances and immune cells, thereby maintaining a stable environment for neuronal health [6,138]. The BBB regulates the supply of nutrients and removal of metabolic waste, ensuring that neurons and glial cells receive the resources required for optimal function [8,181,182]. Its dynamic responses to changes in neural activity, inflammation, and hypoxia further highlight its importance as an active regulator of brain homeostasis [6,138]. The functional roles of the BBB underscore its importance for maintaining CNS health and highlight the need for therapeutic strategies that can protect and support BBB integrity in neurodegenerative diseases [6,138].

### 2.6. BBB Dysfunction in Neurodegenerative Diseases

In neurodegenerative diseases, such as AD, PD, and multiple sclerosis, the BBB becomes increasingly compromised, disturbing the balance essential for brain health [12,183,184]. As tight junctions between endothelial cells weaken and transport mechanisms break down, blood-derived toxins, inflammatory molecules, and immune cells can infiltrate the brain [12,183,184] (Table 1). Such BBB disruption exacerbates neuroinflammation, accelerates neuronal damage, and promotes degeneration, establishing a self-perpetuating cycle that drives disease progression and facilitates cognitive and functional decline [12,183,184] (Table 1).

The integrity of the BBB is crucial for maintaining a stable internal environment in the CNS [11,19,185]. Its selective permeability ensures that only essential nutrients and ions reach the brain and waste products are efficiently removed [19,138]. However, in neurodegenerative diseases, this precise regulation is disrupted, allowing harmful substances to penetrate the BBB and disrupt neuronal function [12,183,184]. The breakdown of the BBB structure compromises the brain’s defense against external stressors and internal imbalances, resulting in increased vulnerability to inflammation and toxin accumulation [8,20,186].

The BBB is an intricately designed barrier that plays essential roles in CNS protection, brain homeostasis, and neuronal health [19,138,185]. Each component of the BBB functions together to regulate selective permeability, providing the brain with a controlled environment crucial for optimal neuronal function [167,187]. In neurodegenerative diseases, this balance is disrupted because of BBB dysfunction. Consequently, harmful agents can infiltrate and accelerate disease progression [12,183,184]. The preservation of BBB integrity is therefore vital for maintaining the protective environment of the brain and supporting overall neural health [8,11,186].

**Table 1:** Critical functions of the BBB and its disruption in neurodegenerative diseases.

	Functions	Contents	References
Function of the BBB	Protection of the CNS and selective permeability	The BBB protects the CNS by preventing harmful substances from entering it, ensuring neural health.	[6,9,11]
		Tight junctions and specialized transport systems block pathogens, toxins, and other molecules, reducing infection and inflammation risks.	[9,24,140]
		The BBB acts as a selective barrier against circulating pathogens such as bacteria and viruses, minimizing infections like meningitis and encephalitis.	[141,142]
		The BBB limits peripheral immune cell access, preventing excessive immune responses that could cause inflammation and neuronal damage.	[6,145]
		This immune regulation is vital to protect sensitive neural tissue from inflammation, preserving synaptic signaling and preventing neuronal death.	[146,147]
	Nutrient supply and waste removal	The BBB supports brain metabolism by transporting essential nutrients and removing metabolic waste.	[8,137,149]
		GLUT1 transports glucose for energy production and neurotransmitter synthesis, while LAT1 facilitates the uptake of amino acids for protein and neurotransmitter production.	[154,155,156,157,158,159]
		The BBB regulates ions such as K^+^ and Ca^2+^ to maintain ionic stability and support synaptic transmission.	[6,160]
		Efflux transporters, including P-gp, remove waste, xenobiotics, and toxins, preventing accumulation and oxidative stress.	[161,162,163]
	Adaptive regulation in response to neural activity	The BBB dynamically adjusts its permeability to meet the brain’s metabolic needs, neural activity, and environmental conditions.	[6,138,167]
		During heightened neural activity, the BBB increases blood flow and nutrient delivery to active brain regions through vasodilation and neurovascular coupling.	[170,171,172]
		In response to inflammation and oxidative stress, the BBB strengthens tight junctions and enhances efflux transporter activity to protect the CNS.	[57,173,174]
		Under hypoxia, the BBB upregulates glucose and metabolite transporters and boosts endothelial antioxidant defenses to counteract oxidative damage.	[172,179,180]
BBB disruption in neurodegenerative diseases	Weakened tight junctions and transport breakdown allow harmful substances to enter the brain	In neurodegenerative diseases, BBB disruption allows harmful substances to impair neuronal function.	[12,183,184]
	BBB disruption fuels neuroinflammation, neuronal damage, and degeneration in a vicious cycle	BBB disruption worsens neuroinflammation, accelerates damage, and drives cognitive decline in a self-perpetuating cycle.	[12,183,184]
		BBB disruption weakens brain defense, causing toxin buildup and increased vulnerability.	[8,20,186]

## 3. Mechanisms of BBB Disruption in Neurodegenerative Diseases

**Figure 2:**
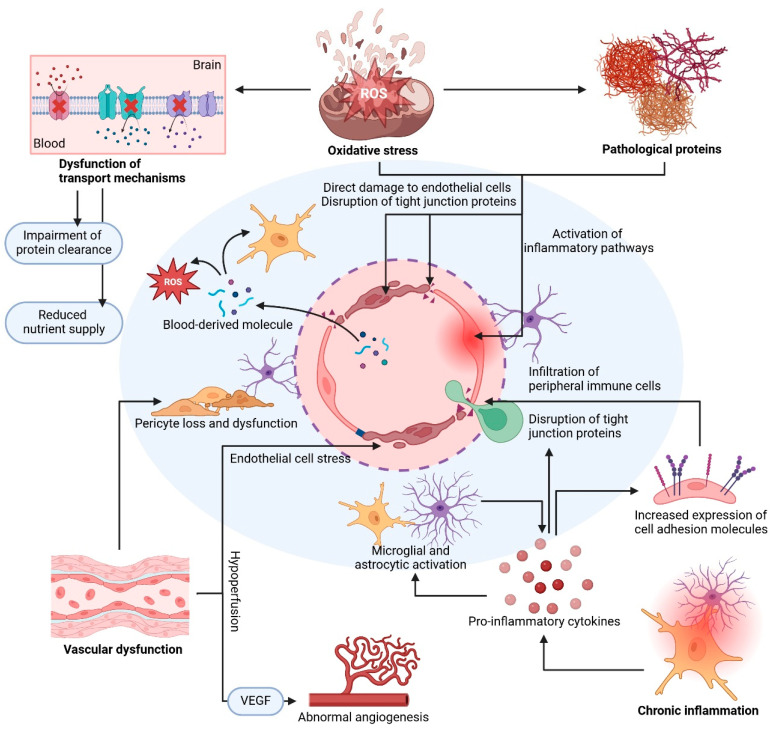
Mechanism of BBB disruption in the brain. Disruption of the BBB is a hallmark of several neurodegenerative diseases, including Alzheimer’s disease, Parkinson’s disease, and multiple sclerosis. Oxidative stress, chronic inflammation, pathological protein accumulation, and vascular dysfunction collectively weaken BBB integrity. Oxidative stress leads to increased production of reactive oxygen species (ROS) and reactive nitrogen species (RNS), which damage endothelial cells and tight junction proteins, thereby compromising the BBB and increasing its permeability. Chronic inflammation involves the release of proinflammatory cytokines by activated microglia and astrocytes, which weaken tight junctions and increase cell adhesion molecules. This facilitates immune cell infiltration into the CNS. Pathological proteins, such as Aβ, alpha-synuclein, and tau, accumulate and directly damage endothelial cells and tight junctions. They also activate inflammatory pathways that further destabilize the BBB. Vascular dysfunction, including hypoperfusion, vessel remodeling, and pericyte loss, limits oxygen and nutrient supply to the brain and weakens BBB stability. Lastly, impaired transport mechanisms disrupt nutrient supply and waste clearance, exacerbating neuronal damage. These factors collectively form a vicious cycle of BBB disruption and neurodegenerative disease progression. The figure was created using Biorender.com (Agreement number: ME27KUPC8L).

BBB disruption is a significant hallmark in several neurodegenerative diseases, including AD, PD, and multiple sclerosis, as well as acute disorders such as ischemic stroke [3,6,184,188]. Although each disease involves unique pathological changes, several shared mechanisms contribute to BBB breakdown [6,184,188] (Figure 2, Table 2). These include oxidative stress, chronic inflammation, protein accumulation, and vascular dysfunction, which collectively weaken the structural and functional integrity of the BBB [6,184,188] (Table 2).

### 3.1. Mechanisms of Oxidative Stress-Induced BBB Damage

Oxidative stress is a primary contributor to BBB breakdown in neurodegenerative diseases [189,190,191]. It involves an imbalance between the production of reactive oxygen species (ROS) and reactive nitrogen species (RNS) and the ability of the brain to detoxify these reactive molecules or repair the resulting cellular damage [192,193,194]. In neurodegenerative diseases, oxidative stress is significantly elevated. This results in cumulative damage to BBB components, particularly endothelial cells and tight junction proteins, which are crucial for maintaining BBB integrity [24,195,196]. ROS, including superoxide anions, hydroxyl radicals, and hydrogen peroxide, are byproducts of normal cellular metabolism [197,198]. However, in neurodegenerative diseases, ROS production is often significantly elevated because of mitochondrial dysfunction, NADPH oxidase activation, and inflammatory cell activity [189,199]. The production of RNS, particularly nitric oxide (NO) and peroxynitrite, is also elevated in the brain. This increase in production is often caused by the overactivation of inducible nitric oxide synthase (iNOS) in inflammatory and endothelial cells [200,201].

#### 3.1.1. Direct Damage to Endothelial Cells

BBB endothelial cells are highly susceptible to oxidative damage [195,196]. Excessive ROS and RNS can cause lipid peroxidation in the cell membranes, resulting in the loss of membrane integrity and functional alterations in endothelial cells [198,202,203]. Moreover, oxidative stress damages cellular organelles, particularly mitochondria, which are responsible for producing cellular energy [204,205,206]. Damaged mitochondria in turn release more ROS, creating a vicious cycle that amplifies oxidative stress within endothelial cells and weakens their barrier function [207,208,209].

#### 3.1.2. Degradation of Tight Junction Proteins

Tight junction proteins, such as claudin-5, occludin, and JAMs, are crucial for maintaining the tight seal between BBB endothelial cells [21,24,25]. Oxidative stress disrupts these proteins through several mechanisms [21,24,25]. In particular, ROS and RNS can compromise BBB integrity by modifying and degrading tight junction proteins [21,41,210]. Various processes, such as nitration, carbonylation, and oxidation, can alter the structure and function of these proteins, diminishing their ability to form effective seals between cells [211,212]. Oxidative stress can also activate proteases, such as MMPs, which degrade ECM components and tight junction proteins [213,214,215]. Upon overactivation, MMPs cleave proteins, such as claudin-5 and occludin, causing increased BBB permeability and enhanced vulnerability to inflammation and cellular damage [216,217,218].

#### 3.1.3. Activation of Inflammatory Pathways

Oxidative stress often triggers or enhances inflammatory responses, further contributing to BBB breakdown [196,219,220]. ROS and RNS can activate signaling pathways, such as the nuclear factor kappa-light-chain-enhancer of activated B cells (NF-κB) pathway, in endothelial cells, resulting in the production of proinflammatory cytokines (e.g., tumor necrosis factor-alpha [TNF-α] and interleukin-1 beta [IL-1β]) [221,222,223]. These cytokines disrupt the BBB by weakening tight junctions and promoting the infiltration of peripheral immune cells [24,224,225]. Moreover, oxidative stress can upregulate cell adhesion molecules (e.g., intercellular adhesion molecule-1 [ICAM-1] and vascular cell adhesion molecule-1 [VCAM-1]) on endothelial cells, facilitating the binding and migration of inflammatory cells across the BBB [226,227,228].

#### 3.1.4. Impact on Transport Mechanisms

Oxidative stress also affects the selective transport systems of the BBB, thereby disrupting the controlled passage of essential molecules [196,229]. In particular, ROS and RNS can damage carrier-mediated transport (CMT) and receptor-mediated transport (RMT) systems, lowering the efficiency of glucose and amino acid transport into the brain [198,230,231]. This deprivation of essential nutrients impairs neuronal metabolism and exacerbates neuronal vulnerability [232,233]. Additionally, oxidative stress reduces the function of efflux transporters, such as P-gp, leading to the accumulation of neurotoxic substances within the CNS [177,229,234].

### 3.2. Mechanisms of BBB Disruption Due to Chronic Inflammation

Chronic inflammation is a hallmark of many neurodegenerative diseases. It plays a crucial role in weakening the BBB [6,184,235]. In neurodegenerative diseases, persistent inflammatory responses disrupt BBB integrity, facilitating disease progression [4,5,188]. Activated microglia (resident immune cells in the brain) and astrocytes release proinflammatory cytokines, such as TNF-α, IL-1β, and IL-6. These cytokines affect the structural and functional stability of the BBB, primarily by disrupting tight junctions and altering endothelial cell function [6,236,237].

#### 3.2.1. Release of Proinflammatory Cytokines

Microglia and astrocytes are persistently activated in response to injury, protein accumulation, or environmental stress within the brain [238,239,240]. These activated glial cells release high levels of proinflammatory cytokines, including TNF-α, IL-1β, and IL-6 [239,241,242]. These cytokines bind to receptors on endothelial cells and trigger intracellular signaling cascades that weaken the BBB [167,224].

Proinflammatory cytokines play a significant role in compromising BBB integrity [225,243,244]. TNF-α binds to receptors on endothelial cells and activates signaling pathways, including the NF-κB pathway, which upregulates cell adhesion molecules, such as ICAM-1 and VCAM-1 [245,246,247]. This upregulation facilitates immune cell adhesion and migration across the BBB, resulting in heightened permeability [167,248,249]. Meanwhile, IL-1β and IL-6 contribute to inflammatory responses by upregulating MMPs and disrupting the expression of tight junction proteins, further weakening the BBB and increasing its susceptibility to inflammatory damage [12,21,244].

#### 3.2.2. Disruption of Tight Junction Proteins Due to Inflammation

Chronic inflammation disrupts tight junction proteins that hold endothelial cells together and prevent paracellular transport [250,251,252]. Proinflammatory cytokines alter the expression and function of tight junction proteins, such as claudin-5, occludin, and ZO-1 [250,253,254]. Various cytokines, such as TNF-α and IL-1β, weaken the BBB by downregulating tight junction proteins at both transcriptional and translational levels, thereby reducing the tightness of the BBB and allowing toxic substances to pass more easily [21,243,255]. Additionally, inflammatory signaling can cause the phosphorylation of tight junction proteins. Consequently, they are redistributed away from cell–cell junctions [251,256]. This redistribution further compromises the BBB structure, increasing paracellular permeability and making the barrier more susceptible to infiltration and potential damage from external substances [251,256].

#### 3.2.3. Activation of MMPs

Chronic inflammation often leads to the activation of MMPs, which degrade the ECM and basement membrane components [257,258]. Activated MMPs, particularly MMP-9 and MMP-2, break down collagen and laminin in the basement membrane, in addition to breaking down tight junction proteins [132,259,260]. This degradation not only weakens the physical structure of the BBB but also facilitates the infiltration of immune cells and inflammatory molecules into the CNS, further exacerbating neuroinflammation [261,262].

#### 3.2.4. Increased Expression of Cell Adhesion Molecules

Inflammatory cytokines stimulate the expression of cell adhesion molecules on endothelial cells, such as ICAM-1 and VCAM-1 [263,264,265]. These cell adhesion molecules allow peripheral immune cells to bind to endothelial cells, enabling their passage through the BBB into brain tissue [225,226,266]. This infiltration of immune cells, including T cells and macrophages, amplifies the inflammatory response within the CNS, creating a self-sustaining cycle of BBB damage and neuroinflammation [145,267,268].

#### 3.2.5. Microglial and Astrocytic Activation

The chronic activation of microglia and astrocytes creates a sustained inflammatory environment that can damage the BBB over time [236,238,269]. Microglia release additional ROS, RNS, and cytokines, further impairing endothelial cell function and degrading tight junction proteins [12,270,271]. Although astrocytes are typically beneficial to the BBB, they can contribute to inflammation by releasing IL-6 and other cytokines when chronically activated [272,273,274]. Such persistent glial activation amplifies the disruption of BBB integrity and perpetuates an environment of neuroinflammation [12,275].

#### 3.2.6. Infiltration of Peripheral Immune Cells

Increased BBB permeability due to chronic inflammation allows peripheral immune cells, such as monocytes, T cells, and neutrophils, to infiltrate the CNS [6,276,277]. Within the brain, these cells release additional proinflammatory molecules, further promoting neuroinflammation and worsening BBB damage [12,278,279]. The resulting cycle of immune cell infiltration and inflammation creates a feed-forward loop, wherein inflammation continuously drives BBB disruption, in turn accelerating neurodegenerative processes [6,119,280].

### 3.3. Mechanisms of BBB Disruption by Pathological Proteins

The accumulation of abnormal proteins is a hallmark of neurodegenerative diseases and plays a significant role in disrupting BBB integrity [128,184,281]. In AD, PD, and various tauopathies, specific pathological proteins, such as amyloid-beta (Aβ), alpha-synuclein, and tau, respectively, accumulate in the brain [282,283,284]. These proteins exhibit toxic effects on the BBB by directly damaging endothelial cells, disrupting tight junctions, and triggering inflammatory responses. All these effects collectively increase BBB permeability and worsen disease progression [128,281,285].

#### 3.3.1. Direct Damage to Endothelial Cells and Tight Junction Proteins

Aβ, alpha-synuclein, and tau proteins accumulate near and within BBB endothelial cells, causing cellular stress and structural damage [5,128,285]. For instance, Aβ can integrate into endothelial cell membranes, altering membrane integrity and triggering cellular stress pathways that weaken the BBB [286,287]. Similarly, excessive accumulation of alpha-synuclein and tau can disrupt cellular function, causing cellular stress and compromising the protective barrier function of endothelial cells [128,281,285].

Pathological proteins weaken tight junctions that seal endothelial cells, directly increasing BBB permeability [21,288,289]. These proteins can interfere with the expression and stability of key tight junction proteins, such as claudin-5 and occludin [184,281,290]. Aβ, for instance, can downregulate the expression of tight junction proteins by activating inflammatory and oxidative stress pathways, which phosphorylate and degrade tight junction proteins, thereby weakening the tight seal of the BBB [165,291,292].

#### 3.3.2. Activation of Inflammatory Response by Pathological Proteins

The accumulation of pathological proteins activates microglia and astrocytes, which release proinflammatory cytokines [238,239,269]. These cytokines disrupt BBB structure by targeting endothelial cells and promoting the activity of MMPs, which can degrade ECM components and further destabilize the BBB [11,224,261]. For instance, the accumulation of Aβ activates microglia, resulting in sustained inflammation that damages BBB integrity and increases the vulnerability of the brain to peripheral toxins and immune cells [128,271,293].

#### 3.3.3. Impairment of Protein Clearance Mechanisms

The accumulation of pathological proteins is exacerbated by an impaired ability of the BBB to clear these toxins [128,184,294]. Normally, the BBB contains transporters and efflux mechanisms (e.g., low-density lipoprotein receptor-related protein 1 [LRP1]) to clear proteins like Aβ from the brain [295,296,297]. However, chronic accumulation of these proteins disrupts these clearance pathways, resulting in further accumulation of toxic proteins in the brain and exacerbating BBB permeability [295,296,297]. In AD, for instance, LRP1 is downregulated in response to high levels of Aβ, slowing its clearance and leading to increased deposition in the brain and around blood vessels [295,296,298].

#### 3.3.4. Oxidative Stress Induced by Pathological Proteins

Pathological proteins also contribute to oxidative stress, further damaging the BBB [128]. Aβ, alpha-synuclein, and tau can increase the production of ROS within endothelial cells and nearby glial cells [299,300,301]. Such oxidative stress damages cellular structures, including tight junctions and cell membranes, promoting BBB leakage [128]. ROS can further activate inflammatory pathways, creating a cycle of inflammation, oxidative stress, and BBB damage [3,270,302].

### 3.4. Mechanisms of BBB Disruption Due to Vascular Dysfunction

Vascular dysfunction is a prominent feature of neurodegenerative diseases. It significantly enhances BBB disruption [6,135,183]. In neurodegenerative diseases, abnormalities in blood flow, structural remodeling of blood vessels, and direct damage to the vascular system impact BBB integrity and facilitate disease progression [4,6,184]. These vascular changes compromise endothelial cell function, reduce the supply of oxygen and nutrients to the brain, and weaken tight junctions, leading to increased BBB permeability and heightened neuronal damage [3].

#### 3.4.1. Reduced Blood Flow to the Brain (Hypoperfusion)

Hypoperfusion is commonly observed in neurodegenerative diseases [303,304,305]. It deprives brain cells, including endothelial cells, of essential oxygen and nutrients, thereby creating a stressful environment that compromises BBB stability [6,305,306].

Inadequate oxygen and glucose supply leads to endothelial stress and dysfunction, causing mitochondrial impairment, increased ROS production, and a breakdown of tight junction integrity [307,308,309]. This stressful environment increases BBB permeability and heightens its vulnerability to damage [3,41,306]. Moreover, hypoperfusion creates hypoxic conditions within the brain. Low oxygen levels further impair cellular function and weaken tight junctions [24,305,310]. This hypoxic injury renders the BBB more susceptible to external stressors, allowing neurotoxic substances to penetrate the brain and potentially cause additional damage [19,311,312].

#### 3.4.2. Vascular Remodeling

Blood vessels often undergo structural remodeling to adapt to the reduced blood supply resulting from chronic hypoperfusion or hypoxia [313]. While these changes are initially compensatory, they can ultimately destabilize the BBB [314,315,316].

In neurodegenerative diseases, structural changes in the BBB compromise its function [6,127,184]. The basement membrane surrounding blood vessels can thicken abnormally, disrupting the NVU and weakening interactions between endothelial cells, pericytes, and astrocytes, thereby weakening the protective role of the BBB [20,104,317]. Additionally, hypoxia-induced angiogenesis, primarily driven by VEGF, leads to the formation of new blood vessels [318,319]. However, these newly formed vessels are often immature and lack proper tight junctions, making them leaky and facilitating the entry of unwanted substances into the brain [21,148,320]. This increased permeability further disrupts the CNS environment and weakens BBB integrity [8,11,138].

#### 3.4.3. VEGF and Tight Junction Disruption

VEGF is upregulated in hypoxic and neuroinflammatory conditions, commonly noted in neurodegenerative diseases [321,322,323]. While VEGF promotes angiogenesis, it also disrupts BBB integrity [324,325,326].

Moreover, VEGF facilitates the breakdown of the BBB by downregulating tight junction proteins, thereby increasing paracellular permeability and weakening BBB integrity [24,25,327]. As the expression of tight junction proteins decreases, endothelial cells become less cohesive, creating gaps that allow blood-derived toxins and inflammatory molecules to infiltrate the brain [21,24,328]. Additionally, VEGF signaling through receptors on endothelial cells promotes vascular permeability by inducing the disassembly of tight junction proteins and causing the retraction of endothelial cells [329,330]. This increased permeability allows harmful substances to cross the BBB, further exacerbating neuroinflammation and contributing to CNS dysfunction [6,12,175].

#### 3.4.4. Pericyte Loss and Dysfunction

Pericytes play a crucial role in maintaining vascular stability and BBB integrity [34,52,331]. In neurodegenerative diseases, pericyte loss and dysfunction can contribute to vascular abnormalities [44,332,333].

Reduced pericyte coverage weakens the BBB by depriving endothelial cells of the necessary structural support, thereby compromising tight junction integrity and increasing permeability [34,60,334]. This decrease in pericyte support, observed in neurodegenerative diseases, is associated with an increase in the permeability and instability of blood vessels [335,336]. Pericytes also play a crucial role in regulating capillary blood flow in response to neural activity. When pericytes are dysfunctional, they fail to adjust blood flow adequately, resulting in impaired circulation and hypoxic conditions [68,337,338]. These hypoxic conditions further weaken BBB integrity, making the brain more vulnerable to external stressors and potential damage [339,340,341].

### 3.5. Dysfunction of Transport Mechanisms

In neurodegenerative diseases, BBB disruption is frequently accompanied by dysfunction of the transport systems responsible for the controlled exchange of nutrients, ions, and waste products between the bloodstream and the brain [5,6,184]. Two primary transport mechanisms, namely CMT and RMT, typically regulate the selective passage of essential molecules, while efflux transporters actively remove neurotoxic substances from the brain [342,343,344]. When these systems are compromised, as commonly seen in neurodegenerative diseases, the supply of essential nutrients to the brain is reduced and the clearance of harmful waste products is impaired. Both these effects accelerate neuronal damage and exacerbate disease progression [4,6,119].

#### 3.5.1. CMT Impairment

CMT systems selectively move small molecules, such as glucose, amino acids, and ions, across the BBB [154,156,343]. These transporters play a crucial role in providing neurons with the resources essential for energy and neurotransmitter synthesis [154,156,159].

In neurodegenerative diseases, dysfunctional nutrient transport across the BBB significantly impacts brain health [4,6]. The expression of GLUT1, responsible for delivering glucose, is often downregulated in these conditions, resulting in impaired glucose transport [345,346,347]. The resultant energy shortage makes neurons more vulnerable to stress and damage. For instance, in AD, decreased GLUT1 levels correlate with cognitive decline caused by energy deficits that hinder neuronal function and synaptic transmission [345,346,348]. Additionally, amino acid transport is compromised by the decreased function of carrier proteins, such as LAT1, which transports essential amino acids, such as tryptophan and tyrosine [159,349,350]. These amino acids are precursors for serotonin and dopamine, respectively, which are neurotransmitters crucial for mood and cognition [351,352]. Limited availability of these precursors disrupts neurotransmitter synthesis, impairing neural signaling and potentially contributing to the mood and cognitive disturbances associated with neurodegenerative diseases [353,354,355].

#### 3.5.2. RMT Impairment

Larger molecules, such as hormones, growth factors, and certain proteins, rely on RMT to cross the BBB [11,153]. In neurodegenerative diseases, RMT pathways become dysfunctional, affecting the delivery of key molecules into the brain [6,356].

Insulin and other growth factors, crucial for brain function, rely on specific transport mechanisms at the BBB that can be compromised in neurodegenerative diseases [357,358,359]. Insulin, which crosses the BBB through insulin receptors on endothelial cells, is essential for neuronal glucose metabolism and growth signaling [360,361,362]. However, in AD, insulin receptors often function poorly, causing brain insulin resistance [363,364,365]. This reduced insulin transport results in energy deficits and impaired neural plasticity, potentially accelerating disease progression [364,366]. Similarly, transferrin receptors are responsible for transporting iron, which is vital for neuronal function [367,368,369]. In PD and other neurodegenerative disorders, disrupted regulation of iron transport can lead to iron accumulation in certain brain regions. This can result in oxidative stress and neurotoxicity, further exacerbating neuronal damage [370,371,372].

#### 3.5.3. Efflux Transporter Dysfunction

Efflux transporters, such as P-gp, are crucial for clearing neurotoxic substances from the brain [370,371,372]. These ATP-dependent transporters actively pump out toxins, xenobiotics, and metabolic byproducts, helping maintain the internal environment of the brain [163,373,374]. However, in neurodegenerative diseases, the function of efflux transporters is often impaired [6,182,375].

P-gp and other efflux transporters are essential for clearing neurotoxic substances from the brain; however, their downregulation in neurodegenerative diseases leads to harmful accumulations [177,182]. P-gp expels Aβ from the brain. However, decreased expression and activity of P-gp in AD can contribute to the accumulation of Aβ, exacerbating plaque formation, neuroinflammation, and neuronal loss [376,377,378]. Similarly, dysfunctional efflux transporters enable the accumulation of harmful metabolic byproducts, environmental toxins, and drugs, increasing oxidative stress and damaging neurons [6,182,229]. In PD, impaired clearance of neurotoxins intensifies dopaminergic neuron loss, worsening motor dysfunction and advancing disease progression [379,380,381].

#### 3.5.4. Disrupted Ion Transport

BBB transport mechanisms also regulate ion balance, ensuring that the ionic environment of the brain remains optimal for neuronal activity [11,382,383]. In neurodegenerative diseases, the regulation of ions becomes impaired [4,6,119]. The regulation of calcium ions, K^+^, and sodium ions is crucial for maintaining neuronal signaling and excitability. However, dysregulation of these ions can lead to severe neuronal stress and damage in neurodegenerative diseases [384,385]. Ion channels and transporters tightly regulate calcium and potassium concentrations in the brain; however, dysfunction of these pathways can result in abnormal concentrations of ions, causing excitotoxicity or neuronal hyperactivity that damages neurons over time [102,386,387]. Similarly, sodium–potassium ATPase, which maintains the sodium and potassium balance across the BBB, can become impaired in neurodegenerative diseases, leading to imbalances in neuronal resting potentials and the disruption of electrical signaling [388]. This dysregulation places additional stress on neurons, as seen in multiple sclerosis, wherein ion imbalance is particularly prevalent, further compromising neural health [389,390].

**Table 2:** Mechanisms of BBB disruption in neurodegenerative diseases.

Title	Subtitle	Contents	References
Mechanisms of oxidative stress-induced BBB damage	Direct damage to endothelial cells	Oxidative stress induces lipid peroxidation, compromising membrane integrity and endothelial cell function.	[198,202,203]
		Damaged cellular organelles, especially mitochondria, further amplify oxidative stress by releasing more ROS.	[204,205,206]
		This vicious cycle weakens the barrier function of endothelial cells, exacerbating BBB dysfunction.	[207,208,209]
	Degradation of tight junction proteins	Oxidative stress disrupts tight junction proteins through ROS- and RNS-induced modifications, such as nitration, carbonylation, and oxidation.	[21,41,210]
		Oxidative stress activates proteases such as MMPs, which degrade ECM components and tight junction proteins.	[213,214,215]
		Overactive MMPs cleave claudin-5 and occludin, increasing BBB permeability and vulnerability to inflammation and damage.	[216,217,218]
	Activation of inflammatory pathways	ROS and RNS activate the NF-κB pathway, leading to the release of proinflammatory cytokines, including TNF-α and IL-1β.	[221,222,223]
		Proinflammatory cytokines weaken tight junctions and promote immune cell infiltration, disrupting the BBB.	[24,224,225]
		Oxidative stress upregulates adhesion molecules (ICAM-1 and VCAM-1), enhancing inflammatory cell migration across the BBB.	[226,227,228]
	Impact on transport mechanisms	ROS and RNS damage CMT and RMT, reducing glucose and amino acid delivery to the brain.	[198,230,231]
		Nutrient deprivation impairs neuronal metabolism, increasing neuronal vulnerability.	[232,233]
		Oxidative stress diminishes efflux transporter function (e.g., P-gp), causing neurotoxic substance accumulation in the CNS.	[177,229,234]
Mechanisms of BBB disruption due to chronic inflammation	Release of proinflammatory cytokines	Microglia and astrocytes are activated by brain injury, protein accumulation, or stress, releasing proinflammatory cytokines such as TNF-α, IL-1β, and IL-6.	[239,241,242]
		TNF-α activates the NF-κB pathway, upregulating adhesion molecules (ICAM-1 and VCAM-1) and promoting immune cell migration across the BBB.	[245,246,247]
		IL-1β and IL-6 increase inflammation by upregulating MMPs and disrupting tight junction proteins, further compromising BBB integrity.	[12,21,244]
	Disruption of tight junction proteins due to inflammation	Proinflammatory cytokines such as TNF-α and IL-1β downregulate tight junction proteins (claudin-5, occludin, and ZO-1) at transcriptional and translational levels.	[250,253,254]
		Reduced tight junction protein expression weakens the BBB, allowing easier passage of toxic substances.	[21,243,255]
		Inflammatory signaling phosphorylates tight junction proteins, causing their redistribution away from cell junctions.	[251,256]
	Activation of MMPs	Chronic inflammation activates MMPs, especially MMP-9 and MMP-2, which degrade ECM and basement membrane components.	[132,259,260]
		MMPs break down collagen, laminin, and tight junction proteins, weakening the BBB structure.	[261,262]
	Increased expression of cell adhesion molecules	Inflammatory cytokines upregulate cell adhesion molecules (ICAM-1 and VCAM-1) on endothelial cells.	[263,264,265]
		These adhesion molecules enable peripheral immune cells to bind and cross the BBB into brain tissue.	[225,226,266]
	Microglial and astrocytic activation	Microglia release ROS, RNS, and cytokines, impairing endothelial function and degrading tight junction proteins.	[12,270,271]
		Chronically activated astrocytes release IL-6 and other cytokines, contributing to inflammation.	[272,273,274]
		Persistent glial activation amplifies BBB disruption and perpetuates neuroinflammation.	[12,275]
	Infiltration of peripheral immune cells	Chronic inflammation increases BBB permeability, allowing immune cells such as monocytes, T cells, and neutrophils to infiltrate the CNS.	[6,276,277]
		Infiltrating immune cells release proinflammatory molecules, worsening neuroinflammation and BBB damage.	[12,278,279]
		This creates a feed-forward loop where inflammation perpetuates BBB disruption, accelerating neurodegenerative processes.	[6,119,280]
Mechanisms of BBB disruption by pathological proteins	Direct damage to endothelial cells and tight junction proteins	Aβ integrates into endothelial membranes, altering integrity and activating stress pathways that weaken the BBB.	[286,287]
		Alpha-synuclein and tau disrupt endothelial cell function, compromising the BBB’s protective layer.	[128,281,285]
		Pathological proteins weaken tight junctions by interfering with the expression and stability of proteins such as claudin-5 and occludin.	[184,281,290]
		Aβ downregulates tight junction proteins through inflammatory and oxidative stress pathways, increasing BBB permeability.	[165,291,292]
	Activation of inflammatory response by pathological proteins	Pathological protein accumulation activates microglia and astrocytes, leading to the release of proinflammatory cytokines.	[238,239,269]
		These cytokines disrupt BBB structure by targeting endothelial cells and activating MMPs.	[11,224,261]
	Impairment of protein clearance mechanisms	The BBB normally uses transporters (e.g., LRP1) to remove proteins such as Aβ from the brain.	[295,296,297]
		Chronic protein accumulation disrupts these clearance pathways, increasing toxic protein buildup and BBB permeability.	[295,296,297]
		In AD, LRP1 is downregulated in response to high Aβ levels, slowing its clearance and promoting further deposition in the brain and blood vessels.	[295,296,298]
	Oxidative stress induced by pathological proteins	Pathological proteins increase ROS production in endothelial and glial cells.	[299,300,301]
Mechanisms of BBB disruption due to vascular dysfunction	Reduced blood flow to the brain (hypoperfusion)	Hypoperfusion in neurodegenerative diseases deprives brain cells of oxygen and nutrients, compromising BBB stability.	[6,303,304,305,306]
		Oxygen and glucose deprivation cause endothelial stress, mitochondrial dysfunction, and increased ROS production.	[307,308,309]
		Hypoperfusion-induced hypoxia weakens tight junctions and impairs cellular function.	[24,305,310]
	Vascular remodeling	In neurodegenerative diseases, abnormal thickening of the basement membrane disrupts the NVU and weakens BBB function.	[20,104,317]
		Hypoxia-induced angiogenesis driven by VEGF creates immature, leaky blood vessels lacking proper tight junctions.	[318,319]
	VEGF and tight junction disruption	While VEGF promotes angiogenesis, it disrupts BBB integrity by downregulating tight junction proteins.	[24,25,327]
		VEGF signaling causes tight junction disassembly and endothelial cell retraction, enhancing vascular permeability.	[329,330]
	Pericyte loss and dysfunction	Reduced pericyte coverage weakens tight junctions, increasing BBB permeability and instability.	[34,60,334]
		Dysfunctional pericytes fail to regulate capillary blood flow, leading to impaired circulation and hypoxic conditions.	[68,337,338]
Dysfunction of transport mechanisms	CMT impairment	CMT systems transport small molecules such as glucose, amino acids, and ions across the BBB, essential for neuronal energy and neurotransmitter synthesis.	[154,156,159]
		GLUT1 downregulation impairs glucose transport, causing energy deficits that increase neuronal vulnerability and hinder synaptic transmission.	[345,346,348]
		Decreased LAT1 function limits amino acid transport, reducing precursors for neurotransmitters, including serotonin and dopamine.	[159,349,350,351,352]
	RMT impairment	Larger molecules such as hormones, growth factors, and proteins cross the BBB via RMT. In neurodegenerative diseases, RMT dysfunction disrupts the delivery of essential molecules to the brain.	[6,11,153,356]
		Insulin, crucial for glucose metabolism and growth signaling, crosses the BBB through insulin receptors, but brain insulin resistance in AD impairs this process.	[360,361,362,363,364,365]
		Transferrin receptors regulate iron transport, but their dysfunction in PD and other disorders causes iron accumulation in the brain.	[367,368,369,370,371,372]
	Efflux transporter dysfunction	P-gp clears Aβ, but its downregulation in AD promotes Aβ buildup, exacerbating plaque formation, neuroinflammation, and neuronal loss.	[177,182,376,377,378]
		Dysfunctional transporters allow metabolic byproducts, toxins, and drugs to accumulate, increasing oxidative stress and neuronal damage.	[6,182,229]
	Disrupted ion transport	Ca^2+^, K^+^, and Na^+^ ion dysregulation leads to excitotoxicity and neuronal hyperactivity, damaging neurons over time.	[384,385]
		Dysfunctional ion channels and transporters disrupt Ca^2+^ and K^+^ concentrations, exacerbating neuronal damage.	[102,386,387]
		Impaired Na^+^–K^+^ ATPase disrupts ion balance, altering neuronal resting potentials and electrical signaling.	[388]

## 4. Consequences of BBB Disruption

When the BBB is compromised, it sets off a cascade of pathological processes that accelerate neurodegeneration [6,119,183]. This weakened BBB allows immune cells, blood-derived molecules, and toxic substances to infiltrate the brain, triggering neuroinflammation, neurotoxicity, and abnormal protein aggregation [4,184]. These processes interact in a self-perpetuating cycle that worsens neuronal damage and accelerates disease progression [391,392,393].

### 4.1. Neuroinflammation

BBB disruption allows immune cells, such as macrophages, T cells, and B cells, to infiltrate the brain parenchyma [394,395,396]. Under normal conditions, the BBB acts as a selective barrier that prevents these peripheral immune cells from entering the CNS [395,397]. However, once the BBB is compromised, its increased permeability enables these cells to enter the brain, triggering a potent neuroinflammatory response [398,399].

The entry of peripheral immune cells into the brain triggers an inflammatory cascade that disrupts BBB integrity and accelerates neurodegeneration [400,401]. Immune cell infiltration activates resident immune cells, such as microglia and astrocytes, which release proinflammatory cytokines, thereby amplifying inflammation and causing further neuronal and glial cell damage [82,239,402]. Microglia enter a proinflammatory state, releasing additional cytokines, ROS, and RNS. Furthermore, astrocytes, which usually support BBB function, contribute to the inflammatory environment by releasing additional cytokines and chemokines [403,404,405]. This inflammatory response damages endothelial cells and disrupts tight junctions, increasing BBB permeability and allowing even more immune cells to infiltrate the brain. Consequently, a feedback loop of inflammation and barrier breakdown is created [21,24,397]. This cycle of chronic neuroinflammation accelerates neurodegeneration by directly harming neurons, increasing oxidative stress, and interfering with neuronal signaling [406,407].

### 4.2. Neurotoxicity from Blood-Derived Molecules

In a healthy brain, the BBB restricts the entry of blood-derived molecules [6,8,11,14,408]. However, when the BBB is compromised, these substances leak into the brain parenchyma, causing neuronal damage and exacerbating neurodegeneration [4,14,294,408,409].

When the BBB is compromised, various blood proteins, such as prothrombin, thrombin, prothrombin kringle-2 (pKr-2), fibrinogen, and albumin, also infiltrate the brain, initiating a cascade of neuroinflammatory and neurodegenerative processes [14,408]. Moreover, thrombin stimulates microglia and astrocytes to release inflammatory mediators, impairing synaptic plasticity, neuronal signaling, and overall neuronal health [14,410,411]. Prolonged exposure to thrombin and other blood proteins exacerbates neuronal dysfunction and can cause neuronal death [14,410,411]. pKr-2, a fragment of prothrombin, is indirectly neurotoxic. It specifically activates microglia and induces a proinflammatory response that results in elevated cytokine production and oxidative stress, further damaging neurons [14,408,412]. Fibrinogen is another clotting protein that is particularly detrimental to the CNS [413]. Once it crosses the compromised BBB, it activates microglia to release additional cytokines and ROS, thereby intensifying neuroinflammation and causing BBB disruption [14,413]. In AD, fibrinogen also binds to Aβ, promoting its aggregation into plaques and accelerating disease progression [414,415]. Albumin is typically restricted from entering the brain. However, upon BBB breakdown, it further contributes to neuroinflammation [12,294,398]. By disrupting neural ion balance, it causes excitotoxicity and activates astrocytes, which release inflammatory mediators that weaken the BBB and amplify inflammation [416,417,418]. This cycle of protein infiltration, neuroinflammation, and oxidative stress establishes a feedback loop that progressively worsens neuronal function, potentially causing irreversible neurodegeneration [12,14,294].

### 4.3. Abnormal Protein Aggregation

BBB breakdown also facilitates the accumulation and aggregation of pathological proteins, such as Aβ in AD and alpha-synuclein in PD [184,419,420]. These proteins disrupt neuronal function and form aggregates, such as Aβ plaques in AD and Lewy bodies in PD [421,422,423]. The presence of these aggregates initiates a series of damaging processes that accelerate neurodegeneration [424,425]. The compromised BBB allows the accumulation of pathological proteins within the brain. These proteins can aggregate because of reduced clearance [127,128,184]. Upon accumulation, Aβ and alpha-synuclein are particularly prone to aggregation, forming insoluble deposits that are resistant to normal degradation pathways [426,427,428].

Protein aggregates in neurodegenerative diseases interfere with neuronal function and create a toxic environment that accelerates neurodegeneration [424,429,430]. These aggregates disrupt essential cellular processes, such as synaptic signaling, cellular transport, and mitochondrial function [428,431,432]. For instance, Aβ plaques disrupt calcium signaling, which is vital for neurotransmission, while alpha-synuclein aggregates impair vesicle trafficking and dopamine release, thereby affecting motor function in PD [433,434,435]. These aggregates also activate microglia and astrocytes, which release proinflammatory cytokines and ROS, establishing a chronic state of neuroinflammation and oxidative stress [238,436,437]. This toxic environment further damages neurons and promotes additional protein aggregation, creating a self-perpetuating cycle of degeneration [238,407,437]. The formed aggregates are resistant to degradation and persist within the brain, disrupting neuronal networks and contributing to neuronal loss over time [438,439,440]. The presence of these persistent structures is linked to the progressive cognitive and motor deficits characteristic of neurodegenerative diseases [135,441,442].

## 5. Therapeutic Approaches to Preserve BBB Integrity

**Figure 3:**
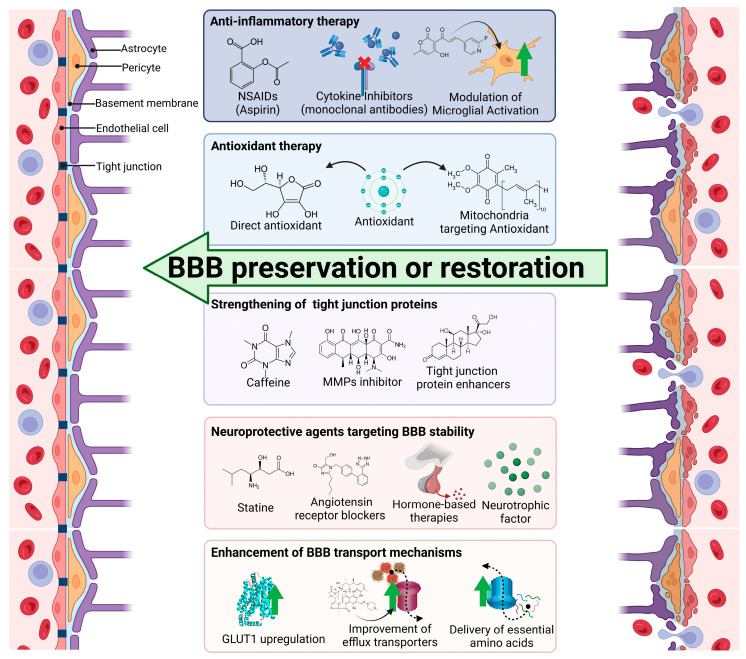
Therapeutic approach to preserve the BBB. In neurodegenerative diseases, BBB damage accelerates neurodegeneration. Hence, strategies to preserve or restore BBB integrity are crucial for delaying disease progression and protecting brain function. Key approaches include the use of anti-inflammatory and antioxidant therapies, strengthening of tight junction proteins, enhancement of BBB transport mechanisms, and use of neuroprotective agents. Anti-inflammatory therapies reduce BBB damage by inhibiting inflammatory cytokine and immune cell infiltration, while antioxidant therapies protect BBB components from oxidative stress caused by ROS. The upregulation of tight junction proteins decreases BBB permeability. Moreover, improved transport mechanisms ensure the delivery of essential nutrients to the brain and the clearance of toxic substances from the brain, while neuroprotective agents directly enhance BBB stability. Together, these strategies help maintain BBB integrity, creating a stable environment in the brain and protecting neural functions. Thus, multifaceted approaches to protect and restore the BBB are vital therapeutic targets for preserving brain health in neurodegenerative diseases. The figure was created using Biorender.com (Agreement number: AH27QDI3FF).

The BBB is essential for maintaining a stable environment in the CNS. It regulates the exchange of substances and protects neurons from toxins, inflammation, and other stressors [6,9,11]. In neurodegenerative diseases, BBB disruption accelerates neurodegeneration. Hence, therapeutic approaches to preserve or restore BBB integrity are crucial [6,15,119]. These strategies include the use of anti-inflammatory and antioxidant therapies; modulation of tight junctions; enhancement of transport mechanisms; and use of neuroprotective agents, such as caffeine, which can support BBB stability [21,231,443] (Figure 3).

### 5.1. Anti-Inflammatory Therapies

Chronic inflammation is a major contributor to BBB disruption in neurodegenerative diseases [6,444]. Anti-inflammatory therapies aim to reduce the damaging effects of proinflammatory cytokines and immune cell infiltration, thereby stabilizing the BBB [243,445,446].

Therapeutic strategies aimed at reducing neuroinflammation and protecting the BBB are being explored to combat BBB damage in neurodegenerative diseases [13,184,447]. For example, nonsteroidal anti-inflammatory drugs (NSAIDs) inhibit various enzymes, such as COX-2, which lowers the production of proinflammatory cytokines that can weaken BBB integrity [445,446,448]. Certain NSAIDs may exhibit neuroprotective effects in AD, potentially slowing BBB damage by reducing inflammatory signaling [401,449]. Additionally, targeting specific cytokines, such as TNF-α, IL-1β, and IL-6, with cytokine inhibitors, including monoclonal antibodies and other biologics, may directly reduce BBB permeability and neuroinflammation [175,444,450]. Modulating microglial activation is promising, as lowering the activation of these resident immune cells can help minimize neuroinflammation and prevent further BBB damage [271,279,451]. Different compounds, such as PPAR-γ agonists and other anti-inflammatory agents, are currently under investigation to modulate microglial activity in order to reduce chronic inflammation and protect the BBB from ongoing disruption [452,453,454]. Moreover, emerging therapeutic approaches are focusing on more precise targets to combat inflammation-induced BBB disruption. For instance, Janus kinase inhibitors, such as tofacitinib, have shown potential in blocking key inflammatory pathways mediated by cytokines, thereby reducing endothelial activation and stabilizing the BBB [455,456]. Furthermore, NF-κB inhibitors are being developed to downregulate pro-inflammatory gene expression, which is critical in mitigating BBB damage [457]. Another promising strategy involves the use of MMP inhibitors, such as doxycycline, to prevent the degradation of tight junction proteins and basement membrane components, thereby preserving the structural integrity of the BBB [132,458,459].

### 5.2. Antioxidant Therapies

Oxidative stress significantly contributes to BBB breakdown by damaging endothelial cells and tight junction proteins [196,460]. Antioxidant therapies aim to neutralize ROS and RNS, protecting BBB components from oxidative damage [300,461,462].

Various antioxidant strategies are being explored to protect BBB integrity by reducing oxidative stress in neurodegenerative diseases [463,464,465]. Direct antioxidants, such as vitamin E, vitamin C, and N-acetylcysteine (NAC), can neutralize ROS in the brain, potentially preventing oxidative damage to BBB endothelial cells and preserving tight junctions [466,467,468]. Another approach is targeting the source of ROS, namely mitochondrial dysfunction, in endothelial cells. Antioxidants targeting the mitochondria, such as MitoQ and coenzyme Q10, are being studied for their potential to reduce ROS production within mitochondria and support BBB health [469,470]. Moreover, polyphenols, such as resveratrol, curcumin, and flavonoids, are plant-based compounds with antioxidant and anti-inflammatory properties [471,472,473]. They can cross the BBB and may further support BBB integrity by reducing oxidative stress and inflammation in the CNS [460,474,475]. Emerging antioxidant therapies are leveraging innovative strategies to combat oxidative stress and protect BBB integrity in neurodegenerative diseases. Enzyme-based antioxidants, such as superoxide dismutase mimetics and catalase formulations, directly neutralize ROS, while nanoparticle-based delivery systems enhance antioxidant bioavailability and target oxidative damage within the CNS [476,477,478]. Compounds that activate the Nrf2 signaling pathway, such as sulforaphane and dimethyl fumarate, promote the expression of endogenous antioxidant enzymes, providing long-term cellular protection [479,480]. Lipid-based antioxidants such as alpha-lipoic acid and hydrogen therapy, which selectively reduce toxic ROS, offer additional avenues for mitigating oxidative stress and preserving endothelial cell function [481,482,483]. Targeting mitochondrial dysfunction with pharmacological chaperones to reduce ROS production at its source is another promising approach [484,485,486]. Furthermore, combination therapies pairing antioxidants with anti-inflammatory agents, such as curcumin, show synergistic effects in reducing oxidative and inflammatory damage [487]. These advanced strategies highlight the potential for comprehensive antioxidant approaches to stabilize the BBB and slow the progression of neurodegenerative diseases.

### 5.3. Modulation of Tight Junction Proteins

The integrity of the BBB heavily relies on tight junction proteins, such as claudin-5, occludin, and ZO-1, which prevent paracellular leakage [21,25,216]. Strategies to strengthen or restore tight junctions are therefore promising for preserving BBB function [21,24,328].

Various therapeutic approaches are being explored to enhance BBB integrity by targeting tight junction proteins, reducing permeability, and stabilizing the barrier [21,24,328]. Agents that upregulate tight junction protein expression, e.g., glucocorticoids, can strengthen the BBB by increasing the expression of proteins, such as occludin and claudin. However, their long-term use may be associated with side effects [488,489,490]. Caffeine also shows promise for BBB support; it upregulates tight junction proteins, such as claudin-5 and ZO-1, improving endothelial cell cohesion and reducing permeability [408,491]. Its antioxidant and anti-inflammatory properties also help protect tight junctions from oxidative stress and inflammation—factors contributing to BBB breakdown [492,493]. VEGF inhibition is another approach, as VEGF disrupts tight junctions and increases BBB permeability in neurodegenerative diseases. Specific antagonists or antibodies targeting VEGF may reduce leakage and support barrier stability [494,495,496]. Lastly, MMPs degrade tight junction proteins and basement membrane components, weakening the BBB. MMP inhibitors, such as doxycycline, reduce MMP activity, thereby preserving tight junction integrity and further promoting BBB stability [132,259,459]. Together, these interventions may help lower the progression of neurodegenerative diseases by reinforcing the BBB [6,184,497]. Emerging strategies to modulate tight junction proteins focus on enhancing BBB integrity through innovative approaches. Small molecules targeting tight junction signaling pathways, such as Wnt/β-catenin activators, and peptide-based therapies that mimic tight junction domains show promise in strengthening BBB stability [253,498]. Natural compounds such as flavonoids (e.g., baicalin and quercetin) offer dual antioxidant and anti-inflammatory benefits while enhancing tight junction expression [499,500,501]. These advancements offer promising avenues to preserve BBB integrity and mitigate neurodegeneration.

### 5.4. Enhancement of BBB Transport Mechanisms

Efficient transport across the BBB is essential for delivering nutrients and clearing waste products [19,181,502]. Therapeutic approaches to enhance or restore CMT, RMT, and efflux systems are being explored to improve BBB function [342,343,344].

Enhancing transporter function at the BBB is a promising strategy for supporting neuronal health and maintaining BBB integrity in neurodegenerative diseases [6,8,184]. The upregulation of GLUT1 can improve glucose transport across the BBB, restoring energy balance in the CNS. This can be particularly effective in AD, where glucose transport is often impaired [345,346,419]. Efflux transporters, such as P-gp, play a crucial role in removing neurotoxic substances from the brain. Increasing P-gp activity may help clear harmful proteins, such as Aβ, thereby reducing the brain’s toxic burden and supporting BBB stability [378,503,504]. Additionally, amino acid transporters, such as LAT1, facilitate the passage of essential amino acids, which are necessary for neurotransmitter synthesis [158,159,505]. Enhancing these transporters can increase the availability of key precursors for neurotransmitter production, contributing to neuronal health and further reinforcing BBB integrity [158,159,505]. Emerging strategies to enhance BBB transport mechanisms aim to optimize nutrient delivery, waste clearance, and therapeutic drug delivery in neurodegenerative diseases. Lipid transporters, such as those facilitating omega-3 fatty acid delivery, and iron transporters, such as transferrin receptor 1, support neuronal health and prevent oxidative damage [506,507,508]. The modulation of efflux transporters, including P-gp and BCRP, is being investigated to balance the clearance of neurotoxins while enhancing therapeutic efficacy [509,510,511]. Peptide-based therapeutics that mimic natural ligands are also being studied to exploit endogenous transport systems, such as amino acid or glucose transporters [512]. Additionally, gut microbiota-derived metabolites, such as short-chain fatty acids, are being explored for their potential to influence BBB transporter activity, highlighting the interplay between diet, microbiota, and CNS health [513,514,515].

### 5.5. Neuroprotective Agents Targeting BBB Stability

Neuroprotective agents that specifically target endothelial cell health and BBB stability are being developed to protect against BBB damage in neurodegenerative diseases [5,16,516].

Several therapeutic approaches are being explored to support BBB integrity through anti-inflammatory, antioxidant, and neuroprotective mechanisms [447,517]. Angiotensin receptor blockers (ARBs), such as losartan, may help protect BBB stability by reducing oxidative stress and inflammation in endothelial cells. In preclinical studies, ARBs have shown potential in mitigating BBB disruption under chronic inflammatory conditions [518,519,520]. Statins, primarily known for their cholesterol-lowering effects, also exhibit anti-inflammatory and antioxidant properties. They may reduce BBB permeability by protecting endothelial cells and lowering inflammation, making them a promising therapeutic option for neurodegenerative diseases [521,522,523]. Moreover, hormone-based therapies, particularly estrogen-based ones, have shown potential in maintaining BBB integrity by supporting tight junction protein expression and reducing inflammation. This approach could benefit postmenopausal women at increased risk for neurodegeneration [524,525]. Additionally, neurotrophic factors, such as brain-derived neurotrophic factor (BDNF) and GDNF, support neuronal and neurovascular health, potentially reinforcing BBB integrity [188,451,526]. While direct delivery of these agents to the brain is challenging, innovative approaches, such as gene therapy and nanoparticle-based delivery systems, are being explored to deliver these neurotrophic factors effectively [527,528,529]. Emerging neuroprotective agents are expanding the scope of strategies targeting BBB stability in neurodegenerative diseases. Endothelin receptor antagonists, such as bosentan, reduce BBB permeability by counteracting vascular inflammation and oxidative stress [530,531]. Similarly, S1P receptor modulators such as fingolimod stabilize endothelial junctions and decrease immune cell trafficking, supporting BBB integrity [532,533,534]. Cerebroprotective peptides, such as angiotensin 1–7, and mitochondrial-targeted therapies, including SS-31 (elamipretide), protect endothelial cells from oxidative damage and apoptosis [535,536,537]. Prostaglandin analogs, such as misoprostol, and dietary nutraceuticals, including quercetin and catechins, also show promise by reducing inflammation and oxidative stress while enhancing vascular health [538,539,540].

### 5.6. Other Physical Strategies for Enhancing BBB Protection

Physical strategies for BBB protection have gained attention as potential therapeutic approaches in neurological conditions [175,541]. Focused ultrasound with microbubbles enables the temporary and targeted opening of the BBB, facilitating drug delivery while minimizing damage to surrounding tissues [542,543,544]. Hyperbaric oxygen therapy has been shown to enhance BBB integrity by reducing oxidative stress, inflammation, and edema [545,546,547]. Additionally, therapeutic hypothermia stabilizes endothelial cells and decreases inflammation, particularly during acute brain injuries such as ischemic stroke [548,549,550]. Emerging methods, such as electromagnetic field therapy, have demonstrated potential in modulating BBB permeability, while mechanical barriers, such as stents or implantable scaffolds, can indirectly protect the BBB by supporting vascular structures [551,552,553]. These strategies provide promising avenues for enhancing BBB protection and repair in various neurological disorders.

## 6. Conclusions

The BBB is crucial in protecting the brain from harmful substances and maintaining the precise microenvironment necessary for proper neuronal function. In neurodegenerative diseases, the disruption of BBB integrity is a critical contributor to disease progression, allowing neurotoxic molecules, inflammatory cells, and pathological proteins to infiltrate the brain. This review highlights the BBB’s central role in mitigating the progression of neurodegenerative diseases, emphasizing that preserving its function can slow neurodegeneration and improve patient outcomes. A deeper understanding of the molecular and cellular mechanisms underlying BBB breakdown, coupled with the development of targeted therapeutic strategies, is essential for protecting the BBB and reducing the burden of these debilitating conditions. Therefore, by prioritizing the maintenance of a healthy BBB, we can uncover novel approaches to prevent or delay the onset and progression of neurodegenerative diseases, offering hope for improved quality of life for affected individuals.

## References

[1] Pathak N., Vimal S.K., Tandon I., Agrawal L., Hongyi C., Bhattacharyya S. (2022). Neurodegenerative Disorders of Alzheimer, Parkinsonism, Amyotrophic Lateral Sclerosis and Multiple Sclerosis: An Early Diagnostic Approach for Precision Treatment. Metab. Brain Dis..

[2] Ji Z., Chen Q., Yang J., Hou J., Wu H., Zhang L. (2024). Global, regional, and national health inequalities of Alzheimer’s disease and Parkinson’s disease in 204 countries, 1990–2019. Int. J. Equity Health.

[3] Nian K., Harding I.C., Herman I.M., Ebong E.E. (2020). Blood-Brain Barrier Damage in Ischemic Stroke and Its Regulation by Endothelial Mechanotransduction. Front. Physiol..

[4] Sweeney M.D., Sagare A.P., Zlokovic B.V. (2018). Blood-brain barrier breakdown in Alzheimer disease and other neurodegenerative disorders. Nat. Rev. Neurol..

[5] Yuan Y., Sun J., Dong Q., Cui M. (2023). Blood-brain barrier endothelial cells in neurodegenerative diseases: Signals from the “barrier”. Front. Neurosci..

[6] Chen T., Dai Y., Hu C., Lin Z., Wang S., Yang J., Zeng L., Li S., Li W. (2024). Cellular and molecular mechanisms of the blood-brain barrier dysfunction in neurodegenerative diseases. Fluids Barriers CNS.

[7] Abbott N.J., Patabendige A.A., Dolman D.E., Yusof S.R., Begley D.J. (2010). Structure and function of the blood-brain barrier. Neurobiol. Dis..

[8] Alajangi H.K., Kaur M., Sharma A., Rana S., Thakur S., Chatterjee M., Singla N., Jaiswal P.K., Singh G., Barnwal R.P. (2022). Blood-brain barrier: Emerging trends on transport models and new-age strategies for therapeutics intervention against neurological disorders. Mol. Brain.

[9] Wu D., Chen Q., Chen X., Han F., Chen Z., Wang Y. (2023). The blood-brain barrier: Structure, regulation, and drug delivery. Signal Transduct. Target. Ther..

[10] Persidsky Y., Ramirez S.H., Haorah J., Kanmogne G.D. (2006). Blood-brain barrier: Structural components and function under physiologic and pathologic conditions. J. Neuroimmune Pharmacol..

[11] Kadry H., Noorani B., Cucullo L. (2020). A blood-brain barrier overview on structure, function, impairment, and biomarkers of integrity. Fluids Barriers CNS.

[12] Takata F., Nakagawa S., Matsumoto J., Dohgu S. (2021). Blood-Brain Barrier Dysfunction Amplifies the Development of Neuroinflammation: Understanding of Cellular Events in Brain Microvascular Endothelial Cells for Prevention and Treatment of BBB Dysfunction. Front. Cell. Neurosci..

[13] Adamu A., Li S., Gao F., Xue G. (2024). The role of neuroinflammation in neurodegenerative diseases: Current understanding and future therapeutic targets. Front. Aging Neurosci..

[14] Kim S., Sharma C., Jung U.J., Kim S.R. (2023). Pathophysiological Role of Microglial Activation Induced by Blood-Borne Proteins in Alzheimer’s Disease. Biomedicines.

[15] Uprety A., Kang Y., Kim S.Y. (2021). Blood-brain barrier dysfunction as a potential therapeutic target for neurodegenerative disorders. Arch. Pharm. Res..

[16] Mhaske A., Shukla S., Ahirwar K., Singh K.K., Shukla R. (2024). Receptor-Assisted Nanotherapeutics for Overcoming the Blood-Brain Barrier. Mol. Neurobiol..

[17] Alahmari A. (2021). Blood-Brain Barrier Overview: Structural and Functional Correlation. Neural Plast..

[18] McConnell H.L., Mishra A. (2022). Cells of the Blood-Brain Barrier: An Overview of the Neurovascular Unit in Health and Disease. Methods Mol. Biol..

[19] Sanchez-Cano F., Hernandez-Kelly L.C., Ortega A. (2021). The Blood-Brain Barrier: Much More Than a Selective Access to the Brain. Neurotox. Res..

[20] Xu L., Nirwane A., Yao Y. (2019). Basement membrane and blood-brain barrier. Stroke Vasc. Neurol..

[21] Lochhead J.J., Yang J., Ronaldson P.T., Davis T.P. (2020). Structure, Function, and Regulation of the Blood-Brain Barrier Tight Junction in Central Nervous System Disorders. Front. Physiol..

[22] Villasenor R., Lampe J., Schwaninger M., Collin L. (2019). Intracellular transport and regulation of transcytosis across the blood-brain barrier. Cell. Mol. Life Sci..

[23] Berndt P., Winkler L., Cording J., Breitkreuz-Korff O., Rex A., Dithmer S., Rausch V., Blasig R., Richter M., Sporbert A. (2019). Tight junction proteins at the blood-brain barrier: Far more than claudin-5. Cell. Mol. Life Sci..

[24] Luissint A.C., Artus C., Glacial F., Ganeshamoorthy K., Couraud P.O. (2012). Tight junctions at the blood brain barrier: Physiological architecture and disease-associated dysregulation. Fluids Barriers CNS.

[25] Greene C., Hanley N., Campbell M. (2019). Claudin-5: Gatekeeper of neurological function. Fluids Barriers CNS.

[26] Hu Y.J., Wang Y.D., Tan F.Q., Yang W.X. (2013). Regulation of paracellular permeability: Factors and mechanisms. Mol. Biol. Rep..

[27] Traweger A., Fuchs R., Krizbai I.A., Weiger T.M., Bauer H.C., Bauer H. (2003). The tight junction protein ZO-2 localizes to the nucleus and interacts with the heterogeneous nuclear ribonucleoprotein scaffold attachment factor-B. J. Biol. Chem..

[28] Bhalerao A., Sivandzade F., Archie S.R., Chowdhury E.A., Noorani B., Cucullo L. (2020). In vitro modeling of the neurovascular unit: Advances in the field. Fluids Barriers CNS.

[29] Geranmayeh M.H., Rahbarghazi R., Farhoudi M. (2019). Targeting pericytes for neurovascular regeneration. Cell Commun. Signal..

[30] Kugler E.C., Greenwood J., MacDonald R.B. (2021). The “Neuro-Glial-Vascular” Unit: The Role of Glia in Neurovascular Unit Formation and Dysfunction. Front. Cell Dev. Biol..

[31] Pivoriunas A., Verkhratsky A. (2021). Astrocyte-Endotheliocyte Axis in the Regulation of the Blood-Brain Barrier. Neurochem. Res..

[32] Manu D.R., Slevin M., Barcutean L., Forro T., Boghitoiu T., Balasa R. (2023). Astrocyte Involvement in Blood-Brain Barrier Function: A Critical Update Highlighting Novel, Complex, Neurovascular Interactions. Int. J. Mol. Sci..

[33] Yang L., Lin Z., Mu R., Wu W., Zhi H., Liu X., Yang H., Liu L. (2024). Neurons enhance blood-brain barrier function via upregulating claudin-5 and VE-cadherin expression due to glial cell line-derived neurotrophic factor secretion. eLife.

[34] Armulik A., Genove G., Mae M., Nisancioglu M.H., Wallgard E., Niaudet C., He L., Norlin J., Lindblom P., Strittmatter K. (2010). Pericytes regulate the blood-brain barrier. Nature.

[35] Rustenhoven J., Aalderink M., Scotter E.L., Oldfield R.L., Bergin P.S., Mee E.W., Graham E.S., Faull R.L., Curtis M.A., Park T.I. (2016). TGF-beta1 regulates human brain pericyte inflammatory processes involved in neurovasculature function. J. Neuroinflamm..

[36] Tjakra M., Wang Y., Vania V., Hou Z., Durkan C., Wang N., Wang G. (2019). Overview of Crosstalk Between Multiple Factor of Transcytosis in Blood Brain Barrier. Front. Neurosci..

[37] Villabona-Rueda A., Erice C., Pardo C.A., Stins M.F. (2019). The Evolving Concept of the Blood Brain Barrier (BBB): From a Single Static Barrier to a Heterogeneous and Dynamic Relay Center. Front. Cell. Neurosci..

[38] Kaplan L., Chow B.W., Gu C. (2020). Neuronal regulation of the blood-brain barrier and neurovascular coupling. Nat. Rev. Neurosci..

[39] Presa J.L., Saravia F., Bagi Z., Filosa J.A. (2020). Vasculo-Neuronal Coupling and Neurovascular Coupling at the Neurovascular Unit: Impact of Hypertension. Front. Physiol..

[40] Zolotoff C., Voirin A.C., Puech C., Roche F., Perek N. (2020). Intermittent Hypoxia and Its Impact on Nrf2/HIF-1alpha Expression and ABC Transporters: An in Vitro Human Blood-Brain Barrier Model Study. Cell. Physiol. Biochem..

[41] Gao H.M., Chen H., Cui G.Y., Hu J.X. (2023). Damage mechanism and therapy progress of the blood-brain barrier after ischemic stroke. Cell Biosci..

[42] Liu G., Wang Q., Tian L., Wang M., Duo D., Duan Y., Lin Y., Han J., Jia Q., Zhu J. (2024). Blood-Brain Barrier Permeability is Affected by Changes in Tight Junction Protein Expression at High-Altitude Hypoxic Conditions-this may have Implications for Brain Drug Transport. AAPS J..

[43] Sa-Pereira I., Brites D., Brito M.A. (2012). Neurovascular unit: A focus on pericytes. Mol. Neurobiol..

[44] Brown L.S., Foster C.G., Courtney J.M., King N.E., Howells D.W., Sutherland B.A. (2019). Pericytes and Neurovascular Function in the Healthy and Diseased Brain. Front. Cell. Neurosci..

[45] Hill J., Rom S., Ramirez S.H., Persidsky Y. (2014). Emerging roles of pericytes in the regulation of the neurovascular unit in health and disease. J. Neuroimmune Pharmacol..

[46] Eltanahy A.M., Koluib Y.A., Gonzales A. (2021). Pericytes: Intrinsic Transportation Engineers of the CNS Microcirculation. Front. Physiol..

[47] Heymans M., Figueiredo R., Dehouck L., Francisco D., Sano Y., Shimizu F., Kanda T., Bruggmann R., Engelhardt B., Winter P. (2020). Contribution of brain pericytes in blood-brain barrier formation and maintenance: A transcriptomic study of cocultured human endothelial cells derived from hematopoietic stem cells. Fluids Barriers CNS.

[48] Medina-Flores F., Hurtado-Alvarado G., Deli M.A., Gomez-Gonzalez B. (2023). The Active Role of Pericytes During Neuroinflammation in the Adult Brain. Cell. Mol. Neurobiol..

[49] Stratman A.N., Malotte K.M., Mahan R.D., Davis M.J., Davis G.E. (2009). Pericyte recruitment during vasculogenic tube assembly stimulates endothelial basement membrane matrix formation. Blood.

[50] Stratman A.N., Davis G.E. (2012). Endothelial cell-pericyte interactions stimulate basement membrane matrix assembly: Influence on vascular tube remodeling, maturation, and stabilization. Microsc. Microanal..

[51] Reed M.J., Damodarasamy M., Banks W.A. (2019). The extracellular matrix of the blood-brain barrier: Structural and functional roles in health, aging, and Alzheimer’s disease. Tissue Barriers.

[52] Daneman R., Zhou L., Kebede A.A., Barres B.A. (2010). Pericytes are required for blood-brain barrier integrity during embryogenesis. Nature.

[53] Fernandez-Klett F., Priller J. (2015). Diverse functions of pericytes in cerebral blood flow regulation and ischemia. J. Cereb. Blood Flow. Metab..

[54] Dalkara T., Ostergaard L., Heusch G., Attwell D. (2024). Pericytes in the brain and heart: Functional roles and response to ischemia and reperfusion. Cardiovasc. Res..

[55] Alarcon-Martinez L., Yilmaz-Ozcan S., Yemisci M., Schallek J., Kilic K., Can A., Di Polo A., Dalkara T. (2018). Capillary pericytes express alpha-smooth muscle actin, which requires prevention of filamentous-actin depolymerization for detection. eLife.

[56] Csipo T., Lipecz A., Mukli P., Bahadli D., Abdulhussein O., Owens C.D., Tarantini S., Hand R.A., Yabluchanska V., Kellawan J.M. (2021). Increased cognitive workload evokes greater neurovascular coupling responses in healthy young adults. PLoS ONE.

[57] Zhu W.M., Neuhaus A., Beard D.J., Sutherland B.A., DeLuca G.C. (2022). Neurovascular coupling mechanisms in health and neurovascular uncoupling in Alzheimer’s disease. Brain.

[58] Feng L., Gao L. (2024). The role of neurovascular coupling dysfunction in cognitive decline of diabetes patients. Front. Neurosci..

[59] Payne L.B., Hoque M., Houk C., Darden J., Chappell J.C. (2020). Pericytes in Vascular Development. Curr. Tissue Microenviron. Rep..

[60] Armulik A., Mae M., Betsholtz C. (2011). Pericytes and the blood-brain barrier: Recent advances and implications for the delivery of CNS therapy. Ther. Deliv..

[61] Laredo F., Plebanski J., Tedeschi A. (2019). Pericytes: Problems and Promises for CNS Repair. Front. Cell. Neurosci..

[62] Jansson D., Rustenhoven J., Feng S., Hurley D., Oldfield R.L., Bergin P.S., Mee E.W., Faull R.L., Dragunow M. (2014). A role for human brain pericytes in neuroinflammation. J. Neuroinflamm..

[63] Cai W., Liu H., Zhao J., Chen L.Y., Chen J., Lu Z., Hu X. (2017). Pericytes in Brain Injury and Repair After Ischemic Stroke. Transl. Stroke Res..

[64] Kovac A., Erickson M.A., Banks W.A. (2011). Brain microvascular pericytes are immunoactive in culture: Cytokine, chemokine, nitric oxide, and LRP-1 expression in response to lipopolysaccharide. J. Neuroinflamm..

[65] Navarro R., Compte M., Alvarez-Vallina L., Sanz L. (2016). Immune Regulation by Pericytes: Modulating Innate and Adaptive Immunity. Front. Immunol..

[66] Cheng J., Korte N., Nortley R., Sethi H., Tang Y., Attwell D. (2018). Targeting pericytes for therapeutic approaches to neurological disorders. Acta Neuropathol..

[67] Nakamura K., Ago T. (2023). Pericyte-Mediated Molecular Mechanisms Underlying Tissue Repair and Functional Recovery after Ischemic Stroke. J. Atheroscler. Thromb..

[68] Sweeney M.D., Ayyadurai S., Zlokovic B.V. (2016). Pericytes of the neurovascular unit: Key functions and signaling pathways. Nat. Neurosci..

[69] Kaushik D.K., Bhattacharya A., Lozinski B.M., Wee Yong V. (2021). Pericytes as mediators of infiltration of macrophages in multiple sclerosis. J. Neuroinflamm..

[70] Teske N.C., Dyckhoff-Shen S., Beckenbauer P., Bewersdorf J.P., Engelen-Lee J.Y., Hammerschmidt S., Kalin R.E., Pfister H.W., Brouwer M.C., Klein M. (2023). Pericytes are protective in experimental pneumococcal meningitis through regulating leukocyte infiltration and blood-brain barrier function. J. Neuroinflamm..

[71] Torok O., Schreiner B., Schaffenrath J., Tsai H.C., Maheshwari U., Stifter S.A., Welsh C., Amorim A., Sridhar S., Utz S.G. (2021). Pericytes regulate vascular immune homeostasis in the CNS. Proc. Natl. Acad. Sci. USA.

[72] Mills W.A., Woo A.M., Jiang S., Martin J., Surendran D., Bergstresser M., Kimbrough I.F., Eyo U.B., Sofroniew M.V., Sontheimer H. (2022). Astrocyte plasticity in mice ensures continued endfoot coverage of cerebral blood vessels following injury and declines with age. Nat. Commun..

[73] Benarroch E.E. (2005). Neuron-astrocyte interactions: Partnership for normal function and disease in the central nervous system. Mayo Clin. Proc..

[74] Abbott N.J., Ronnback L., Hansson E. (2006). Astrocyte-endothelial interactions at the blood-brain barrier. Nat. Rev. Neurosci..

[75] Jensen C.J., Massie A., De Keyser J. (2013). Immune players in the CNS: The astrocyte. J. Neuroimmune Pharmacol..

[76] Sofroniew M.V., Vinters H.V. (2010). Astrocytes: Biology and pathology. Acta Neuropathol..

[77] Zhao Y., Huang Y., Cao Y., Yang J. (2024). Astrocyte-Mediated Neuroinflammation in Neurological Conditions. Biomolecules.

[78] Overgaard Wichmann T., Hedegaard Hojsager M., Hasager Damkier H. (2024). Water channels in the brain and spinal cord-overview of the role of aquaporins in traumatic brain injury and traumatic spinal cord injury. Front. Cell. Neurosci..

[79] Liu X., Li C., Li J., Xie L., Hong Z., Zheng K., Zhao X., Yang A., Xu X., Tao H. (2022). EGF signaling promotes the lineage conversion of astrocytes into oligodendrocytes. Mol. Med..

[80] Qu X., Yang R., Tan C., Chen H., Wang X. (2024). Astrocytes-Secreted WNT5B Disrupts the Blood-Brain Barrier Via ROR1/JNK/c-JUN Cascade During Meningitic Escherichia Coli Infection. Mol. Neurobiol..

[81] Dozio V., Sanchez J.C. (2018). Profiling the proteomic inflammatory state of human astrocytes using DIA mass spectrometry. J. Neuroinflamm..

[82] Alsbrook D.L., Di Napoli M., Bhatia K., Biller J., Andalib S., Hinduja A., Rodrigues R., Rodriguez M., Sabbagh S.Y., Selim M. (2023). Neuroinflammation in Acute Ischemic and Hemorrhagic Stroke. Curr. Neurol. Neurosci. Rep..

[83] Vandebroek A., Yasui M. (2020). Regulation of AQP4 in the Central Nervous System. Int. J. Mol. Sci..

[84] Salman M.M., Kitchen P., Halsey A., Wang M.X., Tornroth-Horsefield S., Conner A.C., Badaut J., Iliff J.J., Bill R.M. (2022). Emerging roles for dynamic aquaporin-4 subcellular relocalization in CNS water homeostasis. Brain.

[85] Zhou Z., Zhan J., Cai Q., Xu F., Chai R., Lam K., Luan Z., Zhou G., Tsang S., Kipp M. (2022). The Water Transport System in Astrocytes-Aquaporins. Cells.

[86] Tang G., Yang G.Y. (2016). Aquaporin-4: A Potential Therapeutic Target for Cerebral Edema. Int. J. Mol. Sci..

[87] Song Y., Gunnarson E. (2012). Potassium dependent regulation of astrocyte water permeability is mediated by cAMP signaling. PLoS ONE.

[88] Kinboshi M., Ikeda A., Ohno Y. (2020). Role of Astrocytic Inwardly Rectifying Potassium (Kir) 4.1 Channels in Epileptogenesis. Front. Neurol..

[89] Murakami S., Kurachi Y. (2016). Mechanisms of astrocytic K(+) clearance and swelling under high extracellular K(+) concentrations. J. Physiol. Sci..

[90] Bellot-Saez A., Stevenson R., Kekesi O., Samokhina E., Ben-Abu Y., Morley J.W., Buskila Y. (2021). Neuromodulation of Astrocytic K(+) Clearance. Int. J. Mol. Sci..

[91] Falkowska A., Gutowska I., Goschorska M., Nowacki P., Chlubek D., Baranowska-Bosiacka I. (2015). Energy Metabolism of the Brain, Including the Cooperation between Astrocytes and Neurons, Especially in the Context of Glycogen Metabolism. Int. J. Mol. Sci..

[92] Mason S. (2017). Lactate Shuttles in Neuroenergetics-Homeostasis, Allostasis and Beyond. Front. Neurosci..

[93] Beard E., Lengacher S., Dias S., Magistretti P.J., Finsterwald C. (2021). Astrocytes as Key Regulators of Brain Energy Metabolism: New Therapeutic Perspectives. Front. Physiol..

[94] Cai M., Wang H., Song H., Yang R., Wang L., Xue X., Sun W., Hu J. (2022). Lactate Is Answerable for Brain Function and Treating Brain Diseases: Energy Substrates and Signal Molecule. Front. Nutr..

[95] Xue X., Liu B., Hu J., Bian X., Lou S. (2022). The potential mechanisms of lactate in mediating exercise-enhanced cognitive function: A dual role as an energy supply substrate and a signaling molecule. Nutr. Metab..

[96] Wu A., Lee D., Xiong W.C. (2023). Lactate Metabolism, Signaling, and Function in Brain Development, Synaptic Plasticity, Angiogenesis, and Neurodegenerative Diseases. Int. J. Mol. Sci..

[97] Schousboe A., Scafidi S., Bak L.K., Waagepetersen H.S., McKenna M.C. (2014). Glutamate metabolism in the brain focusing on astrocytes. Adv. Neurobiol..

[98] Sidoryk-Wegrzynowicz M., Adamiak K., Struzynska L. (2024). Astrocyte-Neuron Interaction via the Glutamate-Glutamine Cycle and Its Dysfunction in Tau-Dependent Neurodegeneration. Int. J. Mol. Sci..

[99] Mahmoud S., Gharagozloo M., Simard C., Gris D. (2019). Astrocytes Maintain Glutamate Homeostasis in the CNS by Controlling the Balance between Glutamate Uptake and Release. Cells.

[100] Peterson A.R., Binder D.K. (2020). Astrocyte Glutamate Uptake and Signaling as Novel Targets for Antiepileptogenic Therapy. Front. Neurol..

[101] Cuellar-Santoyo A.O., Ruiz-Rodriguez V.M., Mares-Barbosa T.B., Patron-Soberano A., Howe A.G., Portales-Perez D.P., Miquelajauregui Graf A., Estrada-Sanchez A.M. (2022). Revealing the contribution of astrocytes to glutamatergic neuronal transmission. Front. Cell. Neurosci..

[102] Armada-Moreira A., Gomes J.I., Pina C.C., Savchak O.K., Goncalves-Ribeiro J., Rei N., Pinto S., Morais T.P., Martins R.S., Ribeiro F.F. (2020). Going the Extra (Synaptic) Mile: Excitotoxicity as the Road Toward Neurodegenerative Diseases. Front. Cell. Neurosci..

[103] Belov Kirdajova D., Kriska J., Tureckova J., Anderova M. (2020). Ischemia-Triggered Glutamate Excitotoxicity From the Perspective of Glial Cells. Front. Cell. Neurosci..

[104] Thomsen M.S., Routhe L.J., Moos T. (2017). The vascular basement membrane in the healthy and pathological brain. J. Cereb. Blood Flow. Metab..

[105] Soles A., Selimovic A., Sbrocco K., Ghannoum F., Hamel K., Moncada E.L., Gilliat S., Cvetanovic M. (2023). Extracellular Matrix Regulation in Physiology and in Brain Disease. Int. J. Mol. Sci..

[106] Mak K.M., Mei R. (2017). Basement Membrane Type IV Collagen and Laminin: An Overview of Their Biology and Value as Fibrosis Biomarkers of Liver Disease. Anat. Rec..

[107] Roig-Rosello E., Rousselle P. (2020). The Human Epidermal Basement Membrane: A Shaped and Cell Instructive Platform That Aging Slowly Alters. Biomolecules.

[108] Wareham L.K., Baratta R.O., Del Buono B.J., Schlumpf E., Calkins D.J. (2024). Collagen in the central nervous system: Contributions to neurodegeneration and promise as a therapeutic target. Mol. Neurodegener..

[109] Yurchenco P.D. (2011). Basement membranes: Cell scaffoldings and signaling platforms. Cold Spring Harb. Perspect. Biol..

[110] Leclech C., Natale C.F., Barakat A.I. (2020). The basement membrane as a structured surface—Role in vascular health and disease. J. Cell Sci..

[111] Topfer U. (2023). Basement membrane dynamics and mechanics in tissue morphogenesis. Biol. Open.

[112] Glentis A., Gurchenkov V., Matic Vignjevic D. (2014). Assembly, heterogeneity, and breaching of the basement membranes. Cell Adhes. Migr..

[113] Naylor R.W., Morais M., Lennon R. (2021). Complexities of the glomerular basement membrane. Nat. Rev. Nephrol..

[114] Aguilera K.Y., Brekken R.A. (2014). Recruitment and retention: Factors that affect pericyte migration. Cell. Mol. Life Sci..

[115] Potapova I.A., Gaudette G.R., Brink P.R., Robinson R.B., Rosen M.R., Cohen I.S., Doronin S.V. (2007). Mesenchymal stem cells support migration, extracellular matrix invasion, proliferation, and survival of endothelial cells in vitro. Stem Cells.

[116] Omorphos N.P., Gao C., Tan S.S., Sangha M.S. (2021). Understanding angiogenesis and the role of angiogenic growth factors in the vascularisation of engineered tissues. Mol. Biol. Rep..

[117] Gautam J., Zhang X., Yao Y. (2016). The role of pericytic laminin in blood brain barrier integrity maintenance. Sci. Rep..

[118] Halder S.K., Sapkota A., Milner R. (2022). The impact of genetic manipulation of laminin and integrins at the blood-brain barrier. Fluids Barriers CNS.

[119] Knox E.G., Aburto M.R., Clarke G., Cryan J.F., O’Driscoll C.M. (2022). The blood-brain barrier in aging and neurodegeneration. Mol. Psychiatry.

[120] Kelley L.C., Lohmer L.L., Hagedorn E.J., Sherwood D.R. (2014). Traversing the basement membrane in vivo: A diversity of strategies. J. Cell Biol..

[121] Chang J., Chaudhuri O. (2019). Beyond proteases: Basement membrane mechanics and cancer invasion. J. Cell Biol..

[122] Ghannam S.F., Rutland C.S., Allegrucci C., Mongan N.P., Rakha E. (2023). Defining invasion in breast cancer: The role of basement membrane. J. Clin. Pathol..

[123] Engelhardt B., Sorokin L. (2009). The blood-brain and the blood-cerebrospinal fluid barriers: Function and dysfunction. Semin. Immunopathol..

[124] Zhang X., Wang Y., Song J., Gerwien H., Chuquisana O., Chashchina A., Denz C., Sorokin L. (2020). The endothelial basement membrane acts as a checkpoint for entry of pathogenic T cells into the brain. J. Exp. Med..

[125] Kangwantas K., Pinteaux E., Penny J. (2016). The extracellular matrix protein laminin-10 promotes blood-brain barrier repair after hypoxia and inflammation in vitro. J. Neuroinflamm..

[126] Archie S.R., Al Shoyaib A., Cucullo L. (2021). Blood-Brain Barrier Dysfunction in CNS Disorders and Putative Therapeutic Targets: An Overview. Pharmaceutics.

[127] Nguyen B., Bix G., Yao Y. (2021). Basal lamina changes in neurodegenerative disorders. Mol. Neurodegener..

[128] Wu Y.C., Bogale T.A., Koistinaho J., Pizzi M., Rolova T., Bellucci A. (2024). The contribution of beta-amyloid, Tau and alpha-synuclein to blood-brain barrier damage in neurodegenerative disorders. Acta Neuropathol..

[129] Lukes A., Mun-Bryce S., Lukes M., Rosenberg G.A. (1999). Extracellular matrix degradation by metalloproteinases and central nervous system diseases. Mol. Neurobiol..

[130] Behl T., Kaur G., Sehgal A., Bhardwaj S., Singh S., Buhas C., Judea-Pusta C., Uivarosan D., Munteanu M.A., Bungau S. (2021). Multifaceted Role of Matrix Metalloproteinases in Neurodegenerative Diseases: Pathophysiological and Therapeutic Perspectives. Int. J. Mol. Sci..

[131] Radosinska D., Radosinska J. (2025). The Link Between Matrix Metalloproteinases and Alzheimer’s Disease Pathophysiology. Mol. Neurobiol..

[132] Ahmadighadykolaei H., Lambert J.A., Raeeszadeh-Sarmazdeh M. (2023). TIMP-1 Protects Tight Junctions of Brain Endothelial Cells From MMP-Mediated Degradation. Pharm. Res..

[133] Obermeier B., Daneman R., Ransohoff R.M. (2013). Development, maintenance and disruption of the blood-brain barrier. Nat. Med..

[134] Yuan Y., Liu H., Dai Z., He C., Qin S., Su Z. (2024). From Physiology to Pathology of Astrocytes: Highlighting Their Potential as Therapeutic Targets for CNS Injury. Neurosci. Bull..

[135] Ahmad A., Patel V., Xiao J., Khan M.M. (2020). The Role of Neurovascular System in Neurodegenerative Diseases. Mol. Neurobiol..

[136] Yu X., Ji C., Shao A. (2020). Neurovascular Unit Dysfunction and Neurodegenerative Disorders. Front. Neurosci..

[137] Daneman R., Prat A. (2015). The blood-brain barrier. Cold Spring Harb. Perspect. Biol..

[138] Viscusi E.R., Viscusi A.R. (2020). Blood-brain barrier: Mechanisms governing permeability and interaction with peripherally acting mu-opioid receptor antagonists. Reg. Anesth. Pain. Med..

[139] Sousa J.A., Bernardes C., Bernardo-Castro S., Lino M., Albino I., Ferreira L., Bras J., Guerreiro R., Tabuas-Pereira M., Baldeiras I. (2023). Reconsidering the role of blood-brain barrier in Alzheimer’s disease: From delivery to target. Front. Aging Neurosci..

[140] Al-Obaidi M.M.J., Desa M.N.M. (2018). Mechanisms of Blood Brain Barrier Disruption by Different Types of Bacteria, and Bacterial-Host Interactions Facilitate the Bacterial Pathogen Invading the Brain. Cell. Mol. Neurobiol..

[141] Le Govic Y., Demey B., Cassereau J., Bahn Y.S., Papon N. (2022). Pathogens infecting the central nervous system. PLoS Pathog..

[142] Rani A., Ergun S., Karnati S., Jha H.C. (2024). Understanding the link between neurotropic viruses, BBB permeability, and MS pathogenesis. J. NeuroVirology.

[143] Cain M.D., Salimi H., Diamond M.S., Klein R.S. (2019). Mechanisms of Pathogen Invasion into the Central Nervous System. Neuron.

[144] Galea I. (2021). The blood-brain barrier in systemic infection and inflammation. Cell. Mol. Immunol..

[145] DeMaio A., Mehrotra S., Sambamurti K., Husain S. (2022). The role of the adaptive immune system and T cell dysfunction in neurodegenerative diseases. J. Neuroinflamm..

[146] Butovsky O., Weiner H.L. (2018). Microglial signatures and their role in health and disease. Nat. Rev. Neurosci..

[147] Reyes-Martinez S., Segura-Real L., Gomez-Garcia A.P., Tesoro-Cruz E., Constantino-Jonapa L.A., Amedei A., Aguirre-Garcia M.M. (2023). Neuroinflammation, Microbiota-Gut-Brain Axis, and Depression: The Vicious Circle. J. Integr. Neurosci..

[148] Haddad-Tovolli R., Dragano N.R.V., Ramalho A.F.S., Velloso L.A. (2017). Development and Function of the Blood-Brain Barrier in the Context of Metabolic Control. Front. Neurosci..

[149] Redzic Z. (2011). Molecular biology of the blood-brain and the blood-cerebrospinal fluid barriers: Similarities and differences. Fluids Barriers CNS.

[150] Barber C.N., Raben D.M. (2019). Lipid Metabolism Crosstalk in the Brain: Glia and Neurons. Front. Cell. Neurosci..

[151] Afridi R., Kim J.H., Rahman M.H., Suk K. (2020). Metabolic Regulation of Glial Phenotypes: Implications in Neuron-Glia Interactions and Neurological Disorders. Front. Cell. Neurosci..

[152] Fong C.W. (2015). Permeability of the Blood-Brain Barrier: Molecular Mechanism of Transport of Drugs and Physiologically Important Compounds. J. Membr. Biol..

[153] Fu B.M. (2018). Transport Across the Blood-Brain Barrier. Adv. Exp. Med. Biol..

[154] Zaragoza R. (2020). Transport of Amino Acids Across the Blood-Brain Barrier. Front. Physiol..

[155] Park M.S. (2015). Molecular Dynamics Simulations of the Human Glucose Transporter GLUT1. PLoS ONE.

[156] Patching S.G. (2017). Glucose Transporters at the Blood-Brain Barrier: Function, Regulation and Gateways for Drug Delivery. Mol. Neurobiol..

[157] Koepsell H. (2020). Glucose transporters in brain in health and disease. Pflugers Arch..

[158] Puris E., Gynther M., Auriola S., Huttunen K.M. (2020). L-Type amino acid transporter 1 as a target for drug delivery. Pharm. Res..

[159] Ahmed H.S. (2024). The Multifaceted Role of L-Type Amino Acid Transporter 1 at the Blood-Brain Barrier: Structural Implications and Therapeutic Potential. Mol. Neurobiol..

[160] Brini M., Cali T., Ottolini D., Carafoli E. (2014). Neuronal calcium signaling: Function and dysfunction. Cell. Mol. Life Sci..

[161] Liu L., Liu X.D. (2014). Alterations in function and expression of ABC transporters at blood-brain barrier under diabetes and the clinical significances. Front. Pharmacol..

[162] Mahringer A., Fricker G. (2016). ABC transporters at the blood-brain barrier. Expert Opin. Drug Metab. Toxicol..

[163] Eng M.E., Imperio G.E., Bloise E., Matthews S.G. (2022). ATP-binding cassette (ABC) drug transporters in the developing blood-brain barrier: Role in fetal brain protection. Cell. Mol. Life Sci..

[164] Strazielle N., Ghersi-Egea J.F. (2015). Efflux transporters in blood-brain interfaces of the developing brain. Front. Neurosci..

[165] Gu M., Mei X.L., Zhao Y.N. (2021). Sepsis and Cerebral Dysfunction: BBB Damage, Neuroinflammation, Oxidative Stress, Apoptosis and Autophagy as Key Mediators and the Potential Therapeutic Approaches. Neurotox. Res..

[166] Feng Z., Fang C., Ma Y., Chang J. (2024). Obesity-induced blood-brain barrier dysfunction: Phenotypes and mechanisms. J. Neuroinflamm..

[167] Almutairi M.M., Gong C., Xu Y.G., Chang Y., Shi H. (2016). Factors controlling permeability of the blood-brain barrier. Cell. Mol. Life Sci..

[168] Schurhoff N., Toborek M. (2023). Circadian rhythms in the blood-brain barrier: Impact on neurological disorders and stress responses. Mol. Brain.

[169] Chen S., Chen H., Du Q., Shen J. (2020). Targeting Myeloperoxidase (MPO) Mediated Oxidative Stress and Inflammation for Reducing Brain Ischemia Injury: Potential Application of Natural Compounds. Front. Physiol..

[170] Gobel B., Oltmanns K.M., Chung M. (2013). Linking neuronal brain activity to the glucose metabolism. Theor. Biol. Med. Model..

[171] Watts M.E., Pocock R., Claudianos C. (2018). Brain Energy and Oxygen Metabolism: Emerging Role in Normal Function and Disease. Front. Mol. Neurosci..

[172] Ozugur S., Kunz L., Straka H. (2020). Relationship between oxygen consumption and neuronal activity in a defined neural circuit. BMC Biol..

[173] Petzold G.C., Murthy V.N. (2011). Role of astrocytes in neurovascular coupling. Neuron.

[174] Phillips A.A., Chan F.H., Zheng M.M., Krassioukov A.V., Ainslie P.N. (2016). Neurovascular coupling in humans: Physiology, methodological advances and clinical implications. J. Cereb. Blood Flow Metab..

[175] Malkiewicz M.A., Szarmach A., Sabisz A., Cubala W.J., Szurowska E., Winklewski P.J. (2019). Blood-brain barrier permeability and physical exercise. J. Neuroinflamm..

[176] Hoshi Y., Uchida Y., Tachikawa M., Ohtsuki S., Couraud P.O., Suzuki T., Terasaki T. (2020). Oxidative stress-induced activation of Abl and Src kinases rapidly induces P-glycoprotein internalization via phosphorylation of caveolin-1 on tyrosine-14, decreasing cortisol efflux at the blood-brain barrier. J. Cereb. Blood Flow Metab..

[177] Chai A.B., Callaghan R., Gelissen I.C. (2022). Regulation of P-Glycoprotein in the Brain. Int. J. Mol. Sci..

[178] Shchulkin A.V., Abalenikhina Y.V., Kosmachevskaya O.V., Topunov A.F., Yakusheva E.N. (2024). Regulation of P-Glycoprotein during Oxidative Stress. Antioxidants.

[179] Ibbotson K., Yell J., Ronaldson P.T. (2017). Nrf2 signaling increases expression of ATP-binding cassette subfamily C mRNA transcripts at the blood-brain barrier following hypoxia-reoxygenation stress. Fluids Barriers CNS.

[180] Sharma V., Singh T.G., Mannan A. (2022). Therapeutic implications of glucose transporters (GLUT) in cerebral ischemia. Neurochem. Res..

[181] Liu J., Guo Y., Zhang C., Zeng Y., Luo Y., Wang G. (2021). Clearance Systems in the Brain, From Structure to Function. Front. Cell. Neurosci..

[182] Villa M., Wu J., Hansen S., Pahnke J. (2024). Emerging Role of ABC Transporters in Glia Cells in Health and Diseases of the Central Nervous System. Cells.

[183] Noe C.R., Noe-Letschnig M., Handschuh P., Noe C.A., Lanzenberger R. (2020). Dysfunction of the Blood-Brain Barrier-A Key Step in Neurodegeneration and Dementia. Front. Aging Neurosci..

[184] Lau K., Kotzur R., Richter F. (2024). Blood-brain barrier alterations and their impact on Parkinson’s disease pathogenesis and therapy. Transl. Neurodegener..

[185] Serlin Y., Shelef I., Knyazer B., Friedman A. (2015). Anatomy and physiology of the blood-brain barrier. Semin. Cell Dev. Biol..

[186] Liebner S., Dijkhuizen R.M., Reiss Y., Plate K.H., Agalliu D., Constantin G. (2018). Functional morphology of the blood-brain barrier in health and disease. Acta Neuropathol..

[187] Benz F., Liebner S. (2022). Structure and Function of the Blood-Brain Barrier (BBB). Handb. Exp. Pharmacol..

[188] Xiao M., Xiao Z.J., Yang B., Lan Z., Fang F. (2020). Blood-Brain Barrier: More Contributor to Disruption of Central Nervous System Homeostasis Than Victim in Neurological Disorders. Front. Neurosci..

[189] Uttara B., Singh A.V., Zamboni P., Mahajan R.T. (2009). Oxidative stress and neurodegenerative diseases: A review of upstream and downstream antioxidant therapeutic options. Curr. Neuropharmacol..

[190] Melo A., Monteiro L., Lima R.M., Oliveira D.M., Cerqueira M.D., El-Bacha R.S. (2011). Oxidative stress in neurodegenerative diseases: Mechanisms and therapeutic perspectives. Oxidative Med. Cell. Longev..

[191] Liu Z., Zhou T., Ziegler A.C., Dimitrion P., Zuo L. (2017). Oxidative Stress in Neurodegenerative Diseases: From Molecular Mechanisms to Clinical Applications. Oxidative Med. Cell. Longev..

[192] Shadfar S., Parakh S., Jamali M.S., Atkin J.D. (2023). Redox dysregulation as a driver for DNA damage and its relationship to neurodegenerative diseases. Transl. Neurodegener..

[193] Kong J., Fan R., Zhang Y., Jia Z., Zhang J., Pan H., Wang Q. (2024). Oxidative stress in the brain-lung crosstalk: Cellular and molecular perspectives. Front. Aging Neurosci..

[194] Salvagno M., Sterchele E.D., Zaccarelli M., Mrakic-Sposta S., Welsby I.J., Balestra C., Taccone F.S. (2024). Oxidative Stress and Cerebral Vascular Tone: The Role of Reactive Oxygen and Nitrogen Species. Int. J. Mol. Sci..

[195] Freeman L.R., Keller J.N. (2012). Oxidative stress and cerebral endothelial cells: Regulation of the blood-brain-barrier and antioxidant based interventions. Biochim. Biophys. Acta.

[196] Chung T.D., Linville R.M., Guo Z., Ye R., Jha R., Grifno G.N., Searson P.C. (2022). Effects of acute and chronic oxidative stress on the blood-brain barrier in 2D and 3D in vitro models. Fluids Barriers CNS.

[197] Ray P.D., Huang B.W., Tsuji Y. (2012). Reactive oxygen species (ROS) homeostasis and redox regulation in cellular signaling. Cell. Signal..

[198] Jomova K., Raptova R., Alomar S.Y., Alwasel S.H., Nepovimova E., Kuca K., Valko M. (2023). Reactive oxygen species, toxicity, oxidative stress, and antioxidants: Chronic diseases and aging. Arch. Toxicol..

[199] Bustamante-Barrientos F.A., Luque-Campos N., Araya M.J., Lara-Barba E., de Solminihac J., Pradenas C., Molina L., Herrera-Luna Y., Utreras-Mendoza Y., Elizondo-Vega R. (2023). Mitochondrial dysfunction in neurodegenerative disorders: Potential therapeutic application of mitochondrial transfer to central nervous system-residing cells. J. Transl. Med..

[200] Zamora R., Vodovotz Y., Billiar T.R. (2000). Inducible nitric oxide synthase and inflammatory diseases. Mol. Med..

[201] Justo A.F.O., Suemoto C.K. (2022). The modulation of neuroinflammation by inducible nitric oxide synthase. J. Cell Commun. Signal..

[202] Panieri E., Santoro M.M. (2015). ROS signaling and redox biology in endothelial cells. Cell. Mol. Life Sci..

[203] Wang B., Wang Y., Zhang J., Hu C., Jiang J., Li Y., Peng Z. (2023). ROS-induced lipid peroxidation modulates cell death outcome: Mechanisms behind apoptosis, autophagy, and ferroptosis. Arch. Toxicol..

[204] Lin M.T., Beal M.F. (2006). Mitochondrial dysfunction and oxidative stress in neurodegenerative diseases. Nature.

[205] Wang X., Wang W., Li L., Perry G., Lee H.G., Zhu X. (2014). Oxidative stress and mitochondrial dysfunction in Alzheimer’s disease. Biochim. Biophys. Acta.

[206] Ebadpour N., Mahmoudi M., Kamal Kheder R., Abavisani M., Baridjavadi Z., Abdollahi N., Esmaeili S.A. (2024). From mitochondrial dysfunction to neuroinflammation in Parkinson’s disease: Pathogenesis and mitochondrial therapeutic approaches. Int. Immunopharmacol..

[207] Tang X., Luo Y.X., Chen H.Z., Liu D.P. (2014). Mitochondria, endothelial cell function, and vascular diseases. Front. Physiol..

[208] Li X., Fang P., Li Y., Kuo Y.M., Andrews A.J., Nanayakkara G., Johnson C., Fu H., Shan H., Du F. (2016). Mitochondrial Reactive Oxygen Species Mediate Lysophosphatidylcholine-Induced Endothelial Cell Activation. Arterioscler. Thromb. Vasc. Biol..

[209] Minjares M., Wu W., Wang J.M. (2023). Oxidative Stress and MicroRNAs in Endothelial Cells under Metabolic Disorders. Cells.

[210] Abdul-Muneer P.M., Chandra N., Haorah J. (2015). Interactions of oxidative stress and neurovascular inflammation in the pathogenesis of traumatic brain injury. Mol. Neurobiol..

[211] Rao R. (2008). Oxidative stress-induced disruption of epithelial and endothelial tight junctions. Front. Biosci..

[212] Sultana R., Butterfield D.A. (2024). Protein Oxidation in Aging and Alzheimer’s Disease Brain. Antioxidants.

[213] Haorah J., Ramirez S.H., Schall K., Smith D., Pandya R., Persidsky Y. (2007). Oxidative stress activates protein tyrosine kinase and matrix metalloproteinases leading to blood-brain barrier dysfunction. J. Neurochem..

[214] Alge-Priglinger C.S., Kreutzer T., Obholzer K., Wolf A., Mempel M., Kernt M., Kampik A., Priglinger S.G. (2009). Oxidative stress-mediated induction of MMP-1 and MMP-3 in human RPE cells. Investig. Ophthalmol. Vis. Sci..

[215] Xu R., Li Q., Zhou X.D., Perelman J.M., Kolosov V.P. (2013). Oxidative stress mediates the disruption of airway epithelial tight junctions through a TRPM2-PLCgamma1-PKCalpha signaling pathway. Int. J. Mol. Sci..

[216] Jiao H., Wang Z., Liu Y., Wang P., Xue Y. (2011). Specific role of tight junction proteins claudin-5, occludin, and ZO-1 of the blood-brain barrier in a focal cerebral ischemic insult. J. Mol. Neurosci..

[217] Yang Y., Rosenberg G.A. (2011). MMP-mediated disruption of claudin-5 in the blood-brain barrier of rat brain after cerebral ischemia. Methods Mol. Biol..

[218] Lee J.Y., Choi H.Y., Ahn H.J., Ju B.G., Yune T.Y. (2014). Matrix metalloproteinase-3 promotes early blood-spinal cord barrier disruption and hemorrhage and impairs long-term neurological recovery after spinal cord injury. Am. J. Pathol..

[219] Liu L., Yang C., Lavayen B.P., Tishko R.J., Larochelle J., Candelario-Jalil E. (2022). Targeted BRD4 protein degradation by dBET1 ameliorates acute ischemic brain injury and improves functional outcomes associated with reduced neuroinflammation and oxidative stress and preservation of blood-brain barrier integrity. J. Neuroinflamm..

[220] Dean T., Mendiola A.S., Yan Z., Meza-Acevedo R., Cabriga B., Akassoglou K., Ryu J.K. (2024). Fibrin promotes oxidative stress and neuronal loss in traumatic brain injury via innate immune activation. J. Neuroinflamm..

[221] Kempe S., Kestler H., Lasar A., Wirth T. (2005). NF-kappaB controls the global pro-inflammatory response in endothelial cells: Evidence for the regulation of a pro-atherogenic program. Nucleic Acids Res..

[222] Ashique S., Mishra N., Mantry S., Garg A., Kumar N., Gupta M., Kar S.K., Islam A., Mohanto S., Subramaniyan V. (2024). Crosstalk between ROS-inflammatory gene expression axis in the progression of lung disorders. Naunyn-Schmiedeberg’s Arch. Pharmacol..

[223] Manoharan R.R., Prasad A., Pospisil P., Kzhyshkowska J. (2024). ROS signaling in innate immunity via oxidative protein modifications. Front. Immunol..

[224] Yang J., Ran M., Li H., Lin Y., Ma K., Yang Y., Fu X., Yang S. (2022). New insight into neurological degeneration: Inflammatory cytokines and blood-brain barrier. Front. Mol. Neurosci..

[225] Lu W., Wen J. (2024). Crosstalk Among Glial Cells in the Blood-Brain Barrier Injury After Ischemic Stroke. Mol. Neurobiol..

[226] Steiner O., Coisne C., Cecchelli R., Boscacci R., Deutsch U., Engelhardt B., Lyck R. (2010). Differential roles for endothelial ICAM-1, ICAM-2, and VCAM-1 in shear-resistant T cell arrest, polarization, and directed crawling on blood-brain barrier endothelium. J. Immunol..

[227] Higashi Y. (2022). Roles of Oxidative Stress and Inflammation in Vascular Endothelial Dysfunction-Related Disease. Antioxidants.

[228] Fang X., Zhang Y., Zhang Y., Guan H., Huang X., Miao R., Yin R., Tian J. (2024). Endothelial extracellular vesicles: Their possible function and clinical significance in diabetic vascular complications. J. Transl. Med..

[229] Velmurugan G.V., Vekaria H.J., Hartz A.M.S., Bauer B., Hubbard W.B. (2024). Oxidative stress alters mitochondrial homeostasis in isolated brain capillaries. Fluids Barriers CNS.

[230] Ahmad W., Ijaz B., Shabbiri K., Ahmed F., Rehman S. (2017). Oxidative toxicity in diabetes and Alzheimer’s disease: Mechanisms behind ROS/ RNS generation. J. Biomed. Sci..

[231] Banks W.A., Rhea E.M. (2021). The Blood-Brain Barrier, Oxidative Stress, and Insulin Resistance. Antioxidants.

[232] Geronimo-Olvera C., Massieu L. (2019). Autophagy as a Homeostatic Mechanism in Response to Stress Conditions in the Central Nervous System. Mol. Neurobiol..

[233] Jadiya P., Garbincius J.F., Elrod J.W. (2021). Reappraisal of metabolic dysfunction in neurodegeneration: Focus on mitochondrial function and calcium signaling. Acta Neuropathol. Commun..

[234] Sita G., Hrelia P., Tarozzi A., Morroni F. (2017). P-glycoprotein (ABCB1) and Oxidative Stress: Focus on Alzheimer’s Disease. Oxidative Med. Cell. Longev..

[235] Amelimojarad M., Amelimojarad M., Cui X. (2024). The emerging role of brain neuroinflammatory responses in Alzheimer’s disease. Front. Aging Neurosci..

[236] da Fonseca A.C., Matias D., Garcia C., Amaral R., Geraldo L.H., Freitas C., Lima F.R. (2014). The impact of microglial activation on blood-brain barrier in brain diseases. Front. Cell. Neurosci..

[237] Liu L.R., Liu J.C., Bao J.S., Bai Q.Q., Wang G.Q. (2020). Interaction of Microglia and Astrocytes in the Neurovascular Unit. Front. Immunol..

[238] Kwon H.S., Koh S.H. (2020). Neuroinflammation in neurodegenerative disorders: The roles of microglia and astrocytes. Transl. Neurodegener..

[239] Woodburn S.C., Bollinger J.L., Wohleb E.S. (2021). The semantics of microglia activation: Neuroinflammation, homeostasis, and stress. J. Neuroinflamm..

[240] Singh D. (2022). Astrocytic and microglial cells as the modulators of neuroinflammation in Alzheimer’s disease. J. Neuroinflamm..

[241] Hansen D.V., Hanson J.E., Sheng M. (2018). Microglia in Alzheimer’s disease. J. Cell Biol..

[242] Shan C., Zhang C., Zhang C. (2024). The Role of IL-6 in Neurodegenerative Disorders. Neurochem. Res..

[243] O’Carroll S.J., Kho D.T., Wiltshire R., Nelson V., Rotimi O., Johnson R., Angel C.E., Graham E.S. (2015). Pro-inflammatory TNFalpha and IL-1beta differentially regulate the inflammatory phenotype of brain microvascular endothelial cells. J. Neuroinflamm..

[244] Guo X., Liu R., Jia M., Wang Q., Wu J. (2023). Ischemia Reperfusion Injury Induced Blood Brain Barrier Dysfunction and the Involved Molecular Mechanism. Neurochem. Res..

[245] Modur V., Zimmerman G.A., Prescott S.M., McIntyre T.M. (1996). Endothelial cell inflammatory responses to tumor necrosis factor alpha. Ceramide-dependent and -independent mitogen-activated protein kinase cascades. J. Biol. Chem..

[246] Zhang F., Yu W., Hargrove J.L., Greenspan P., Dean R.G., Taylor E.W., Hartle D.K. (2002). Inhibition of TNF-alpha induced ICAM-1, VCAM-1 and E-selectin expression by selenium. Atherosclerosis.

[247] Lin C.C., Pan C.S., Wang C.Y., Liu S.W., Hsiao L.D., Yang C.M. (2015). Tumor necrosis factor-alpha induces VCAM-1-mediated inflammation via c-Src-dependent transactivation of EGF receptors in human cardiac fibroblasts. J. Biomed. Sci..

[248] Majerova P., Michalicova A., Cente M., Hanes J., Vegh J., Kittel A., Kosikova N., Cigankova V., Mihaljevic S., Jadhav S. (2019). Trafficking of immune cells across the blood-brain barrier is modulated by neurofibrillary pathology in tauopathies. PLoS ONE.

[249] Nishihara H., Perriot S., Gastfriend B.D., Steinfort M., Cibien C., Soldati S., Matsuo K., Guimbal S., Mathias A., Palecek S.P. (2022). Intrinsic blood-brain barrier dysfunction contributes to multiple sclerosis pathogenesis. Brain.

[250] Forster C. (2008). Tight junctions and the modulation of barrier function in disease. Histochem. Cell Biol..

[251] Bhat A.A., Uppada S., Achkar I.W., Hashem S., Yadav S.K., Shanmugakonar M., Al-Naemi H.A., Haris M., Uddin S. (2018). Tight Junction Proteins and Signaling Pathways in Cancer and Inflammation: A Functional Crosstalk. Front. Physiol..

[252] Horowitz A., Chanez-Paredes S.D., Haest X., Turner J.R. (2023). Paracellular permeability and tight junction regulation in gut health and disease. Nat. Rev. Gastroenterol. Hepatol..

[253] Huang X., Wei P., Fang C., Yu M., Yang S., Qiu L., Wang Y., Xu A., Hoo R.L.C., Chang J. (2024). Compromised endothelial Wnt/beta-catenin signaling mediates the blood-brain barrier disruption and leads to neuroinflammation in endotoxemia. J. Neuroinflamm..

[254] Varghese S.M., Patel S., Nandan A., Jose A., Ghosh S., Sah R.K., Menon B., V A.K., Chakravarty S. (2024). Unraveling the Role of the Blood-Brain Barrier in the Pathophysiology of Depression: Recent Advances and Future Perspectives. Mol. Neurobiol..

[255] Versele R., Sevin E., Gosselet F., Fenart L., Candela P. (2022). TNF-alpha and IL-1beta Modulate Blood-Brain Barrier Permeability and Decrease Amyloid-beta Peptide Efflux in a Human Blood-Brain Barrier Model. Int. J. Mol. Sci..

[256] Nehme Z., Roehlen N., Dhawan P., Baumert T.F. (2023). Tight Junction Protein Signaling and Cancer Biology. Cells.

[257] He J., Qin M., Chen Y., Hu Z., Xie F., Ye L., Hui T. (2020). Epigenetic regulation of matrix metalloproteinases in inflammatory diseases: A narrative review. Cell Biosci..

[258] Lee H.S., Kim W.J. (2022). The Role of Matrix Metalloproteinase in Inflammation with a Focus on Infectious Diseases. Int. J. Mol. Sci..

[259] Feng S., Cen J., Huang Y., Shen H., Yao L., Wang Y., Chen Z. (2011). Matrix metalloproteinase-2 and -9 secreted by leukemic cells increase the permeability of blood-brain barrier by disrupting tight junction proteins. PLoS ONE.

[260] Almutairi S., Kalloush H.M., Manoon N.A., Bardaweel S.K. (2023). Matrix Metalloproteinases Inhibitors in Cancer Treatment: An Updated Review (2013–2023). Molecules.

[261] Rosenberg G.A. (2009). Matrix metalloproteinases and their multiple roles in neurodegenerative diseases. Lancet Neurol..

[262] Candelario-Jalil E., Dijkhuizen R.M., Magnus T. (2022). Neuroinflammation, Stroke, Blood-Brain Barrier Dysfunction, and Imaging Modalities. Stroke.

[263] Lawson C., Wolf S. (2009). ICAM-1 signaling in endothelial cells. Pharmacol. Rep..

[264] Pinte S., Caetano B., Le Bras A., Havet C., Villain G., Dernayka R., Duez C., Mattot V., Soncin F. (2016). Endothelial Cell Activation Is Regulated by Epidermal Growth Factor-like Domain 7 (Egfl7) during Inflammation. J. Biol. Chem..

[265] Birch C.A., Wedegaertner H., Orduna-Castillo L.B., Gonzalez Ramirez M.L., Qin H., Trejo J. (2023). Endothelial APC/PAR1 distinctly regulates cytokine-induced pro-inflammatory VCAM-1 expression. Front. Mol. Biosci..

[266] Engelhardt B. (2006). Molecular mechanisms involved in T cell migration across the blood-brain barrier. J. Neural Transm..

[267] Gonzalez H., Pacheco R. (2014). T-cell-mediated regulation of neuroinflammation involved in neurodegenerative diseases. J. Neuroinflamm..

[268] Sevenich L. (2018). Brain-Resident Microglia and Blood-Borne Macrophages Orchestrate Central Nervous System Inflammation in Neurodegenerative Disorders and Brain Cancer. Front. Immunol..

[269] Vandenbark A.A., Offner H., Matejuk S., Matejuk A. (2021). Microglia and astrocyte involvement in neurodegeneration and brain cancer. J. Neuroinflamm..

[270] Zhu G., Wang X., Chen L., Lenahan C., Fu Z., Fang Y., Yu W. (2022). Crosstalk Between the Oxidative Stress and Glia Cells After Stroke: From Mechanism to Therapies. Front. Immunol..

[271] Mayer M.G., Fischer T. (2024). Microglia at the blood brain barrier in health and disease. Front. Cell. Neurosci..

[272] Lawrence J.M., Schardien K., Wigdahl B., Nonnemacher M.R. (2023). Roles of neuropathology-associated reactive astrocytes: A systematic review. Acta Neuropathol. Commun..

[273] Mora P., Laisne M., Bourguignon C., Rouault P., Jaspard-Vinassa B., Maitre M., Gadeau A.P., Renault M.A., Horng S., Couffinhal T. (2024). Astrocytic DLL4-NOTCH1 signaling pathway promotes neuroinflammation via the IL-6-STAT3 axis. J. Neuroinflamm..

[274] Yu N., Cui H., Jin S., Liu P., Fang Y., Sun F., Cao Y., Yuan B., Xie Y., Duan W. (2024). IL-6 from cerebrospinal fluid causes widespread pain via STAT3-mediated astrocytosis in chronic constriction injury of the infraorbital nerve. J. Neuroinflamm..

[275] Burkhart A., Helgudottir S.S., Mahamed Y.A., Fruergaard M.B., Holm-Jacobsen J.N., Haraldsdottir H., Dahl S.E., Pretzmann F., Routhe L.G., Lambertsen K. (2024). Activation of glial cells induces proinflammatory properties in brain capillary endothelial cells in vitro. Sci. Rep..

[276] Corps K.N., Roth T.L., McGavern D.B. (2015). Inflammation and neuroprotection in traumatic brain injury. JAMA Neurol..

[277] Bodnar C.N., Watson J.B., Higgins E.K., Quan N., Bachstetter A.D. (2021). Inflammatory Regulation of CNS Barriers After Traumatic Brain Injury: A Tale Directed by Interleukin-1. Front. Immunol..

[278] Mokbel A.Y., Burns M.P., Main B.S. (2024). The contribution of the meningeal immune interface to neuroinflammation in traumatic brain injury. J. Neuroinflamm..

[279] Obukohwo O.M., Oreoluwa O.A., Andrew U.O., Williams U.E. (2024). Microglia-mediated neuroinflammation in traumatic brain injury: A review. Mol. Biol. Rep..

[280] Mayne K., White J.A., McMurran C.E., Rivera F.J., de la Fuente A.G. (2020). Aging and Neurodegenerative Disease: Is the Adaptive Immune System a Friend or Foe?. Front. Aging Neurosci..

[281] Michalicova A., Majerova P., Kovac A. (2020). Tau Protein and Its Role in Blood-Brain Barrier Dysfunction. Front. Mol. Neurosci..

[282] Moussaud S., Jones D.R., Moussaud-Lamodiere E.L., Delenclos M., Ross O.A., McLean P.J. (2014). Alpha-synuclein and tau: Teammates in neurodegeneration?. Mol. Neurodegener..

[283] Sun X., Chen W.D., Wang Y.D. (2015). beta-Amyloid: The key peptide in the pathogenesis of Alzheimer’s disease. Front. Pharmacol..

[284] Pan L., Meng L., He M., Zhang Z. (2021). Tau in the Pathophysiology of Parkinson’s Disease. J. Mol. Neurosci..

[285] Bogale T.A., Faustini G., Longhena F., Mitola S., Pizzi M., Bellucci A. (2021). Alpha-Synuclein in the Regulation of Brain Endothelial and Perivascular Cells: Gaps and Future Perspectives. Front. Immunol..

[286] Paik S., Somvanshi R.K., Kumar U. (2019). Somatostatin Maintains Permeability and Integrity of Blood-Brain Barrier in beta-Amyloid Induced Toxicity. Mol. Neurobiol..

[287] Wang D., Chen F., Han Z., Yin Z., Ge X., Lei P. (2021). Relationship Between Amyloid-beta Deposition and Blood-Brain Barrier Dysfunction in Alzheimer’s Disease. Front. Cell. Neurosci..

[288] Liu W.Y., Wang Z.B., Zhang L.C., Wei X., Li L. (2012). Tight junction in blood-brain barrier: An overview of structure, regulation, and regulator substances. CNS Neurosci. Ther..

[289] Bernardo-Castro S., Sousa J.A., Bras A., Cecilia C., Rodrigues B., Almendra L., Machado C., Santo G., Silva F., Ferreira L. (2020). Pathophysiology of Blood-Brain Barrier Permeability Throughout the Different Stages of Ischemic Stroke and Its Implication on Hemorrhagic Transformation and Recovery. Front. Neurol..

[290] Cuevas E., Rosas-Hernandez H., Burks S.M., Ramirez-Lee M.A., Guzman A., Imam S.Z., Ali S.F., Sarkar S. (2019). Amyloid Beta 25-35 induces blood-brain barrier disruption in vitro. Metab. Brain Dis..

[291] Cai Z., Zhao B., Ratka A. (2011). Oxidative stress and beta-amyloid protein in Alzheimer’s disease. Neuromolecular Med..

[292] Fanlo-Ucar H., Picon-Pages P., Herrera-Fernandez V., Ill-Raga G., Munoz F.J. (2024). The Dual Role of Amyloid Beta-Peptide in Oxidative Stress and Inflammation: Unveiling Their Connections in Alzheimer’s Disease Etiopathology. Antioxidants.

[293] Doens D., Fernandez P.L. (2014). Microglia receptors and their implications in the response to amyloid beta for Alzheimer’s disease pathogenesis. J. Neuroinflamm..

[294] Hussain B., Fang C., Chang J. (2021). Blood-Brain Barrier Breakdown: An Emerging Biomarker of Cognitive Impairment in Normal Aging and Dementia. Front. Neurosci..

[295] Kanekiyo T., Bu G. (2014). The low-density lipoprotein receptor-related protein 1 and amyloid-beta clearance in Alzheimer’s disease. Front. Aging Neurosci..

[296] Ramanathan A., Nelson A.R., Sagare A.P., Zlokovic B.V. (2015). Impaired vascular-mediated clearance of brain amyloid beta in Alzheimer’s disease: The role, regulation and restoration of LRP1. Front. Aging Neurosci..

[297] Storck S.E., Meister S., Nahrath J., Meissner J.N., Schubert N., Di Spiezio A., Baches S., Vandenbroucke R.E., Bouter Y., Prikulis I. (2016). Endothelial LRP1 transports amyloid-beta(1-42) across the blood-brain barrier. J. Clin. Investig..

[298] Kanekiyo T., Liu C.C., Shinohara M., Li J., Bu G. (2012). LRP1 in brain vascular smooth muscle cells mediates local clearance of Alzheimer’s amyloid-beta. J. Neurosci..

[299] Daniele S., Pietrobono D., Fusi J., Iofrida C., Chico L., Petrozzi L., Gerfo A.L., Baldacci F., Galetta F., Siciliano G. (2018). alpha-Synuclein Aggregates with beta-Amyloid or Tau in Human Red Blood Cells: Correlation with Antioxidant Capability and Physical Exercise in Human Healthy Subjects. Mol. Neurobiol..

[300] Houldsworth A. (2024). Role of oxidative stress in neurodegenerative disorders: A review of reactive oxygen species and prevention by antioxidants. Brain Commun..

[301] Wang Y., Kuca K., You L., Nepovimova E., Heger Z., Valko M., Adam V., Wu Q., Jomova K. (2024). The role of cellular senescence in neurodegenerative diseases. Arch. Toxicol..

[302] Wu L., Xiong X., Wu X., Ye Y., Jian Z., Zhi Z., Gu L. (2020). Targeting Oxidative Stress and Inflammation to Prevent Ischemia-Reperfusion Injury. Front. Mol. Neurosci..

[303] Zhao Y., Gong C.X. (2015). From chronic cerebral hypoperfusion to Alzheimer-like brain pathology and neurodegeneration. Cell. Mol. Neurobiol..

[304] Dong S., Maniar S., Manole M.D., Sun D. (2018). Cerebral Hypoperfusion and Other Shared Brain Pathologies in Ischemic Stroke and Alzheimer’s Disease. Transl. Stroke Res..

[305] Rajeev V., Chai Y.L., Poh L., Selvaraji S., Fann D.Y., Jo D.G., De Silva T.M., Drummond G.R., Sobey C.G., Arumugam T.V. (2023). Chronic cerebral hypoperfusion: A critical feature in unravelling the etiology of vascular cognitive impairment. Acta Neuropathol. Commun..

[306] Xu W., Bai Q., Dong Q., Guo M., Cui M. (2022). Blood-Brain Barrier Dysfunction and the Potential Mechanisms in Chronic Cerebral Hypoperfusion Induced Cognitive Impairment. Front. Cell. Neurosci..

[307] Page S., Raut S., Al-Ahmad A. (2019). Oxygen-Glucose Deprivation/Reoxygenation-Induced Barrier Disruption at the Human Blood-Brain Barrier is Partially Mediated Through the HIF-1 Pathway. Neuromolecular Med..

[308] Luo Z., Yao J., Wang Z., Xu J. (2023). Mitochondria in endothelial cells angiogenesis and function: Current understanding and future perspectives. J. Transl. Med..

[309] Pang B., Dong G., Pang T., Sun X., Liu X., Nie Y., Chang X. (2024). Emerging insights into the pathogenesis and therapeutic strategies for vascular endothelial injury-associated diseases: Focus on mitochondrial dysfunction. Angiogenesis.

[310] Yang L., Song J., Nan D., Wan Y., Guo H. (2022). Cognitive Impairments and blood-brain Barrier Damage in a Mouse Model of Chronic Cerebral Hypoperfusion. Neurochem. Res..

[311] Lochhead J.J., McCaffrey G., Quigley C.E., Finch J., DeMarco K.M., Nametz N., Davis T.P. (2010). Oxidative stress increases blood-brain barrier permeability and induces alterations in occludin during hypoxia-reoxygenation. J. Cereb. Blood Flow Metab..

[312] Dunn J.F., Isaacs A.M. (2021). The impact of hypoxia on blood-brain, blood-CSF, and CSF-brain barriers. J. Appl. Physiol. (1985).

[313] Zedde M., Pascarella R. (2024). The Cerebrovascular Side of Plasticity: Microvascular Architecture across Health and Neurodegenerative and Vascular Diseases. Brain Sci..

[314] Sladojevic N., Stamatovic S.M., Johnson A.M., Choi J., Hu A., Dithmer S., Blasig I.E., Keep R.F., Andjelkovic A.V. (2019). Claudin-1-Dependent Destabilization of the Blood-Brain Barrier in Chronic Stroke. J. Neurosci..

[315] Freitas-Andrade M., Raman-Nair J., Lacoste B. (2020). Structural and Functional Remodeling of the Brain Vasculature Following Stroke. Front. Physiol..

[316] Halder S.K., Delorme-Walker V.D., Milner R. (2023). beta1 integrin is essential for blood-brain barrier integrity under stable and vascular remodelling conditions; effects differ with age. Fluids Barriers CNS.

[317] Yao Y. (2019). Basement membrane and stroke. J. Cereb. Blood Flow Metab..

[318] Monaci S., Coppola F., Filippi I., Falsini A., Carraro F., Naldini A. (2024). Targeting hypoxia signaling pathways in angiogenesis. Front. Physiol..

[319] Zhao Y., Yu B., Wang Y., Tan S., Xu Q., Wang Z., Zhou K., Liu H., Ren Z., Jiang Z. (2024). Ang-1 and VEGF: Central regulators of angiogenesis. Mol. Cell. Biochem..

[320] Bauer H.C., Krizbai I.A., Bauer H., Traweger A. (2014). “You Shall Not Pass”-tight junctions of the blood brain barrier. Front. Neurosci..

[321] Grammas P. (2011). Neurovascular dysfunction, inflammation and endothelial activation: Implications for the pathogenesis of Alzheimer’s disease. J. Neuroinflamm..

[322] Carmeliet P., Ruiz de Almodovar C. (2013). VEGF ligands and receptors: Implications in neurodevelopment and neurodegeneration. Cell. Mol. Life Sci..

[323] Shim J.W., Madsen J.R. (2018). VEGF Signaling in Neurological Disorders. Int. J. Mol. Sci..

[324] Zhang Z.G., Zhang L., Jiang Q., Zhang R., Davies K., Powers C., Bruggen N., Chopp M. (2000). VEGF enhances angiogenesis and promotes blood-brain barrier leakage in the ischemic brain. J. Clin. Investig..

[325] Hu Y., Zheng Y., Wang T., Jiao L., Luo Y. (2022). VEGF, a Key Factor for Blood Brain Barrier Injury After Cerebral Ischemic Stroke. Aging Dis..

[326] Wu M., Gong Y., Jiang L., Zhang M., Gu H., Shen H., Dang B. (2022). VEGF regulates the blood-brain barrier through MMP-9 in a rat model of traumatic brain injury. Exp. Ther. Med..

[327] Hashimoto Y., Campbell M., Tachibana K., Okada Y., Kondoh M. (2021). Claudin-5: A Pharmacological Target to Modify the Permeability of the Blood-Brain Barrier. Biol. Pharm. Bull..

[328] Dithmer S., Blasig I.E., Fraser P.A., Qin Z., Haseloff R.F. (2024). The Basic Requirement of Tight Junction Proteins in Blood-Brain Barrier Function and Their Role in Pathologies. Int. J. Mol. Sci..

[329] Antonetti D.A., Barber A.J., Hollinger L.A., Wolpert E.B., Gardner T.W. (1999). Vascular endothelial growth factor induces rapid phosphorylation of tight junction proteins occludin and zonula occluden 1. A potential mechanism for vascular permeability in diabetic retinopathy and tumors. J. Biol. Chem..

[330] Bates D.O. (2010). Vascular endothelial growth factors and vascular permeability. Cardiovasc. Res..

[331] Bai Y., Zhu X., Chao J., Zhang Y., Qian C., Li P., Liu D., Han B., Zhao L., Zhang J. (2015). Pericytes contribute to the disruption of the cerebral endothelial barrier via increasing VEGF expression: Implications for stroke. PLoS ONE.

[332] Uemura M.T., Maki T., Ihara M., Lee V.M.Y., Trojanowski J.Q. (2020). Brain Microvascular Pericytes in Vascular Cognitive Impairment and Dementia. Front. Aging Neurosci..

[333] Li P., Fan H. (2023). Pericyte Loss in Diseases. Cells.

[334] Sun Z., Gao C., Gao D., Sun R., Li W., Wang F., Wang Y., Cao H., Zhou G., Zhang J. (2021). Reduction in pericyte coverage leads to blood-brain barrier dysfunction via endothelial transcytosis following chronic cerebral hypoperfusion. Fluids Barriers CNS.

[335] Sengillo J.D., Winkler E.A., Walker C.T., Sullivan J.S., Johnson M., Zlokovic B.V. (2013). Deficiency in mural vascular cells coincides with blood-brain barrier disruption in Alzheimer’s disease. Brain Pathol..

[336] Shi H., Koronyo Y., Rentsendorj A., Regis G.C., Sheyn J., Fuchs D.T., Kramerov A.A., Ljubimov A.V., Dumitrascu O.M., Rodriguez A.R. (2020). Identification of early pericyte loss and vascular amyloidosis in Alzheimer’s disease retina. Acta Neuropathol..

[337] Hamilton N.B., Attwell D., Hall C.N. (2010). Pericyte-mediated regulation of capillary diameter: A component of neurovascular coupling in health and disease. Front. Neuroenergetics.

[338] Hall C.N., Reynell C., Gesslein B., Hamilton N.B., Mishra A., Sutherland B.A., O’Farrell F.M., Buchan A.M., Lauritzen M., Attwell D. (2014). Capillary pericytes regulate cerebral blood flow in health and disease. Nature.

[339] Burek M., Konig A., Lang M., Fiedler J., Oerter S., Roewer N., Bohnert M., Thal S.C., Blecharz-Lang K.G., Woitzik J. (2019). Hypoxia-Induced MicroRNA-212/132 Alter Blood-Brain Barrier Integrity Through Inhibition of Tight Junction-Associated Proteins in Human and Mouse Brain Microvascular Endothelial Cells. Transl. Stroke Res..

[340] Ozgur B., Helms H.C.C., Tornabene E., Brodin B. (2022). Hypoxia increases expression of selected blood-brain barrier transporters GLUT-1, P-gp, SLC7A5 and TFRC, while maintaining barrier integrity, in brain capillary endothelial monolayers. Fluids Barriers CNS.

[341] Agafonova A., Cosentino A., Musso N., Prinzi C., Russo C., Pellitteri R., Anfuso C.D., Lupo G. (2024). Hypoxia-Induced Inflammation in In Vitro Model of Human Blood-Brain Barrier: Modulatory Effects of the Olfactory Ensheathing Cell-Conditioned Medium. Mol. Neurobiol..

[342] Jones A.R., Shusta E.V. (2007). Blood-brain barrier transport of therapeutics via receptor-mediation. Pharm. Res..

[343] Ohtsuki S., Terasaki T. (2007). Contribution of carrier-mediated transport systems to the blood-brain barrier as a supporting and protecting interface for the brain; importance for CNS drug discovery and development. Pharm. Res..

[344] Pulgar V.M. (2018). Transcytosis to Cross the Blood Brain Barrier, New Advancements and Challenges. Front. Neurosci..

[345] Winkler E.A., Nishida Y., Sagare A.P., Rege S.V., Bell R.D., Perlmutter D., Sengillo J.D., Hillman S., Kong P., Nelson A.R. (2015). GLUT1 reductions exacerbate Alzheimer’s disease vasculo-neuronal dysfunction and degeneration. Nat. Neurosci..

[346] Kyrtata N., Emsley H.C.A., Sparasci O., Parkes L.M., Dickie B.R. (2021). A Systematic Review of Glucose Transport Alterations in Alzheimer’s Disease. Front. Neurosci..

[347] Chen Y., Joo J., Chu J.M., Chang R.C., Wong G.T. (2023). Downregulation of the glucose transporter GLUT 1 in the cerebral microvasculature contributes to postoperative neurocognitive disorders in aged mice. J. Neuroinflamm..

[348] Szablewski L. (2021). Brain Glucose Transporters: Role in Pathogenesis and Potential Targets for the Treatment of Alzheimer’s Disease. Int. J. Mol. Sci..

[349] Zhang J., Xu Y., Li D., Fu L., Zhang X., Bao Y., Zheng L. (2020). Review of the Correlation of LAT1 with Diseases: Mechanism and Treatment. Front. Chem..

[350] Puris E., Saveleva L., de Sousa Maciel I., Kanninen K.M., Auriola S., Fricker G. (2023). Protein Expression of Amino Acid Transporters Is Altered in Isolated Cerebral Microvessels of 5xFAD Mouse Model of Alzheimer’s Disease. Mol. Neurobiol..

[351] Yousefzadeh S.A., Jarah M., Riazi G.H. (2020). Tryptophan Improves Memory Independent of Its Role as a Serotonin Precursor: Potential Involvement of Microtubule Proteins. J. Mol. Neurosci..

[352] Roth W., Zadeh K., Vekariya R., Ge Y., Mohamadzadeh M. (2021). Tryptophan Metabolism and Gut-Brain Homeostasis. Int. J. Mol. Sci..

[353] Rajagopal S., Sangam S.R., Singh S., Joginapally V.R. (2016). Modulatory Effects of Dietary Amino Acids on Neurodegenerative Diseases. Adv. Neurobiol..

[354] Marx W., McGuinness A.J., Rocks T., Ruusunen A., Cleminson J., Walker A.J., Gomes-da-Costa S., Lane M., Sanches M., Diaz A.P. (2021). The kynurenine pathway in major depressive disorder, bipolar disorder, and schizophrenia: A meta-analysis of 101 studies. Mol. Psychiatry.

[355] Sharma V.K., Singh T.G., Prabhakar N.K., Mannan A. (2022). Kynurenine Metabolism and Alzheimer’s Disease: The Potential Targets and Approaches. Neurochem. Res..

[356] Malik B.R., Maddison D.C., Smith G.A., Peters O.M. (2019). Autophagic and endo-lysosomal dysfunction in neurodegenerative disease. Mol. Brain.

[357] Lewitt M.S., Boyd G.W. (2019). The Role of Insulin-Like Growth Factors and Insulin-Like Growth Factor-Binding Proteins in the Nervous System. Biochem. Insights.

[358] Rhea E.M., Banks W.A. (2019). Role of the Blood-Brain Barrier in Central Nervous System Insulin Resistance. Front. Neurosci..

[359] Miao J., Zhang Y., Su C., Zheng Q., Guo J. (2024). Insulin-Like Growth Factor Signaling in Alzheimer’s Disease: Pathophysiology and Therapeutic Strategies. Mol. Neurobiol..

[360] Kleinridders A., Ferris H.A., Cai W., Kahn C.R. (2014). Insulin action in brain regulates systemic metabolism and brain function. Diabetes.

[361] Gray S.M., Aylor K.W., Barrett E.J. (2017). Unravelling the regulation of insulin transport across the brain endothelial cell. Diabetologia.

[362] Hersom M., Helms H.C., Schmalz C., Pedersen T.A., Buckley S.T., Brodin B. (2018). The insulin receptor is expressed and functional in cultured blood-brain barrier endothelial cells but does not mediate insulin entry from blood to brain. Am. J. Physiol. Endocrinol. Metab..

[363] Talbot K., Wang H.Y., Kazi H., Han L.Y., Bakshi K.P., Stucky A., Fuino R.L., Kawaguchi K.R., Samoyedny A.J., Wilson R.S. (2012). Demonstrated brain insulin resistance in Alzheimer’s disease patients is associated with IGF-1 resistance, IRS-1 dysregulation, and cognitive decline. J. Clin. Investig..

[364] Diehl T., Mullins R., Kapogiannis D. (2017). Insulin resistance in Alzheimer’s disease. Transl. Res..

[365] Benedict C., Grillo C.A. (2018). Insulin Resistance as a Therapeutic Target in the Treatment of Alzheimer’s Disease: A State-of-the-Art Review. Front. Neurosci..

[366] Spinelli M., Fusco S., Grassi C. (2019). Brain Insulin Resistance and Hippocampal Plasticity: Mechanisms and Biomarkers of Cognitive Decline. Front. Neurosci..

[367] Moos T., Morgan E.H. (2000). Transferrin and transferrin receptor function in brain barrier systems. Cell. Mol. Neurobiol..

[368] Crichton R.R., Dexter D.T., Ward R.J. (2011). Brain iron metabolism and its perturbation in neurological diseases. J. Neural Transm..

[369] Skjorringe T., Burkhart A., Johnsen K.B., Moos T. (2015). Divalent metal transporter 1 (DMT1) in the brain: Implications for a role in iron transport at the blood-brain barrier, and neuronal and glial pathology. Front. Mol. Neurosci..

[370] Li K., Reichmann H. (2016). Role of iron in neurodegenerative diseases. J. Neural Transm..

[371] Carocci A., Catalano A., Sinicropi M.S., Genchi G. (2018). Oxidative stress and neurodegeneration: The involvement of iron. Biometals.

[372] Gao G., You L., Zhang J., Chang Y.Z., Yu P. (2023). Brain Iron Metabolism, Redox Balance and Neurological Diseases. Antioxidants.

[373] Dutheil F., Dauchy S., Diry M., Sazdovitch V., Cloarec O., Mellottee L., Bieche I., Ingelman-Sundberg M., Flinois J.P., de Waziers I. (2009). Xenobiotic-metabolizing enzymes and transporters in the normal human brain: Regional and cellular mapping as a basis for putative roles in cerebral function. Drug Metab. Dispos..

[374] Balasubramanian A., Sundrud M.S. (2023). ATP-dependent transporters: Emerging players at the crossroads of immunity and metabolism. Front. Immunol..

[375] Fang Y.C., Hsieh Y.C., Hu C.J., Tu Y.K. (2023). Endothelial Dysfunction in Neurodegenerative Diseases. Int. J. Mol. Sci..

[376] Chai A.B., Leung G.K.F., Callaghan R., Gelissen I.C. (2020). P-glycoprotein: A role in the export of amyloid-beta in Alzheimer’s disease?. FEBS J..

[377] Ding Y., Zhong Y., Baldeshwiler A., Abner E.L., Bauer B., Hartz A.M.S. (2021). Protecting P-glycoprotein at the blood-brain barrier from degradation in an Alzheimer’s disease mouse model. Fluids Barriers CNS.

[378] Vulin M., Zhong Y., Maloney B.J., Bauer B., Hartz A.M.S. (2023). Proteasome inhibition protects blood-brain barrier P-glycoprotein and lowers Abeta brain levels in an Alzheimer’s disease model. Fluids Barriers CNS.

[379] Masato A., Plotegher N., Boassa D., Bubacco L. (2019). Impaired dopamine metabolism in Parkinson’s disease pathogenesis. Mol. Neurodegener..

[380] Zhou Z.D., Yi L.X., Wang D.Q., Lim T.M., Tan E.K. (2023). Role of dopamine in the pathophysiology of Parkinson’s disease. Transl. Neurodegener..

[381] Flores-Ponce X., Velasco I. (2024). Dopaminergic neuron metabolism: Relevance for understanding Parkinson’s disease. Metabolomics.

[382] Wong A.D., Ye M., Levy A.F., Rothstein J.D., Bergles D.E., Searson P.C. (2013). The blood-brain barrier: An engineering perspective. Front. Neuroeng..

[383] Hladky S.B., Barrand M.A. (2016). Fluid and ion transfer across the blood-brain and blood-cerebrospinal fluid barriers; a comparative account of mechanisms and roles. Fluids Barriers CNS.

[384] Marambaud P., Dreses-Werringloer U., Vingtdeux V. (2009). Calcium signaling in neurodegeneration. Mol. Neurodegener..

[385] Bastioli G., Piccirillo S., Graciotti L., Carone M., Sprega G., Taoussi O., Preziuso A., Castaldo P. (2024). Calcium Deregulation in Neurodegeneration and Neuroinflammation in Parkinson’s Disease: Role of Calcium-Storing Organelles and Sodium-Calcium Exchanger. Cells.

[386] Wang S., Wang B., Shang D., Zhang K., Yan X., Zhang X. (2022). Ion Channel Dysfunction in Astrocytes in Neurodegenerative Diseases. Front. Physiol..

[387] Orfali R., Alwatban A.Z., Orfali R.S., Lau L., Chea N., Alotaibi A.M., Nam Y.W., Zhang M. (2024). Oxidative stress and ion channels in neurodegenerative diseases. Front. Physiol..

[388] Clausen M.V., Hilbers F., Poulsen H. (2017). The Structure and Function of the Na,K-ATPase Isoforms in Health and Disease. Front. Physiol..

[389] Theophanous S., Sargiannidou I., Kleopa K.A. (2024). Glial Cells as Key Regulators in Neuroinflammatory Mechanisms Associated with Multiple Sclerosis. Int. J. Mol. Sci..

[390] Woo M.S., Engler J.B., Friese M.A. (2024). The neuropathobiology of multiple sclerosis. Nat. Rev. Neurosci..

[391] Frank-Cannon T.C., Alto L.T., McAlpine F.E., Tansey M.G. (2009). Does neuroinflammation fan the flame in neurodegenerative diseases?. Mol. Neurodegener..

[392] Schartz N.D., Tenner A.J. (2020). The good, the bad, and the opportunities of the complement system in neurodegenerative disease. J. Neuroinflamm..

[393] Zhao N., Francis N.L., Calvelli H.R., Moghe P.V. (2020). Microglia-targeting nanotherapeutics for neurodegenerative diseases. APL Bioeng..

[394] Qiu Y.M., Zhang C.L., Chen A.Q., Wang H.L., Zhou Y.F., Li Y.N., Hu B. (2021). Immune Cells in the BBB Disruption After Acute Ischemic Stroke: Targets for Immune Therapy?. Front. Immunol..

[395] Mapunda J.A., Tibar H., Regragui W., Engelhardt B. (2022). How Does the Immune System Enter the Brain?. Front. Immunol..

[396] Nair A.L., Groenendijk L., Overdevest R., Fowke T.M., Annida R., Mocellin O., de Vries H.E., Wevers N.R. (2023). Human BBB-on-a-chip reveals barrier disruption, endothelial inflammation, and T cell migration under neuroinflammatory conditions. Front. Mol. Neurosci..

[397] Stamp M.E.M., Halwes M., Nisbet D., Collins D.J. (2023). Breaking barriers: Exploring mechanisms behind opening the blood-brain barrier. Fluids Barriers CNS.

[398] Banks W.A., Gray A.M., Erickson M.A., Salameh T.S., Damodarasamy M., Sheibani N., Meabon J.S., Wing E.E., Morofuji Y., Cook D.G. (2015). Lipopolysaccharide-induced blood-brain barrier disruption: Roles of cyclooxygenase, oxidative stress, neuroinflammation, and elements of the neurovascular unit. J. Neuroinflamm..

[399] Kim Y., Lee S., Zhang H., Lee S., Kim H., Kim Y., Won M.H., Kim Y.M., Kwon Y.G. (2020). CLEC14A deficiency exacerbates neuronal loss by increasing blood-brain barrier permeability and inflammation. J. Neuroinflamm..

[400] Zang X., Chen S., Zhu J., Ma J., Zhai Y. (2022). The Emerging Role of Central and Peripheral Immune Systems in Neurodegenerative Diseases. Front. Aging Neurosci..

[401] Zhang Q., Yang G., Luo Y., Jiang L., Chi H., Tian G. (2024). Neuroinflammation in Alzheimer’s disease: Insights from peripheral immune cells. Immun. Ageing.

[402] Ao L.Y., Yan Y.Y., Zhou L., Li C.Y., Li W.T., Fang W.R., Li Y.M. (2018). Immune Cells After Ischemic Stroke Onset: Roles, Migration, and Target Intervention. J. Mol. Neurosci..

[403] Cekanaviciute E., Buckwalter M.S. (2016). Astrocytes: Integrative Regulators of Neuroinflammation in Stroke and Other Neurological Diseases. Neurotherapeutics.

[404] Thompson K.K., Tsirka S.E. (2017). The Diverse Roles of Microglia in the Neurodegenerative Aspects of Central Nervous System (CNS) Autoimmunity. Int. J. Mol. Sci..

[405] Bachiller S., Jimenez-Ferrer I., Paulus A., Yang Y., Swanberg M., Deierborg T., Boza-Serrano A. (2018). Microglia in Neurological Diseases: A Road Map to Brain-Disease Dependent-Inflammatory Response. Front. Cell. Neurosci..

[406] Solleiro-Villavicencio H., Rivas-Arancibia S. (2018). Effect of Chronic Oxidative Stress on Neuroinflammatory Response Mediated by CD4(+)T Cells in Neurodegenerative Diseases. Front. Cell. Neurosci..

[407] Teleanu D.M., Niculescu A.G., Lungu I.I., Radu C.I., Vladacenco O., Roza E., Costachescu B., Grumezescu A.M., Teleanu R.I. (2022). An Overview of Oxidative Stress, Neuroinflammation, and Neurodegenerative Diseases. Int. J. Mol. Sci..

[408] Kim S., Moon G.J., Kim H.J., Kim D.G., Kim J., Nam Y., Sharma C., Leem E., Lee S., Kim K.S. (2022). Control of hippocampal prothrombin kringle-2 (pKr-2) expression reduces neurotoxic symptoms in five familial Alzheimer’s disease mice. Br. J. Pharmacol..

[409] Che J., Sun Y., Deng Y., Zhang J. (2024). Blood-brain barrier disruption: A culprit of cognitive decline?. Fluids Barriers CNS.

[410] Iannucci J., Renehan W., Grammas P. (2020). Thrombin, a Mediator of Coagulation, Inflammation, and Neurotoxicity at the Neurovascular Interface: Implications for Alzheimer’s Disease. Front. Neurosci..

[411] Iannucci J., Grammas P. (2023). Thrombin, a Key Driver of Pathological Inflammation in the Brain. Cells.

[412] Kim S., Sharma C., Shin M., Kim H.J., Kim J., Kim S.R. (2023). pKr-2 induces neurodegeneration via upregulation of microglial TLR4 in the hippocampus of AD brain. Brain Behav. Immun. Health.

[413] Merlini M., Rafalski V.A., Rios Coronado P.E., Gill T.M., Ellisman M., Muthukumar G., Subramanian K.S., Ryu J.K., Syme C.A., Davalos D. (2019). Fibrinogen Induces Microglia-Mediated Spine Elimination and Cognitive Impairment in an Alzheimer’s Disease Model. Neuron.

[414] Cortes-Canteli M., Strickland S. (2009). Fibrinogen, a possible key player in Alzheimer’s disease. J. Thromb. Haemost..

[415] Fan D.Y., Sun H.L., Sun P.Y., Jian J.M., Li W.W., Shen Y.Y., Zeng F., Wang Y.J., Bu X.L. (2020). The Correlations Between Plasma Fibrinogen with Amyloid-Beta and Tau Levels in Patients with Alzheimer’s Disease. Front. Neurosci..

[416] Ralay Ranaivo H., Hodge J.N., Choi N., Wainwright M.S. (2012). Albumin induces upregulation of matrix metalloproteinase-9 in astrocytes via MAPK and reactive oxygen species-dependent pathways. J. Neuroinflamm..

[417] Weissberg I., Wood L., Kamintsky L., Vazquez O., Milikovsky D.Z., Alexander A., Oppenheim H., Ardizzone C., Becker A., Frigerio F. (2015). Albumin induces excitatory synaptogenesis through astrocytic TGF-beta/ALK5 signaling in a model of acquired epilepsy following blood-brain barrier dysfunction. Neurobiol. Dis..

[418] Preininger M.K., Zaytseva D., Lin J.M., Kaufer D. (2023). Blood-brain barrier dysfunction promotes astrocyte senescence through albumin-induced TGFbeta signaling activation. Aging Cell.

[419] Chen Y., He Y., Han J., Wei W., Chen F. (2023). Blood-brain barrier dysfunction and Alzheimer’s disease: Associations, pathogenic mechanisms, and therapeutic potential. Front. Aging Neurosci..

[420] Iqbal I., Saqib F., Mubarak Z., Latif M.F., Wahid M., Nasir B., Shahzad H., Sharifi-Rad J., Mubarak M.S. (2024). Alzheimer’s disease and drug delivery across the blood-brain barrier: Approaches and challenges. Eur. J. Med. Res..

[421] Wakabayashi K., Tanji K., Odagiri S., Miki Y., Mori F., Takahashi H. (2013). The Lewy body in Parkinson’s disease and related neurodegenerative disorders. Mol. Neurobiol..

[422] Hijaz B.A., Volpicelli-Daley L.A. (2020). Initiation and propagation of alpha-synuclein aggregation in the nervous system. Mol. Neurodegener..

[423] Cobos I., Palop J.J. (2022). Swollen axons impair neuronal circuits in Alzheimer’s disease. Nature.

[424] Sweeney P., Park H., Baumann M., Dunlop J., Frydman J., Kopito R., McCampbell A., Leblanc G., Venkateswaran A., Nurmi A. (2017). Protein misfolding in neurodegenerative diseases: Implications and strategies. Transl. Neurodegener..

[425] Pontano Vaites L., Harper J.W. (2018). Protein aggregates caught stalling. Nature.

[426] Giusti V., Kaur G., Giusto E., Civiero L. (2024). Brain clearance of protein aggregates: A close-up on astrocytes. Mol. Neurodegener..

[427] Koszla O., Solek P. (2024). Misfolding and aggregation in neurodegenerative diseases: Protein quality control machinery as potential therapeutic clearance pathways. Cell Commun. Signal..

[428] Wu Y., Ma B., Liu C., Li D., Sui G. (2024). Pathological Involvement of Protein Phase Separation and Aggregation in Neurodegenerative Diseases. Int. J. Mol. Sci..

[429] Chung C.G., Lee H., Lee S.B. (2018). Mechanisms of protein toxicity in neurodegenerative diseases. Cell. Mol. Life Sci..

[430] Bitetto G., Di Fonzo A. (2020). Nucleo-cytoplasmic transport defects and protein aggregates in neurodegeneration. Transl. Neurodegener..

[431] Wang X., Becker K., Levine N., Zhang M., Lieberman A.P., Moore D.J., Ma J. (2019). Pathogenic alpha-synuclein aggregates preferentially bind to mitochondria and affect cellular respiration. Acta Neuropathol. Commun..

[432] Duarte F.V., Ciampi D., Duarte C.B. (2023). Mitochondria as central hubs in synaptic modulation. Cell. Mol. Life Sci..

[433] Demuro A., Parker I., Stutzmann G.E. (2010). Calcium signaling and amyloid toxicity in Alzheimer disease. J. Biol. Chem..

[434] Stefanis L. (2012). alpha-Synuclein in Parkinson’s disease. Cold Spring Harb. Perspect. Med..

[435] Calabresi P., Di Lazzaro G., Marino G., Campanelli F., Ghiglieri V. (2023). Advances in understanding the function of alpha-synuclein: Implications for Parkinson’s disease. Brain.

[436] Simpson D.S.A., Oliver P.L. (2020). ROS Generation in Microglia: Understanding Oxidative Stress and Inflammation in Neurodegenerative Disease. Antioxidants.

[437] Firdous S.M., Khan S.A., Maity A. (2024). Oxidative stress-mediated neuroinflammation in Alzheimer’s disease. Naunyn-Schmiedeberg’s Arch. Pharmacol..

[438] Tanik S.A., Schultheiss C.E., Volpicelli-Daley L.A., Brunden K.R., Lee V.M. (2013). Lewy body-like alpha-synuclein aggregates resist degradation and impair macroautophagy. J. Biol. Chem..

[439] Roos T.T., Garcia M.G., Martinsson I., Mabrouk R., Israelsson B., Deierborg T., Kobro-Flatmoen A., Tanila H., Gouras G.K. (2021). Neuronal spreading and plaque induction of intracellular Abeta and its disruption of Abeta homeostasis. Acta Neuropathol..

[440] Nabizadeh F. (2024). Disruption in functional networks mediated tau spreading in Alzheimer’s disease. Brain Commun..

[441] Hussain R., Zubair H., Pursell S., Shahab M. (2018). Neurodegenerative Diseases: Regenerative Mechanisms and Novel Therapeutic Approaches. Brain Sci..

[442] Agnello L., Ciaccio M. (2022). Neurodegenerative Diseases: From Molecular Basis to Therapy. Int. J. Mol. Sci..

[443] Hurtado-Alvarado G., Dominguez-Salazar E., Velazquez-Moctezuma J., Gomez-Gonzalez B. (2016). A2A Adenosine Receptor Antagonism Reverts the Blood-Brain Barrier Dysfunction Induced by Sleep Restriction. PLoS ONE.

[444] Mishra A., Bandopadhyay R., Singh P.K., Mishra P.S., Sharma N., Khurana N. (2021). Neuroinflammation in neurological disorders: Pharmacotherapeutic targets from bench to bedside. Metab. Brain Dis..

[445] Candelario-Jalil E., Gonzalez-Falcon A., Garcia-Cabrera M., Leon O.S., Fiebich B.L. (2007). Post-ischaemic treatment with the cyclooxygenase-2 inhibitor nimesulide reduces blood-brain barrier disruption and leukocyte infiltration following transient focal cerebral ischaemia in rats. J. Neurochem..

[446] Candelario-Jalil E., Taheri S., Yang Y., Sood R., Grossetete M., Estrada E.Y., Fiebich B.L., Rosenberg G.A. (2007). Cyclooxygenase inhibition limits blood-brain barrier disruption following intracerebral injection of tumor necrosis factor-alpha in the rat. J. Pharmacol. Exp. Ther..

[447] Sharif A., Mamo J., Lam V., Al-Salami H., Mooranian A., Watts G.F., Clarnette R., Luna G., Takechi R. (2024). The therapeutic potential of probucol and probucol analogues in neurodegenerative diseases. Transl. Neurodegener..

[448] Ahmadi M., Bekeschus S., Weltmann K.D., von Woedtke T., Wende K. (2022). Non-steroidal anti-inflammatory drugs: Recent advances in the use of synthetic COX-2 inhibitors. RSC Med. Chem..

[449] Bilal W., Khawar M.B., Afzal A., Naseer A., Hamid S.E., Shahzaman S., Qamar F. (2023). Recent advances to Neuroprotection: Repurposing drugs against neuroinflammatory disorders. Mol. Biol. Rep..

[450] Mendiola A.S., Cardona A.E. (2018). The IL-1beta phenomena in neuroinflammatory diseases. J. Neural Transm..

[451] Gaire B.P. (2022). Microglia as the Critical Regulators of Neuroprotection and Functional Recovery in Cerebral Ischemia. Cell. Mol. Neurobiol..

[452] Carta A.R., Pisanu A. (2013). Modulating microglia activity with PPAR-gamma agonists: A promising therapy for Parkinson’s disease?. Neurotox. Res..

[453] Liu C.Y., Wang X., Liu C., Zhang H.L. (2019). Pharmacological Targeting of Microglial Activation: New Therapeutic Approach. Front. Cell. Neurosci..

[454] Pearson A., Koprivica M., Eisenbaum M., Ortiz C., Browning M., Vincennie T., Tinsley C., Mullan M., Crawford F., Ojo J. (2024). PPARgamma activation ameliorates cognitive impairment and chronic microglial activation in the aftermath of r-mTBI. J. Neuroinflamm..

[455] Richardson P.J., Smith D.P., de Giorgio A., Snetkov X., Almond-Thynne J., Cronin S., Mead R.J., McDermott C.J., Shaw P.J. (2023). Janus kinase inhibitors are potential therapeutics for amyotrophic lateral sclerosis. Transl. Neurodegener..

[456] Faquetti M.L., Slappendel L., Bigonne H., Grisoni F., Schneider P., Aichinger G., Schneider G., Sturla S.J., Burden A.M. (2024). Baricitinib and tofacitinib off-target profile, with a focus on Alzheimer’s disease. Alzheimer’s Dement. Transl. Res. Clin. Interv..

[457] Tak P.P., Firestein G.S. (2001). NF-kappaB: A key role in inflammatory diseases. J. Clin. Investig..

[458] Wang Z., Xue Y., Jiao H., Liu Y., Wang P. (2012). Doxycycline-mediated protective effect against focal cerebral ischemia-reperfusion injury through the modulation of tight junctions and PKCdelta signaling in rats. J. Mol. Neurosci..

[459] Robinson B.D., Isbell C.L., Melge A.R., Lomas A.M., Shaji C.A., Mohan C.G., Huang J.H., Tharakan B. (2022). Doxycycline prevents blood-brain barrier dysfunction and microvascular hyperpermeability after traumatic brain injury. Sci. Rep..

[460] Kim Y., Cho A.Y., Kim H.C., Ryu D., Jo S.A., Jung Y.S. (2022). Effects of Natural Polyphenols on Oxidative Stress-Mediated Blood-Brain Barrier Dysfunction. Antioxidants.

[461] Delanty N., Dichter M.A. (2000). Antioxidant therapy in neurologic disease. Arch. Neurol..

[462] Ashok A., Andrabi S.S., Mansoor S., Kuang Y., Kwon B.K., Labhasetwar V. (2022). Antioxidant Therapy in Oxidative Stress-Induced Neurodegenerative Diseases: Role of Nanoparticle-Based Drug Delivery Systems in Clinical Translation. Antioxidants.

[463] Franzoni F., Scarfo G., Guidotti S., Fusi J., Asomov M., Pruneti C. (2021). Oxidative Stress and Cognitive Decline: The Neuroprotective Role of Natural Antioxidants. Front. Neurosci..

[464] Feng J., Zheng Y., Guo M., Ares I., Martinez M., Lopez-Torres B., Martinez-Larranaga M.R., Wang X., Anadon A., Martinez M.A. (2023). Oxidative stress, the blood-brain barrier and neurodegenerative diseases: The critical beneficial role of dietary antioxidants. Acta Pharm. Sin. B.

[465] Faysal M., Dehbia Z., Zehravi M., Sweilam S.H., Haque M.A., Kumar K.P., Chakole R.D., Shelke S.P., Sirikonda S., Nafady M.H. (2024). Flavonoids as Potential Therapeutics Against Neurodegenerative Disorders: Unlocking the Prospects. Neurochem. Res..

[466] Senol N., Naziroglu M., Yuruker V. (2014). N-acetylcysteine and selenium modulate oxidative stress, antioxidant vitamin and cytokine values in traumatic brain injury-induced rats. Neurochem. Res..

[467] Sahasrabudhe S.A., Terluk M.R., Kartha R.V. (2023). N-acetylcysteine Pharmacology and Applications in Rare Diseases-Repurposing an Old Antioxidant. Antioxidants.

[468] Mule S., Ferrari S., Rosso G., Brovero A., Botta M., Congiusta A., Galla R., Molinari C., Uberti F. (2024). The Combined Antioxidant Effects of N-Acetylcysteine, Vitamin D3, and Glutathione from the Intestinal-Neuronal In Vitro Model. Foods.

[469] Pham T., MacRae C.L., Broome S.C., D’Souza R.F., Narang R., Wang H.W., Mori T.A., Hickey A.J.R., Mitchell C.J., Merry T.L. (2020). MitoQ and CoQ10 supplementation mildly suppresses skeletal muscle mitochondrial hydrogen peroxide levels without impacting mitochondrial function in middle-aged men. Eur. J. Appl. Physiol..

[470] Wainwright L., Hargreaves I.P., Georgian A.R., Turner C., Dalton R.N., Abbott N.J., Heales S.J.R., Preston J.E. (2020). CoQ(10) Deficient Endothelial Cell Culture Model for the Investigation of CoQ(10) Blood-Brain Barrier Transport. J. Clin. Med..

[471] Rudrapal M., Khairnar S.J., Khan J., Dukhyil A.B., Ansari M.A., Alomary M.N., Alshabrmi F.M., Palai S., Deb P.K., Devi R. (2022). Dietary Polyphenols and Their Role in Oxidative Stress-Induced Human Diseases: Insights Into Protective Effects, Antioxidant Potentials and Mechanism(s) of Action. Front. Pharmacol..

[472] Winiarska-Mieczan A., Kwiecien M., Jachimowicz-Rogowska K., Donaldson J., Tomaszewska E., Baranowska-Wojcik E. (2023). Anti-Inflammatory, Antioxidant, and Neuroprotective Effects of Polyphenols-Polyphenols as an Element of Diet Therapy in Depressive Disorders. Int. J. Mol. Sci..

[473] Purgatorio R., Boccarelli A., Pisani L., de Candia M., Catto M., Altomare C.D. (2024). A Critical Appraisal of the Protective Activity of Polyphenolic Antioxidants against Iatrogenic Effects of Anticancer Chemotherapeutics. Antioxidants.

[474] Figueira I., Tavares L., Jardim C., Costa I., Terrasso A.P., Almeida A.F., Govers C., Mes J.J., Gardner R., Becker J.D. (2019). Blood-brain barrier transport and neuroprotective potential of blackberry-digested polyphenols: An in vitro study. Eur. J. Nutr..

[475] Revi N., Rengan A.K. (2021). Impact of dietary polyphenols on neuroinflammation-associated disorders. Neurol. Sci..

[476] Singhal A., Morris V.B., Labhasetwar V., Ghorpade A. (2013). Nanoparticle-mediated catalase delivery protects human neurons from oxidative stress. Cell Death Dis..

[477] Zhao H., Zhang R., Yan X., Fan K. (2021). Superoxide dismutase nanozymes: An emerging star for anti-oxidation. J. Mater. Chem. B.

[478] Chen Y., Li B., Li K., Lin Y. (2024). Superoxide dismutase nanozymes: Current status and future perspectives on brain disease treatment and diagnosis. Chem. Commun..

[479] Scuderi S.A., Ardizzone A., Paterniti I., Esposito E., Campolo M. (2020). Antioxidant and Anti-inflammatory Effect of Nrf2 Inducer Dimethyl Fumarate in Neurodegenerative Diseases. Antioxidants.

[480] Manai F., Amadio M. (2022). Dimethyl Fumarate Triggers the Antioxidant Defense System in Human Retinal Endothelial Cells through Nrf2 Activation. Antioxidants.

[481] Sabharwal A.K., May J.M. (2008). alpha-Lipoic acid and ascorbate prevent LDL oxidation and oxidant stress in endothelial cells. Mol. Cell. Biochem..

[482] Badran M., Abuyassin B., Golbidi S., Ayas N., Laher I. (2019). Alpha Lipoic Acid Improves Endothelial Function and Oxidative Stress in Mice Exposed to Chronic Intermittent Hypoxia. Oxidative Med. Cell. Longev..

[483] Wang W., An L.P., Li Y.F., An R., Bian Z., Liu W.Z., Song Q.H., Li A.Y. (2020). Alpha-lipoic acid ameliorates H(2)O(2)-induced human vein endothelial cells injury via suppression of inflammation and oxidative stress. Biosci. Biotechnol. Biochem..

[484] de la Mata M., Cotan D., Oropesa-Avila M., Garrido-Maraver J., Cordero M.D., Villanueva Paz M., Delgado Pavon A., Alcocer-Gomez E., de Lavera I., Ybot-Gonzalez P. (2015). Pharmacological Chaperones and Coenzyme Q10 Treatment Improves Mutant beta-Glucocerebrosidase Activity and Mitochondrial Function in Neuronopathic Forms of Gaucher Disease. Sci. Rep..

[485] Voronin M.V., Abramova E.V., Verbovaya E.R., Vakhitova Y.V., Seredenin S.B. (2023). Chaperone-Dependent Mechanisms as a Pharmacological Target for Neuroprotection. Int. J. Mol. Sci..

[486] Choi E.H., Kim M.H., Park S.J. (2024). Targeting Mitochondrial Dysfunction and Reactive Oxygen Species for Neurodegenerative Disease Treatment. Int. J. Mol. Sci..

[487] Peng Y., Ao M., Dong B., Jiang Y., Yu L., Chen Z., Hu C., Xu R. (2021). Anti-Inflammatory Effects of Curcumin in the Inflammatory Diseases: Status, Limitations and Countermeasures. Drug Des. Dev. Ther..

[488] Forster C., Burek M., Romero I.A., Weksler B., Couraud P.O., Drenckhahn D. (2008). Differential effects of hydrocortisone and TNFalpha on tight junction proteins in an in vitro model of the human blood-brain barrier. J. Physiol..

[489] Kroll S., El-Gindi J., Thanabalasundaram G., Panpumthong P., Schrot S., Hartmann C., Galla H.J. (2009). Control of the blood-brain barrier by glucocorticoids and the cells of the neurovascular unit. Ann. N. Y. Acad. Sci..

[490] Salvador E., Shityakov S., Forster C. (2014). Glucocorticoids and endothelial cell barrier function. Cell Tissue Res..

[491] Chen X., Ghribi O., Geiger J.D. (2010). Caffeine protects against disruptions of the blood-brain barrier in animal models of Alzheimer’s and Parkinson’s diseases. J. Alzheimer’s Dis..

[492] Osz B.E., Jitca G., Stefanescu R.E., Puscas A., Tero-Vescan A., Vari C.E. (2022). Caffeine and Its Antioxidant Properties-It Is All about Dose and Source. Int. J. Mol. Sci..

[493] Ruggiero M., Calvello R., Porro C., Messina G., Cianciulli A., Panaro M.A. (2022). Neurodegenerative Diseases: Can Caffeine Be a Powerful Ally to Weaken Neuroinflammation?. Int. J. Mol. Sci..

[494] Lange C., Storkebaum E., de Almodovar C.R., Dewerchin M., Carmeliet P. (2016). Vascular endothelial growth factor: A neurovascular target in neurological diseases. Nat. Rev. Neurol..

[495] Ali M., Falkenhain K., Njiru B.N., Murtaza-Ali M., Ruiz-Uribe N.E., Haft-Javaherian M., Catchers S., Nishimura N., Schaffer C.B., Bracko O. (2022). VEGF signalling causes stalls in brain capillaries and reduces cerebral blood flow in Alzheimer’s mice. Brain.

[496] Zhang M., Zhang Z., Li H., Xia Y., Xing M., Xiao C., Cai W., Bu L., Li Y., Park T.E. (2024). Blockage of VEGF function by bevacizumab alleviates early-stage cerebrovascular dysfunction and improves cognitive function in a mouse model of Alzheimer’s disease. Transl. Neurodegener..

[497] Yu S., Chen X., Yang T., Cheng J., Liu E., Jiang L., Song M., Shu H., Ma Y. (2024). Revealing the mechanisms of blood-brain barrier in chronic neurodegenerative disease: An opportunity for therapeutic intervention. Rev. Neurosci..

[498] Wang Q., Huang X., Su Y., Yin G., Wang S., Yu B., Li H., Qi J., Chen H., Zeng W. (2022). Activation of Wnt/beta-catenin pathway mitigates blood-brain barrier dysfunction in Alzheimer’s disease. Brain.

[499] Lee W., Ku S.K., Bae J.S. (2015). Anti-inflammatory effects of Baicalin, Baicalein, and Wogonin in vitro and in vivo. Inflammation.

[500] Li M.T., Ke J., Guo S.F., Wu Y., Bian Y.F., Shan L.L., Liu Q.Y., Huo Y.J., Guo C., Liu M.Y. (2021). The Protective Effect of Quercetin on Endothelial Cells Injured by Hypoxia and Reoxygenation. Front. Pharmacol..

[501] Wen Y., Wang Y., Zhao C., Zhao B., Wang J. (2023). The Pharmacological Efficacy of Baicalin in Inflammatory Diseases. Int. J. Mol. Sci..

[502] Kaur J., Fahmy L.M., Davoodi-Bojd E., Zhang L., Ding G., Hu J., Zhang Z., Chopp M., Jiang Q. (2021). Waste Clearance in the Brain. Front. Neuroanat..

[503] Hartz A.M., Miller D.S., Bauer B. (2010). Restoring blood-brain barrier P-glycoprotein reduces brain amyloid-beta in a mouse model of Alzheimer’s disease. Mol. Pharmacol..

[504] van Assema D.M., Lubberink M., Bauer M., van der Flier W.M., Schuit R.C., Windhorst A.D., Comans E.F., Hoetjes N.J., Tolboom N., Langer O. (2012). Blood-brain barrier P-glycoprotein function in Alzheimer’s disease. Brain.

[505] Singh N., Ecker G.F. (2018). Insights into the Structure, Function, and Ligand Discovery of the Large Neutral Amino Acid Transporter 1, LAT1. Int. J. Mol. Sci..

[506] Liu Q., Wu D., Ni N., Ren H., Luo C., He C., Kang J.X., Wan J.B., Su H. (2014). Omega-3 polyunsaturated fatty acids protect neural progenitor cells against oxidative injury. Mar. Drugs.

[507] Kang T., Han Z., Zhu L., Cao B. (2024). TFR1 knockdown alleviates iron overload and mitochondrial dysfunction during neural differentiation of Alzheimer’s disease-derived induced pluripotent stem cells by interacting with GSK3B. Eur. J. Med. Res..

[508] Malau I.A., Chang J.P., Lin Y.W., Chang C.C., Chiu W.C., Su K.P. (2024). Omega-3 Fatty Acids and Neuroinflammation in Depression: Targeting Damage-Associated Molecular Patterns and Neural Biomarkers. Cells.

[509] Hartz A.M., Bauer B., Fricker G., Miller D.S. (2006). Rapid modulation of P-glycoprotein-mediated transport at the blood-brain barrier by tumor necrosis factor-alpha and lipopolysaccharide. Mol. Pharmacol..

[510] Miller D.S., Bauer B., Hartz A.M. (2008). Modulation of P-glycoprotein at the blood-brain barrier: Opportunities to improve central nervous system pharmacotherapy. Pharmacol. Rev..

[511] Liu Q., Hou J., Chen X., Liu G., Zhang D., Sun H., Zhang J. (2014). P-glycoprotein mediated efflux limits the transport of the novel anti-Parkinson’s disease candidate drug FLZ across the physiological and PD pathological in vitro BBB models. PLoS ONE.

[512] Gu Z.F., Hao Y.D., Wang T.Y., Cai P.L., Zhang Y., Deng K.J., Lin H., Lv H. (2024). Prediction of blood-brain barrier penetrating peptides based on data augmentation with Augur. BMC Biol..

[513] Parker A., Fonseca S., Carding S.R. (2020). Gut microbes and metabolites as modulators of blood-brain barrier integrity and brain health. Gut Microbes.

[514] Fock E., Parnova R. (2023). Mechanisms of Blood-Brain Barrier Protection by Microbiota-Derived Short-Chain Fatty Acids. Cells.

[515] Motataianu A., Serban G., Andone S. (2023). The Role of Short-Chain Fatty Acids in Microbiota-Gut-Brain Cross-Talk with a Focus on Amyotrophic Lateral Sclerosis: A Systematic Review. Int. J. Mol. Sci..

[516] Zheng X., Yang J., Hou Y., Fang Y., Wu K., Song Y., Liu K., Zhu J. (2023). Current non-invasive strategies for brain drug delivery: Overcoming blood-brain barrier transport. Mol. Biol. Rep..

[517] Mallah K., Couch C., Borucki D.M., Toutonji A., Alshareef M., Tomlinson S. (2020). Anti-inflammatory and Neuroprotective Agents in Clinical Trials for CNS Disease and Injury: Where Do We Go From Here?. Front. Immunol..

[518] Villapol S., Saavedra J.M. (2015). Neuroprotective effects of angiotensin receptor blockers. Am. J. Hypertens..

[519] Welcome M.O. (2020). Cellular mechanisms and molecular signaling pathways in stress-induced anxiety, depression, and blood-brain barrier inflammation and leakage. Inflammopharmacology.

[520] Saleh N., Cosarderelioglu C., Vajapey R., Walston J., Abadir P.M. (2022). Losartan Mitigates Oxidative Stress in the Brains of Aged and Inflamed IL-10-/- Mice. J. Gerontol. A Biol. Sci. Med. Sci..

[521] Shepardson N.E., Shankar G.M., Selkoe D.J. (2011). Cholesterol level and statin use in Alzheimer disease: II. Review of human trials and recommendations. Arch. Neurol..

[522] Shepardson N.E., Shankar G.M., Selkoe D.J. (2011). Cholesterol level and statin use in Alzheimer disease: I. Review of epidemiological and preclinical studies. Arch. Neurol..

[523] Schultz B.G., Patten D.K., Berlau D.J. (2018). The role of statins in both cognitive impairment and protection against dementia: A tale of two mechanisms. Transl. Neurodegener..

[524] McCarthy M., Raval A.P. (2020). The peri-menopause in a woman’s life: A systemic inflammatory phase that enables later neurodegenerative disease. J. Neuroinflamm..

[525] Zhang Y., Tan X., Tang C. (2024). Estrogen-immuno-neuromodulation disorders in menopausal depression. J. Neuroinflamm..

[526] Craig C.F., Filippone R.T., Stavely R., Bornstein J.C., Apostolopoulos V., Nurgali K. (2022). Neuroinflammation as an etiological trigger for depression comorbid with inflammatory bowel disease. J. Neuroinflamm..

[527] Duan L., Li X., Ji R., Hao Z., Kong M., Wen X., Guan F., Ma S. (2023). Nanoparticle-Based Drug Delivery Systems: An Inspiring Therapeutic Strategy for Neurodegenerative Diseases. Polymers.

[528] Zhou N., Gu T., Xu Y., Liu Y., Peng L. (2023). Challenges and progress of neurodrug: Bioactivities, production and delivery strategies of nerve growth factor protein. J. Biol. Eng..

[529] Kumari S., Kamiya A., Karnik S.S., Rohilla S., Dubey S.K., Taliyan R. (2025). Novel Gene Therapy Approaches for Targeting Neurodegenerative Disorders: Focusing on Delivering Neurotrophic Genes. Mol. Neurobiol..

[530] Kowalczyk A., Kleniewska P., Kolodziejczyk M., Skibska B., Goraca A. (2015). The role of endothelin-1 and endothelin receptor antagonists in inflammatory response and sepsis. Arch Immunol Ther. Exp..

[531] Enevoldsen F.C., Sahana J., Wehland M., Grimm D., Infanger M., Kruger M. (2020). Endothelin Receptor Antagonists: Status Quo and Future Perspectives for Targeted Therapy. J. Clin. Med..

[532] van Doorn R., Nijland P.G., Dekker N., Witte M.E., Lopes-Pinheiro M.A., van het Hof B., Kooij G., Reijerkerk A., Dijkstra C., van van der Valk P. (2012). Fingolimod attenuates ceramide-induced blood-brain barrier dysfunction in multiple sclerosis by targeting reactive astrocytes. Acta Neuropathol..

[533] Nishihara H., Shimizu F., Sano Y., Takeshita Y., Maeda T., Abe M., Koga M., Kanda T. (2015). Fingolimod prevents blood-brain barrier disruption induced by the sera from patients with multiple sclerosis. PLoS ONE.

[534] Wang Z., Kawabori M., Houkin K. (2020). FTY720 (Fingolimod) Ameliorates Brain Injury through Multiple Mechanisms and is a Strong Candidate for Stroke Treatment. Curr. Med. Chem..

[535] Zhao W., Xu Z., Cao J., Fu Q., Wu Y., Zhang X., Long Y., Zhang X., Yang Y., Li Y. (2019). Elamipretide (SS-31) improves mitochondrial dysfunction, synaptic and memory impairment induced by lipopolysaccharide in mice. J. Neuroinflamm..

[536] Grosser J.A., Fehrman R.L., Keefe D., Redmon M., Nickells R.W. (2021). The effects of a mitochondrial targeted peptide (elamipretide/SS31) on BAX recruitment and activation during apoptosis. BMC Res. Notes.

[537] Cheng F., Liu J., Guo Z., Li S., Chen J., Tu C., Fu F., Shen B., Zhang X., Lai G. (2022). Angiotensin-(1-7) ameliorates high glucose-induced vascular endothelial injury through suppressing chloride channel 3. Bioengineered.

[538] Bernatoniene J., Kopustinskiene D.M. (2018). The Role of Catechins in Cellular Responses to Oxidative Stress. Molecules.

[539] Aghababaei F., Hadidi M. (2023). Recent Advances in Potential Health Benefits of Quercetin. Pharmaceuticals.

[540] Sheng Y., Sun Y., Tang Y., Yu Y., Wang J., Zheng F., Li Y., Sun Y. (2023). Catechins: Protective mechanism of antioxidant stress in atherosclerosis. Front. Pharmacol..

[541] Qu Z., Luo J., Li Z., Yang R., Zhao J., Chen X., Yu S., Shu H. (2024). Advancements in strategies for overcoming the blood-brain barrier to deliver brain-targeted drugs. Front. Aging Neurosci..

[542] Chen C.C., Sheeran P.S., Wu S.Y., Olumolade O.O., Dayton P.A., Konofagou E.E. (2013). Targeted drug delivery with focused ultrasound-induced blood-brain barrier opening using acoustically-activated nanodroplets. J. Control. Release.

[543] Fan C.H., Liu H.L., Ting C.Y., Lee Y.H., Huang C.Y., Ma Y.J., Wei K.C., Yen T.C., Yeh C.K. (2014). Submicron-bubble-enhanced focused ultrasound for blood-brain barrier disruption and improved CNS drug delivery. PLoS ONE.

[544] Wasielewska J.M., White A.R. (2022). Focused Ultrasound-mediated Drug Delivery in Humans—A Path Towards Translation in Neurodegenerative Diseases. Pharm. Res..

[545] Balasubramanian P., Delfavero J., Nyul-Toth A., Tarantini A., Gulej R., Tarantini S. (2021). Integrative Role of Hyperbaric Oxygen Therapy on Healthspan, Age-Related Vascular Cognitive Impairment, and Dementia. Front. Aging.

[546] Mensah-Kane P., Sumien N. (2023). The potential of hyperbaric oxygen as a therapy for neurodegenerative diseases. Geroscience.

[547] Zeng H., Zeng D., Yin X., Zhang W., Wu M., Chen Z. (2024). Research progress on high-concentration oxygen therapy after cerebral hemorrhage. Front. Neurol..

[548] Matsui T., Kawahara N., Kimoto A., Yoshida Y. (2015). Hypothermia Reduces but Hyperthermia Augments T Cell-Derived Release of Interleukin-17 and Granzyme B that Mediate Neuronal Cell Death. Neurocrit. Care.

[549] You J.S., Kim J.Y., Yenari M.A. (2022). Therapeutic hypothermia for stroke: Unique challenges at the bedside. Front. Neurol..

[550] Xu W., Geng X., Fayyaz A.I., Ding Y. (2024). The Modulatory Role of Hypothermia in Poststroke Brain Inflammation: Mechanisms and Clinical Implications. Cerebrovasc. Dis..

[551] Salford L.G., Brun A., Sturesson K., Eberhardt J.L., Persson B.R. (1994). Permeability of the blood-brain barrier induced by 915 MHz electromagnetic radiation, continuous wave and modulated at 8, 16, 50, and 200 Hz. Microsc. Res. Tech..

[552] Xing Y., Liang G., Zhu T. (2022). Current status and outlook of potential applications of biodegradable materials in cerebral vascular stents. Neurosurg. Rev..

[553] Boulingre M., Portillo-Lara R., Green R.A. (2023). Biohybrid neural interfaces: Improving the biological integration of neural implants. Chem. Commun..

